# Copper(I)-catalyzed tandem reaction: synthesis of 1,4-disubstituted 1,2,3-triazoles from alkyl diacyl peroxides, azidotrimethylsilane, and alkynes

**DOI:** 10.3762/bjoc.14.270

**Published:** 2018-11-23

**Authors:** Muhammad Israr, Changqing Ye, Munira Taj Muhammad, Yajun Li, Hongli Bao

**Affiliations:** 1 Key Laboratory of Coal to Ethylene Glycol and Its Related Technology, State Key Laboratory of Structural Chemistry, Fujian Institute of Research on the Structure of Matter, Center for Excellence in Molecular Synthesis, Chinese Academy of Sciences, 155 Yangqiao Road West, Fuzhou, Fujian 350002, P. R. China; 2 University of Chinese academy of Science (UCAS), Beijing 100190, P. R. China

**Keywords:** alkyl diacyl peroxides, azidotrimethylsilane, click reaction, copper catalysis, radical, 1,2,3-triazoles

## Abstract

A copper-catalyzed azide–alkyne cycloaddition (CuAAC) reaction for the synthesis of 1,4-disubstituted 1,2,3-triazoles from alkyl diacyl peroxides, azidotrimethylsilane, and terminal alkynes is reported. The alkyl carboxylic acids is for the first time being used as the alkyl azide precursors in the form of alkyl diacyl peroxides. This method avoids the necessity to handle organic azides, as they are generated in situ, making this protocol operationally simple. The Cu(I) catalyst not only participates in the alkyl diacyl peroxides decomposition to afford alkyl azides but also catalyzes the subsequent CuAAC reaction to produce the 1,2,3-triazoles.

## Introduction

The “click chemistry”, coined by K. B. Sharpless in 2001 [1], is a powerful chemical transformation that has rapidly orthogonalized traditional disciplinary boundaries. With the discovery of “click chemistry”, new fields have been opened for the research and synthesis of functionalized compounds that have applications in medicinal chemistry, drug discovery, materials chemistry, and as well as in bioconjugates [2–12].

**Scheme 1:**
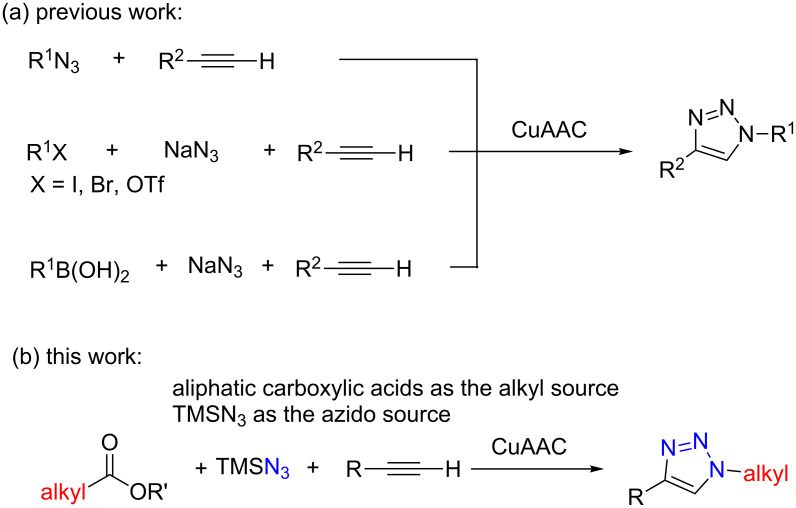
General methods for the synthesis of triazoles.

The copper-catalyzed azide–alkyne cycloaddition (CuAAC) reaction [13–21], derived from the Huisgen’s 1,3-dipolar cycloaddition of azides and alkynes [22], has conceivably emerged as the premier example of click chemistry. Generally, organic azides are used as the azido source in most of the CuAAC reactions (Scheme 1a) [23]. However, the organic azides with low molecular weight are considered to be unstable moieties that can decompose spontaneously, with which the reactions are difficult or dangerous to handle [16]. Thus, a one-pot two-step process for the in situ generation of organic azides is highly required. A frequently used method to in situ generate organic azides is the azidation of organic halides, such as aliphatic halides, vinyl halides, or aromatic halides with sodium azide [24–27]. Organic triflates [28] and organic boronic acids [29–31] can also be used as alternative precursors for organic azides, when reacted with sodium azide.

However, sodium azide is a highly toxic compound and has the potential to explode. Azidotrimethylsilane (TMSN_3_) has been considered as a safer azide source, which actually has been successfully used in the CuAAC reaction directly [32–35], but rarely been used as an azido precursor to enrich the functionality of organic azide source. Moreover, as one of the most commonly appearing compounds in nature, carboxylic acids have rarely been directly used as the organic azide precursors for CuAAC reactions, considering the frequent involvement of organic halides. Thus, new methods with non or less toxic reagents and enriched organic azide sources for CuAAC reaction are still highly required.

Herein, we report a novel CuAAC reaction, using aliphatic carboxylic acids as the alkyl source [36], and TMSN_3_ as the azide source (Scheme 1b). Because TMSN_3_ can react with alkynes to form the CuAAC reaction product [32–35], there is one significant challenge of this method that need to be emphasized: how to control the reaction to generate the alkyl azides from aliphatic carboxylic acids and TMSN_3_, before TMSN_3_ directly reacting with alkynes.

## Results and Discussion

Based on our unpublished work, we found that alkyl azide has always appeared as a side product when the reaction involved TMSN_3_ and alkyl diacyl peroxide, easily available compounds derived from aliphatic carboxylic acids. With this information in mind, initially, we started our investigation with phenylacetylene (**1a**), commercially available lauroyl peroxide (**2a**), and TMSN_3_ (Table 1). In a preliminary experiment, the reaction of **1a** with **2a** in the presence of 10 mol % of CuCl in THF at 50 °C afforded 1,4-disubstituted 1,2,3-triazole **3a** in 65% isolated yield (Table 1, entry 1). Surprisingly, under these conditions no CuAAC product between TMSN_3_ and phenylacetylene was detected. This result encouraged us to further exploit the optimization of the reaction conditions. Afterwards, the effect of the solvent was also investigated (Table 1, entries 2−8). Dichloromethane could afford the best result and the yield of the desired product **3a** could be as high as 97% (Table 1, entry 2). Other metal salts of copper, such as Cu(OAc)_2_, CuI, and CuBr were then examined and they showed lower catalytic efficiencies than that of CuCl (Table 1, entries 9−11). Moreover, a reduced amount of the catalyst loading leads to lower yields of product **3a** (Table 1, entries 12–14).

**Table 1:** Optimization of the reaction conditions^a^.

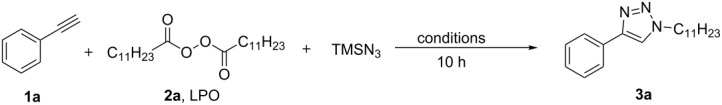			

entry	catalyst (mol %)	solvent	yield (%)^b^

1	CuCl (10)	THF	(65)^c^
2	CuCl (10)	CH_2_Cl_2_	96 (97)^c^
3	CuCl (10)	EtOH	53
4	CuCl (10)	DMF	13
5	CuCl (10)	MeOH	7
6	CuCl (10)	1,4-dioxane	trace
7	CuCl (10)	MeCN	trace
8	CuCl (10)	acetone	trace
9	CuI (10)	CH_2_Cl_2_	52
10	CuBr (10)	CH_2_Cl_2_	48
11	Cu(OAc)_2_ (10)	CH_2_Cl_2_	64
12	CuCl (7.5)	CH_2_Cl_2_	84
13	CuCl (5)	CH_2_Cl_2_	70
14	CuCl (2)	CH_2_Cl_2_	54

^a^Reaction conditions: **1a** (0.5 mmol), **2a** (0.75 mmol), TMSN_3_ (0.75 mmol), catalyst (mol %), solvent (2 mL), 50 °C, 10 h. ^b^Yield was determined by ^1^H NMR analysis. ^c^Isolated yield in parentheses.

**Scheme 2:**
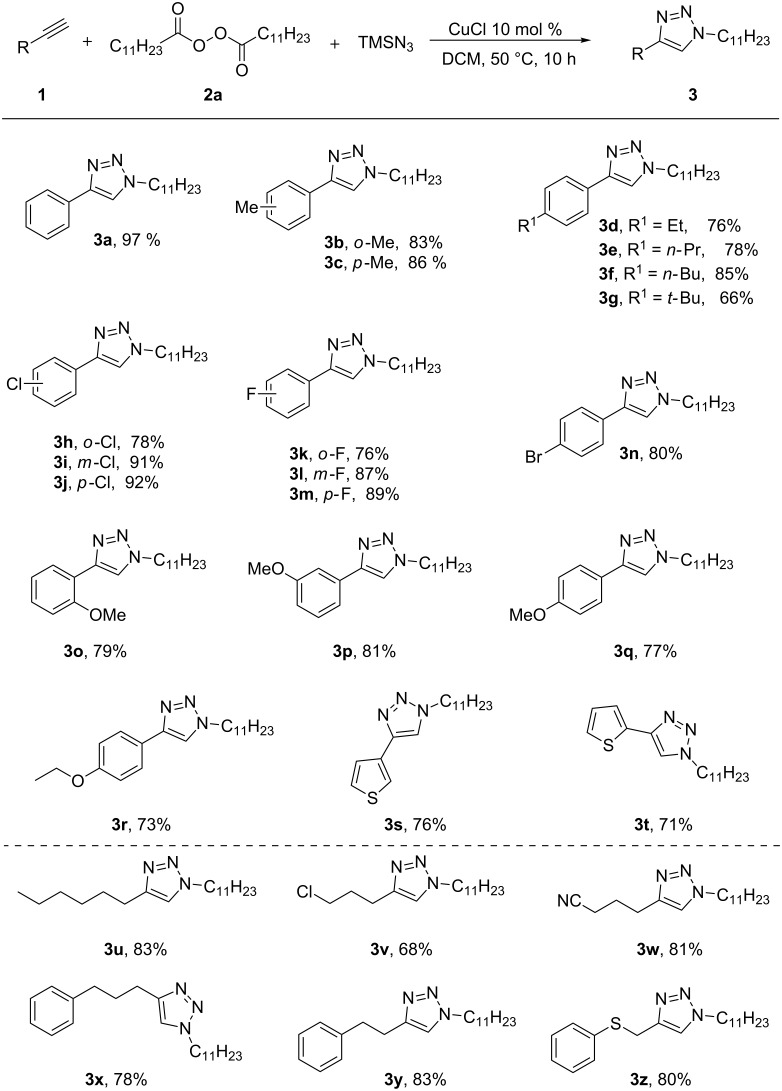
Substrate scope of the terminal alkynes. Conditions: **1** (0.5 mmol), **2a** (0.75 mmol), TMSN_3_ (0.75 mmol), CuCl (10 mol %), DCM (2 mL), 50 °C, 10 h. Yields of the isolated products are given.

With the optimized reaction conditions in hand, the scope of the terminal alkynes was screened and the results are depicted in Scheme 2. First, the reactivity of various substituted terminal arylalkynes was examined. Only 1,4-regioisomeric products were formed with good to excellent yields. Phenylacetylene with an electron-withdrawing bromo-, chloro-, or fluoro substituent afforded the corresponding products **3h**–**n** in up to 92% yield, while phenylacetylenes with electron-donating groups gave the corresponding products **3b**–**g** and **3o**–**q** in up to 86% yield. Instead of a six-membered ring, five-membered heteroaromatics (ethynylthiophenes) have also been used, and afforded the desired products **3s** and **3t** in up to 76% yield. Terminal aliphatic alkynes were then examined and it was found that they could smoothly deliver the corresponding 1,2,3-triazoles **3u**–**z** with high yields.

**Scheme 3:**
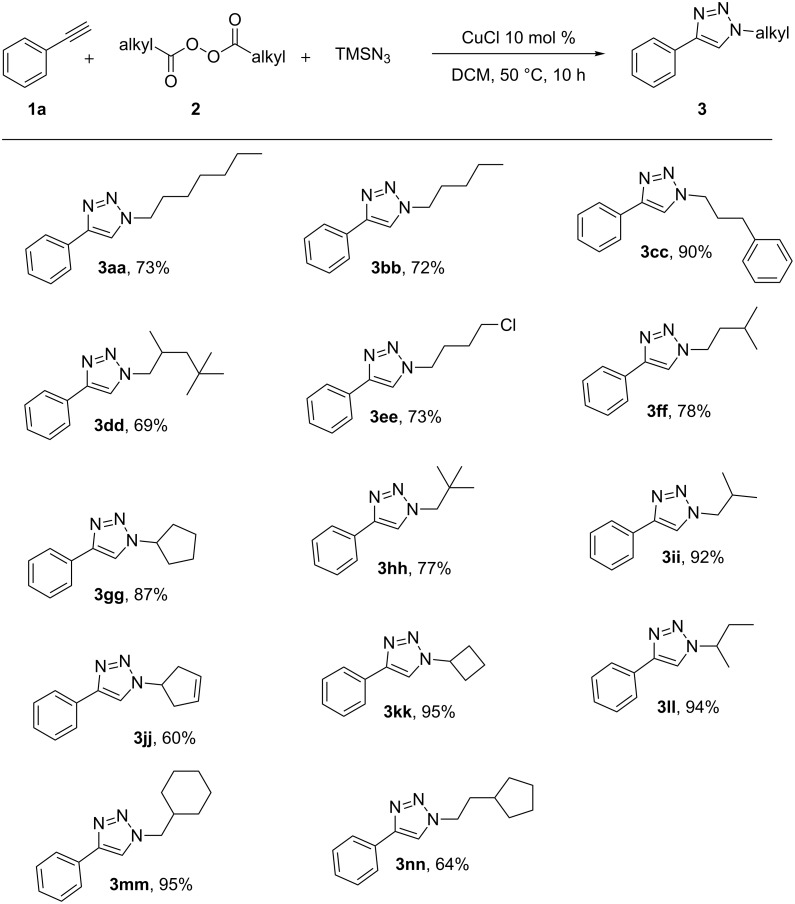
Substrate scope of the alkyl diacyl peroxides. Conditions: **1a** (0.5 mmol), **2** (0.75 mmol), TMSN_3_ (0.75 mmol), CuCl (10 mol %), DCM (2 mL), 50 °C, 10 h. Yields of the isolated products are given.

Furthermore, the scope of the alkyl diacyl peroxides was then studied (Scheme 3). The alkyl diacyl peroxides **2** were synthesized from the corresponding aliphatic carboxylic acids in a single step by DCC-mediated dehydrative condensation with hydrogen peroxide, and were used directly after simple filtration without further treatment; see Supporting Information File 1 for details [37]. The alkyl diacyl peroxides with long-chain alkyl groups and methyl-substituted long-chain alkyl groups afforded the corresponding 1,4-disubstituted 1,2,3-triazoles with good to excellent yields (**3aa**, **3bb**, **3dd**, **3ff**, **3hh**, **3ii**, and **3ll**). Remarkably, the chlorodiacyl peroxide also tolerated the reaction conditions to afford chloro-substituted triazole **3ee** with good yield. Moreover, alkyl diacyl peroxides bearing a phenyl group, cyclopentyl group, or a cyclohexyl group also afforded good yields (**3cc**, **3mm**, and **3nn**). Significantly, diacyl peroxides with cyclic secondary alkanyl and alkenyl groups can also gave the corresponding 1,2,3-triazoles **3gg**, **3jj**, and **3kk**. Tertiary alkyl diacyl peroxides are relatively more reactive than primary and secondary alkyl diacyl peroxides, but they are not stable enough for the simple filtration separation at room temperature. Thus, we have not tried the reactions with tertiary alkyl diacyl peroxides.

**Scheme 4:**
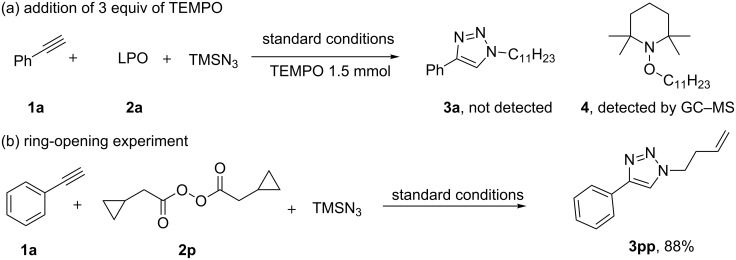
Preliminary mechanistic studies.

In order to understand the mechanism of this reaction, we performed a set of experiments (Scheme 4). Firstly a radical capturing reaction was carried out with the addition of a radical trapping reagent (tetramethylpiperdinyloxy, TEMPO) [38–39] to the standard reaction system, no product **3a** was obtained; only the radical trapped product **4** was detected by GC–MS (Scheme 4a).

To further investigate this phenomenon, we synthesized a substrate bearing a cyclopropylmethyl moiety, diacyl peroxide **2p**, which is a radical-clock [40–41]. The reaction of phenylacetylene with the diacyl peroxide **2p** afforded a ring-opened product **3pp** in 88% yield. This result suggested the engagement of radical species in the reaction (Scheme 4b).

**Scheme 5:**
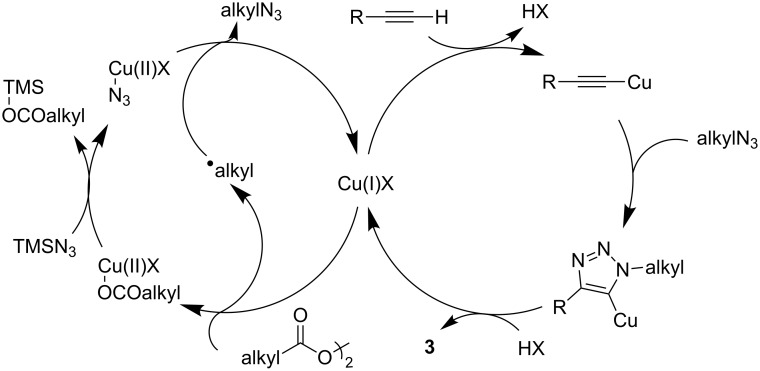
Plausible reaction mechanism.

Based on the previous literature [16,42–43] and the above experimental findings, a possible reaction mechanism is suggested as shown in Scheme 5. In the presence of the Cu(I) catalyst, alkyl diacyl peroxide decomposes into an alkyl radical, CO_2_, and releases a carboxyl–Cu(II) complex, which undergoes a ligand exchange with azidomethylsilane to form azido–Cu(II) species. The alkyl radical then abstracts the azido moiety from the azido–Cu(II) species to afford an alkyl azide and the regenerated Cu(I) catalyst. Then, a conventional CuAAC process delivers the desired cycloaddition product **3**.

## Conclusion

In summary, we have established an efficient, ligand- and additive-free CuAAC reaction for the synthesis of 1,4-disubstituted 1,2,3-triazoles directly from a variety of readily accessible substrates such as alkyl diacyl peroxides, azidotrimethylsilane, and terminal alkynes. The alkyl carboxylic acids are for the first time being used as the alkyl azide precursors in the form of alkyl diacyl peroxides. This method avoids the necessity to handle organic azides, as they are generated in situ, making this protocol operationally simple. This reaction features a wide substrate scope, good functional group tolerance, high yields, and excellent regioselectivity. Most of all, the Cu(I) catalyst plays two roles in the reaction: decomposes the alkyl diacyl peroxides to afford the alkyl azides and catalyzes the subsequent CuAAC reaction to produce the 1,2,3-triazoles.

## Experimental

**General procedure:** To a flame-dried Schlenk tube containing a magnetic stirring bar, terminal alkyne **1** (0.5 mmol), diacyl peroxide **2** (0.75 mmol), TMSN_3_ (90.4 mg, 0.75 mmol), CuCl (4.9 mg, 0.05 mmol) and CH_2_Cl_2_ (2 mL) were added, respectively. The reaction mixture was stirred vigorously for 10 h at 50 °C. Then, the reaction mixture was cooled to room temperature, poured into saturated sodium bicarbonate solution (25 mL) and extracted with CH_2_Cl_2_ (3 × 25 mL). After drying over MgSO_4_, the solvent was removed under reduced pressure in a rotary evaporator; the residue was purified by column chromatography on silica gel (PE/EA) to afford **3**.

## Supporting Information

File 1 Detailed experimental procedures and characterization data for all new compounds.
