# Supplementary material for: Copper(I)-catalyzed tandem reaction: synthesis of 1,4-disubstituted 1,2,3-triazoles from alkyl diacyl peroxides, azidotrimethylsilane, and alkynes
Source: Beilstein J Org Chem. 2018 Nov 23;14:2916–22. doi: 10.3762/bjoc.14.270 (PMC6278767; doi:10.3762/bjoc.14.270)
Supplement: File 1 — Detailed experimental procedures and characterization data for all new compounds. [file Beilstein_J_Org_Chem-14-2916-s001.pdf]

BEILSTEIN JOURNAL OF ORGANIC CHEMISTRY

Supporting Information

for

Copper(I)-catalyzed tandem reaction: synthesis of 1,4-disubstituted 1,2,3-triazoles from alkyl diacyl peroxides, azidotrimethylsilane, and alkynes

Muhammad Israr, Changqing Ye, Munira Taj Muhammad, Yajun Li and Hongli Bao

Beilstein J. Org. Chem. **2018,** *14,* 2916–2922. doi:10.3762/bjoc.14.270

Detailed experimental procedures and characterization data for all new compounds

License and Terms: This is a supporting information file under the terms of the Creative Commons Attribution License (http://creativecommons.org/licenses/by/4.0). Please note that the reuse, redistribution and reproduction in particular requires that the authors and source are credited.
The license is subject to the *Beilstein Journal of Organic Chemistry* terms and conditions: (https://www.beilstein-journals.org/bjoc)

Table of contents

Materials and methods S3

General procedure for the synthesis of triazoles with LPO (for Scheme 2) S4

Synthesis of alkyl diacyl peroxides...S4

General procedure for the synthesis of triazoles with alkyl diacyl peroxides (for Scheme 3) S5

Characterization data for the products S6

Preliminary mechanistic studies S20

References...S22

Copies of ^{1}H, ^{13}C, and ^{19}F NMR spectra S23

Materials and methods

All reactions were carried out in flame-dried glassware with magnetic stirring unless otherwise indicated. Commercially available reagents were used without further purification. Solvents were dried by distillation from sodium or CaH_2. Solutions and liquids were transferred with the help of syringe. Reactions were monitored by thin-layer chromatography. ^{1}H and ^{13}C NMR spectra were recorded on Bruker-BioSpin AVANCE III HD. Data for ^{1}H NMR spectra are reported relative to chloroform as an internal standard (7.26 ppm) and are reported as follows: chemical shift (ppm), multiplicity, coupling constant (Hz), and integration. Data for ^{13}C NMR spectra are reported relative to chloroform as an internal standard (77.16 ppm) and are reported in terms of chemical shift (ppm). All melting points were determined on a Beijing Science Instrument Dianguang Instrument Factory XT4B melting point apparatus and are uncorrected. IR data were obtained from Bruker VERTEX 70. HRMS data were recorded on Agilent Technologies 6224 TOF LC/MS.

General procedure for the synthesis of triazoles with LPO (for Scheme 2)

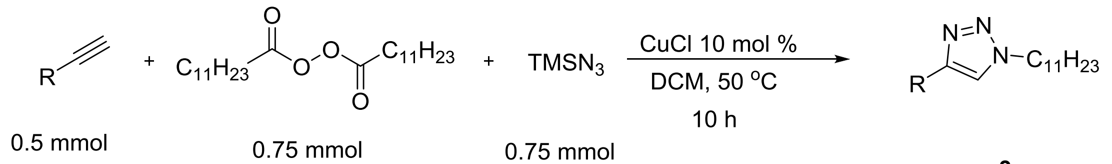

1 2a (LPO) 3

Procedure:

To a flame-dried Schlenk tube containing a magnetic bar, terminal alkyne **1** (0.5 mmol), diacyl peroxide **2a** (0.75 mmol), $TMSN_3$ (90.4 mg, 0.75 mmol), CuCl (4.9 mg, 0.05 mmol) and CH_2Cl_2 (2 mL) were added respectively. The reaction mixture was stirred vigorously for 10 hours at 50 °C. Then, the reaction mixture was cooled to room temperature, poured into saturated sodium bicarbonate solution (25 mL) and extracted with CH_2Cl_2 (3 × 25 mL). After drying over $MgSO_4$, the solvent was removed under reduced pressure by rotary evaporator, the residue was purified by column chromatography on silica gel (PE/EA) to afford **3**.

Synthesis of alkyl diacyl peroxides

Diacyl peroxides were synthesized according to the reported literature.[1,2]

Diacyl peroxides have potentials to explode. Any diacyl peroxide involved in the reaction (as product or substrate) should be carried out with precautions!

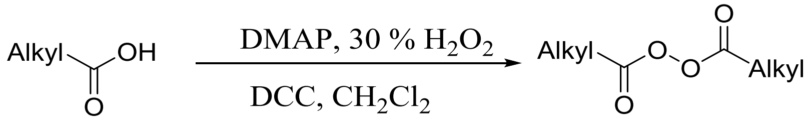

2

4-Dimethylaminopyridine (DMAP, 0.224g, 0.6 mmol), acid (6 mmol), hydrogen peroxide (30 % v/v in H_2O, 7.5 mmol) and CH_2Cl_2 were added to a 50 mL round bottom flask respectively. The reaction mixture was

cooled to −15 °C for about 10 min and dicyclohexylcarbodiimide (DCC, 6.72 mmol) was added. Then, the mixture was stirred at −10 to −15°C for 1.5 hours. The solution was filtered with petroleum ether and dried over $MgSO_4$. After filtration the solvent was removed under reduced pressure at 10 to 15 °C. The residue (peroxide **2**) was used without further purification.

General procedure for the synthesis of triazoles with alkyl diacyl peroxides (for Scheme 3)

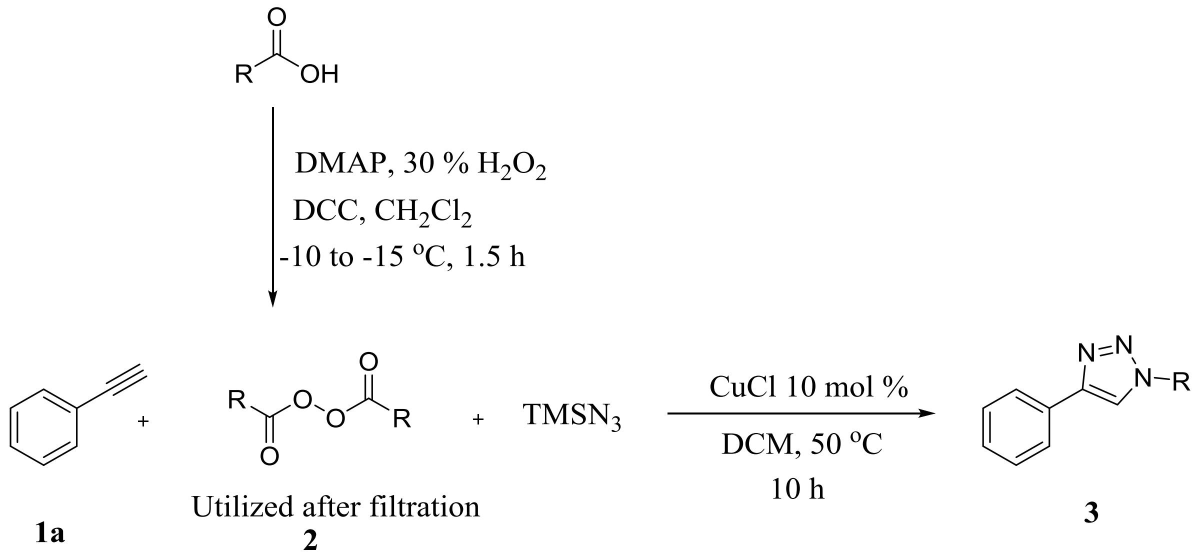

Utilized after filtration

1a **2** **3**

To an oven-dried Schlenk tube equipped with magnetic stirring bar, phenylacetylene (**1a**, 52.6 mg, 0.5 mmol), the above diacyl peroxide **2** (0.75 mmol), $TMSN_3$ (90.4 mg, 0.75 mmol), CuCl (4.9 mg, 0.05 mmol) and CH_2Cl_2 (2 mL) were added, respectively. The reaction mixture was stirred at 50 °C for 10 hours. Then, the reaction mixture was cooled to room temperature, poured into saturated sodium bicarbonate solution (25 mL) and extracted with CH_2Cl_2 (3 × 25 mL). After drying over $MgSO_4$, the solvent was removed under reduced pressure, and the residue was purified by column chromatography on silica gel (PE/EA) to afford **3**.

Characterization data for the products

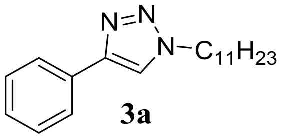

3a

Following the general procedure for Scheme 2, **3a** was obtained as a yellowish solid (145 mg, 97% yield); mp. 85 – 86 °C; ^{1}H NMR (400 MHz, $CDCl_3$) δ 7.87 – 7.77 (m, 2H), 7.74 (s, 1H), 7.39 (dd, *J* = 8.3, 6.8 Hz, 2H), 7.35 – 7.26 (m, 1H), 4.34 (t, *J* = 7.2 Hz, 2H), 1.90 (t, *J* = 7.1 Hz, 2H), 1.32 – 1.21 (m, 16H), 0.87 (t, *J* = 6.8 Hz, 3H); ^{13}C NMR (100 MHz, $CDCl_3$) δ 147.66, 130.79, 128.82, 128.04, 125.68, 119.53, 50.42, 31.91, 30.36, 29.58, 29.54, 29.41, 29.33, 29.04, 26.51, 22.70, 14.14; IR (KBr): υ 2551, 2917, 2847, 1652, 1558, 1464, 1216, 1078 cm^{-1}; HRMS (ESI) calcd for $[C_{19}H_{29}N_3Na]^+$ ($[M+Na]^+$): 322.2254, found: 322.2254.

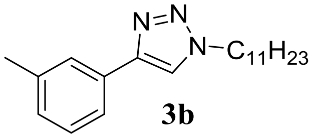

3b

Following the general procedure for Scheme 2, **3b** was obtained as a dull-white solid (130 mg, 83% yield); mp. 66 – 67 °C. ^{1}H NMR (400 MHz, $CDCl_3$) δ 7.72 (s, 1H), 7.69 (d, *J* = 2.0 Hz, 1H), 7.60 (d, *J* = 7.7 Hz, 1H), 7.31 (t, *J* = 7.6 Hz, 1H), 7.14 (d, *J* = 7.6 Hz, 1H), 4.38 (t, *J* = 7.2 Hz, 2H), 2.40 (s, 3H), 1.94 (t, *J* = 7.2 Hz, 2H), 1.34 – 1.25 (m, 16H), 0.87 (t, *J* = 6.8 Hz, 3H); ^{13}C NMR (100 MHz, $CDCl_3$) δ 147.96, 138.63, 130.74, 128.96, 128.84, 126.51, 122.91, 119.46, 50.56, 32.01, 30.49, 29.64, 29.51, 29.42, 29.15, 26.63, 22.80, 21.56, 14.24; IR (KBr): υ 2952, 2916, 2847, 1699, 1541, 1466, 1220, 1024 cm^{-1}; HRMS (ESI) calcd for $[C_{20}H_{31}N_3Na]^+$ ($[M+Na]^+$): 336.2410, found: 336.2409.

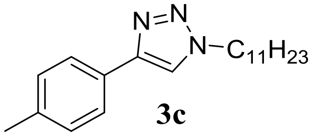

3c

Following the general procedure for Scheme 2, **3c** was obtained as a white solid (140 mg, 86% yield); mp. 86 –87 °C; ^{1}H NMR (400 MHz, $CDCl_3$) δ 7.78 – 7.65 (m, 3H), 7.21 (d, *J* = 7.8 Hz, 2H), 4.35 (t, *J* = 7.2 Hz, 2H), 2.37 (s, 3H), 1.91 (t, *J* = 7.1 Hz, 2H), 1.33 – 1.22 (m, 16H), 0.87 (t, *J* = 6.7 Hz, 3H); ^{13}C NMR (100 MHz, $CDCl_3$) δ 147.82, 137.92, 129.55, 128.01, 125.65, 119.18, 50.46, 31.96, 30.43, 29.63, 29.59, 29.46, 29.37, 29.09, 26.58, 22.74, 21.34, 14.18; IR (KBr): υ 2951, 2917, 2847, 1652, 1541, 1461, 1216, 1075 cm^{-1}; HRMS (ESI) calcd for $[C_{20}H_{31}N_3Na]^+$ ($[M+Na]^+$): 336.2410, found: 336.2412.

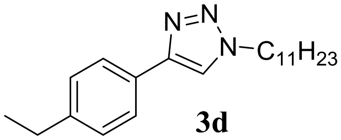

3d

Following the general procedure for Scheme 2, **3d** was obtained as a light brown solid (125 mg, 76% yield); mp. 69 – 70 °C; ^{1}H NMR (400 MHz, $CDCl_3$) δ 7.76 (d, *J* = 8.2 Hz, 2H), 7.71 (s, 1H), 7.28 (d, *J* = 2.5 Hz, 2H), 4.39 (t, *J* = 7.2 Hz, 2H), 2.69 (q, *J* = 7.6 Hz, 2H), 1.95 (t, *J* = 7.1 Hz, 2H), 1.35 – 1.25 (m, 19H), 0.91 – 0.87 (m, 3H); ^{13}C NMR (100 MHz, $CDCl_3$) δ 146.95, 143.44, 127.45, 127.28, 124.83, 118.19, 49.55, 31.02, 29.50, 28.69, 28.65, 28.52, 28.43, 28.16, 27.81, 25.64, 21.81, 14.66, 13.24; IR (KBr): υ 2953, 2917, 2847, 1699, 1541, 1457, 1217, 1076 cm^{-1}; HRMS (ESI) calcd for $[C_{21}H_{33}N_3Na]^+$ ($[M+Na]^+$): 350.2567, found: 350.2563.

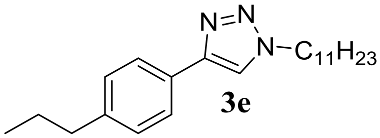

3e

Following the general procedure for Scheme 2, **3e** was obtained as a yellow solid (133 mg, 78% yield); mp. 61 –62 °C; ^{1}H NMR (400 MHz, $CDCl_3$) δ 7.74 (d, *J* = 8.2 Hz, 2H), 7.70 (s, 1H), 7.23 (d, *J* = 8.1 Hz, 2H), 4.38 (t, *J* = 7.2 Hz, 2H), 2.63 (t, , *J* = 7.4 Hz, 2H), 1.94 (t, *J* = 7.3 Hz, 2H), 1.69 – 1.63 (m, 2H), 1.33 – 1.25 (m, 16H), 0.95 (t, *J* = 7.3 Hz, 3H), 0.87 (t, *J* = 6.8 Hz, 3H); ^{13}C NMR (100 MHz, $CDCl_3$) δ 147.96, 142.88, 129.06, 128.26, 125.73, 119.19, 50.55, 37.95, 32.02, 30.49, 29.68, 29.65, 29.52, 29.43, 29.15, 26.64, 24.62, 22.80, 14.24, 13.92; IR (KBr): υ 2956, 2921, 2851, 1733, 1558, 1465, 1363, 1222 cm^{-1}; HRMS (ESI) calcd for $[C_{22}H_{35}N_3Na]^+$ ($[M+Na]^+$): 364.2723, found: 364.2723.

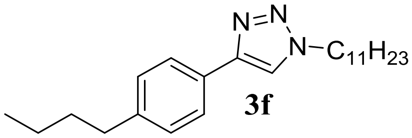

3f

Following the general procedure for Scheme 2, **3f** was obtained as a yellow solid (165 mg, 85% yield); mp. 73 –74 °C; ^{1}H NMR (400 MHz, $CDCl_3$) δ 7.79 – 7.65 (m, 3H), 7.23 (d, *J* = 8.1 Hz, 2H), 4.37 (t, *J* = 7.4 Hz, 2H), 2.63 (t, *J* = 7.4 Hz, 2H), 1.93 (t, *J* = 6.9 Hz, 2H), 1.62 (p, *J* = 7.5 Hz, 2H), 1.39 – 1.24 (m, 18H), 0.90 (m, 6H); ^{13}C NMR (100 MHz, $CDCl_3$) δ 147.91, 143.06, 128.97, 128.22, 125.70, 119.19, 50.50, 35.54, 33.67, 31.99, 30.46, 29.66, 29.62, 29.49, 29.41, 29.12, 26.60, 22.78, 22.44, 14.21, 14.06; IR (KBr): υ 2953, 2817, 2847, 1709, 1463, 1216, 1077 cm^{-1}; HRMS (EI) calcd for $[C_{23}H_{38}N_3][M+H]^+$: 356.3060, found: 356.3061.

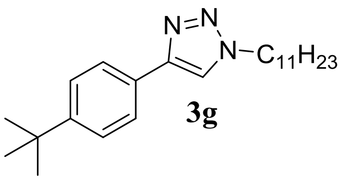

3g

Following the general procedure for Scheme 2, **3g** was obtained as a yellow solid (118 mg, 66% yield); mp. 47 –48 °C; ^{1}H NMR (400 MHz, $CDCl_3$) δ 7.76 (d, *J* = 8.4 Hz, 2H), 7.70 (s, 1H), 7.45 (d, *J* = 8.4 Hz, 2H), 4.38 (t, *J* = 7.2 Hz, 2H), 1.95 (t, *J* = 7.1 Hz, 2H), 1.36 (d, *J* = 36.4 Hz, 25H), 0.87 (t, *J* = 6.7 Hz, 3H); ^{13}C NMR (100 MHz, $CDCl_3$) δ 151.30, 147.84, 128.05, 125.87, 125.56, 119.23, 50.54, 34.79, 32.02, 31.43, 30.49, 29.69, 29.64, 29.52, 29.43, 29.15, 26.63, 22.81, 14.24; IR (KBr): υ 2953, 2917, 2847, 1684, 1541, 1457, 1217, 1076 cm^{-1}; HRMS (ESI) calcd for $[C_{23}H_{37}N_3Na]^+$ ($[M+Na]^+$): 378.2880, found: 378.2881.

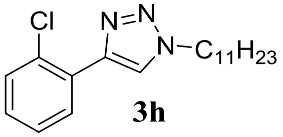

3h

Following the general procedure for Scheme 2, **3h** was obtained as a brown solid (130 mg, 78% yield); mp. 56 –57 °C; ^{1}H NMR (400 MHz, $CDCl_3$) δ 8.26 (dd, J = 7.8, 1.8 Hz, 1H), 8.16 (s, 1H), 7.45 (dd, *J* = 8.0, 1.3 Hz, 1H), 7.37 (td, *J* = 7.6, 1.4 Hz, 1H), 7.27 (dd, J = 7.7, 5.9 Hz, 1H), 4.42 (t, *J* = 7.3 Hz, 2H), 1.94 (t, *J* = 7.3 Hz, 2H), 1.35 – 1.26 (m, 16H), 0.88 (t, *J* = 6.8 Hz, 3H); ^{13}C NMR (100 MHz, $CDCl_3$) δ 144.04, 131.27, 130.30, 129.95, 129.53, 129.06, 127.31, 123.22, 50.62, 32.02, 30.48, 29.69, 29.64, 29.52, 29.44, 29.14, 26.63, 22.81, 14.25; IR (KBr): υ 2920, 2853, 1715, 1507, 1489, 1058, 759 cm^{-1}; HRMS (ESI) calcd for $[C_{19}H_{28}ClN_3Na]^+$ ($[M+Na]^+$): 356.1864, found: 356.1866.

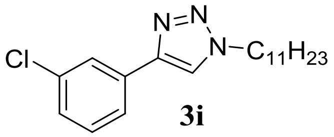

3i

Following the general procedure for Scheme 2, **3i** was obtained as a white solid (151 mg, 91% yield); mp. 79 – 80 °C; ^{1}H NMR (400 MHz, $CDCl_3$) δ 7.82 (d, *J* = 2.0 Hz, 1H), 7.75 (s, 1H), 7.72 (d, *J* = 7.6 Hz, 1H), 7.34 (t, *J* = 7.8 Hz, 1H), 7.29 (d, *J* = 8.0 Hz, 1H), 4.39 (t, *J* = 7.3 Hz, 2H), 1.94 (t, *J* = 7.1 Hz, 2H), 1.34 – 1.25 (m, 16H), 0.87 (t, *J* = 6.7 Hz, 3H); ^{13}C NMR (100 MHz, $CDCl_3$) δ 146.55, 134.89, 132.64, 130.23, 128.15, 125.86, 123.85, 119.89, 50.65, 32.00, 30.44, 29.66, 29.62, 29.49, 29.41, 29.11, 26.60, 22.79, 14.22; IR (KBr): υ 2917, 2847, 1670, 1575, 1489, 1070, 792 cm^{-1}; HRMS (ESI) calcd for $[C_{19}H_{28}ClN_3Na]^+$ ($[M+Na]^+$): 356.1864, found: 356.1863.

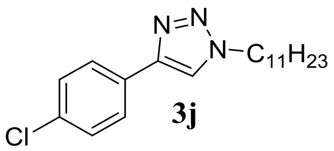

3j

Following the general procedure for Scheme 2, **3j** was obtained as a light yellow solid (153 mg, 92% yield); mp. 97 – 98 °C; ^{1}H NMR (400 MHz, CDCl$_3$) δ 7.76 (d, *J* = 8.5 Hz, 2H), 7.73 (s, 1H), 7.38 (d, *J* = 8.5 Hz, 2H), 4.38 (t, *J* = 7.2 Hz, 2H), 1.93 (t, *J* = 7.2 Hz, 2H), 1.34 – 1.24 (m, 16H), 0.87 (t, *J* = 6.7 Hz, 3H); ^{13}C NMR (100 MHz, CDCl$_3$) δ 146.79, 133.91, 129.39, 129.14, 127.04, 119.58, 50.63, 32.00, 30.46, 29.67, 29.63, 29.49, 29.41, 29.13, 26.62, 22.79, 14.23; IR (KBr): υ 2916, 2845, 1684, 1558, 1457, 1075, 827 cm^{-1}; HRMS (ESI) calcd for $[C_{19}H_{28}ClN_3Na]^+$ ($[M+Na]^+$): 356.1864, found: 356.1865.

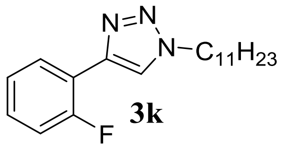

3k

Following the general procedure for Scheme 2, **3k** was obtained as a brown solid (121 mg, 76% yield); mp. 52 –53 °C; ^{1}H NMR (400 MHz, CDCl$_3$) δ 8.32 (td, *J* = 7.6, 2.0 Hz, 1H), 7.93 (d, *J* = 3.8 Hz, 1H), 7.33 – 7.22 (m, 2H), 7.14 (ddd, J = 11.2, 8.0, 1.5 Hz, 1H), 4.42 (t, *J* = 7.2 Hz, 2H), 1.92 (t, *J* = 7.2 Hz, 2H), 1.35 – 1.26 (m, 16H), 0.88 (t, *J* = 6.8 Hz, 3H); ^{13}C NMR (100 MHz, CDCl$_3$) δ 159.33 (d, *J* = 247.4 Hz), 141.20, 129.31 (d, *J* = 8.7 Hz), 127.89 (d, *J* = 3.6 Hz), 124.73 (d, *J* = 3.0 Hz), 122.70 (d, *J* = 12.6 Hz), 118.81 (d, *J* = 13.1 Hz), 115.74 (d, *J* = 21.9 Hz) 50.57, 32.00, 30.46, 29.68, 29.63, 29.50, 29.42, 29.12, 26.60, 22.80, 14.24; ^{19}F NMR (376 MHz, CDCl$_3$) δ -114.97 (s, 1F); IR (KBr): υ 2954, 2918, 2851, 1557, 1469, 1227, 1074 cm^{-1}; HRMS (ESI) calcd for $[C_{19}H_{28}FN_3Na]^+$ ($[M+Na]^+$): 340.2159, found: 340.2160.

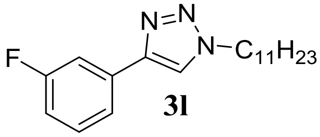

3l

Following the general procedure for Scheme 2, **3l** was obtained as a bright white solid (138 mg, 87% yield); mp. 73 – 74 °C; ^{1}H NMR (400 MHz, CDCl$_3$) δ 7.76 (s, 1H), 7.62 – 7.52 (m, 2H), 7.37 (td, *J* = 8.0, 5.9 Hz, 1H), 7.01 (td, *J* = 8.5, 2.6 Hz, 1H), 4.39 (t, *J* = 7.2 Hz, 2H), 1.94 (t, *J* = 7.1 Hz, 2H), 1.34 – 1.25 (m, 16H), 0.87 (t, *J* = 6.7 Hz, 3H); ^{13}C NMR (100 MHz, CDCl$_3$) δ 163.29 (d, *J* = 245.7 Hz), 146.75 (d, *J* = 2.9 Hz), 133.03 (d, *J* = 8.6 Hz), 130.50 (d, *J* = 8.2 Hz), 121.37 (d, *J* = 2.9 Hz), 119.91, 114.95 (d, *J* = 21.2 Hz), 112.70 (d, *J* = 23.0 Hz), 50.63, 31.99, 30.43, 29.66, 29.61, 29.48, 29.40, 29.11, 26.59, 22.77, 14.21; ^{19}F NMR (376 MHz, CDCl$_3$) δ -112.74 (s, 1F); IR (KBr): υ 2951, 2916, 2847, 1588, 1465, 1217, 1156 cm^{-1}; HRMS (ESI) calcd for $[C_{19}H_{28}FN_3Na]^+$ ($[M+Na]^+$): 340.2159, found: 340.2159.

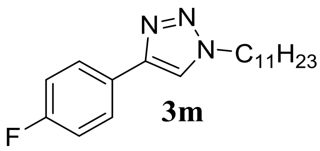

3m

Following the general procedure for Scheme 2, **3m** was obtained as a light yellow solid (141 mg, 89% yield); mp. 89.4 – 90.4 °C; ^{1}H NMR (400 MHz, $CDCl_3$) δ 7.86 – 7.73 (m, 2H), 7.70 (s, 1H), 7.11 (t, *J* = 8.5 Hz, 2H), 4.38 (t, *J* = 7.3 Hz, 2H), 1.94 (t, *J* = 7.1 Hz, 2H), 1.33 –1.25 (m, 16H), 0.87 (t, *J* = 6.7 Hz, 3H); ^{13}C NMR (100 MHz, $CDCl_3$) δ 162.76 (d, *J* = 247.2 Hz), 147.01, 127.54 (d, *J* = 8.1 Hz), 127.10 (d, *J* = 3.2 Hz), 119.26, 115.93 (d, *J* = 21.9 Hz), 50.62, 32.01, 30.48, 29.68, 29.64, 29.51, 29.43, 29.14, 26.63, 22.80, 14.24; ^{19}F NMR (376 MHz, $CDCl_3$) δ -113.72 (s, 1F); IR (KBr): υ 2917, 2846, 1716, 1636, 1521, 1454, 1339 cm^{-1}; HRMS (ESI) calcd for $[C_{19}H_{28}FN_3Na]^+$ ($[M+Na]^+$): 340.2159, found: 340.2156.

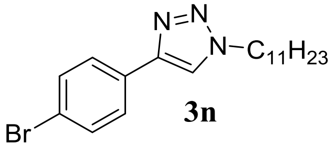

3n

Following the general procedure for Scheme 2, **3n** was obtained as a clear white solid (152 mg, 80% yield), mp. 87 – 88 °C; ^{1}H NMR (400 MHz, $CDCl_3$) δ 7.74 (s, 1H), 7.70 (d, *J* = 8.4 Hz, 2H), 7.54 (d, *J* = 8.3 Hz, 2H), 4.38 (t, *J* = 7.2 Hz, 2H), 1.90 (t, *J* = 7.2 Hz, 2H), 1.34 – 1.25 (m, 16H), 0.87 (t, *J* = 6.7 Hz, 3H); ^{13}C NMR (100 MHz, $CDCl_3$) δ 146.77, 132.06, 129.82, 127.31, 122.02, 119.61, 50.62, 31.98, 30.04, 29.65, 29.61, 29.48, 29.40, 29.10, 26.60, 22.77, 14.22; IR (KBr): υ 2917, 2847, 1684,1541, 1457, 1072, 825 cm^{-1}; HRMS (ESI) calcd for $[C_{19}H_{28}BrN_3Na]^+$ ($[M+Na]^+$): 400.1359, found: 400.1357.

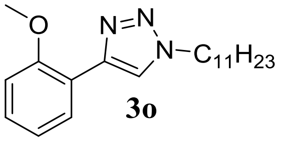

3o

Following the general procedure for Scheme 2, **3o** was obtained as a light yellow semi solid (130 mg, 79% yield); ^{1}H NMR (400 MHz, $CDCl_3$) δ 8.34 (dd, *J* = 7.7, 1.8 Hz, 1H), 8.01 (s, 1H), 7.31 (ddd, *J* = 8.3, 7.3, 1.8 Hz, 1H), 7.08 (td, J = 7.5, 1.1 Hz, 1H), 6.98 (dd, *J* = 8.3, 1.0 Hz, 1H), 4.39 (t, *J* = 7.3 Hz, 2H), 3.94 (s, 3H), 1.95 (t, *J* = 7.3 Hz 2H), 1.34 – 1.23 (m, 16H), 0.87 (t, *J* = 6.8 Hz, 3H); ^{13}C NMR (100 MHz, $CDCl_3$) δ 155.72, 143.17, 128.92, 127.81, 123.02, 121.21, 119.71, 110.90, 55.51, 50.39, 32.02, 30.55, 29.69, 29.66, 29.55, 29.44, 29.18, 26.67, 22.81, 14.24; IR (KBr): υ 2925, 2853, 1550, 1490, 1247, 1181, 1026 cm^{-1}; HRMS (ESI) calcd for $[C_{20}H_{31}N_3NaO]^+$ ($[M+Na]^+$): 352.2359, found: 352.2359.

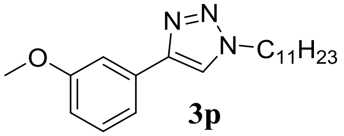

3p

Following the general procedure for Scheme 2, **3p** was obtained as a golden yellow solid (134 mg, 81% yield); mp. 44 – 45 °C; ^{1}H NMR (400 MHz, $CDCl_3$) δ 7.73 (s, 1H), 7.44 (dd, J = 2.6, 1.5 Hz, 1H), 7.36 – 7.27 (m, 2H), 6.86 (ddd, J = 7.9, 2.7, 1.3 Hz, 1H), 4.35 (t, J = 7.2 Hz, 2H), 3.84 (s, 3H), 1.91 (t, J = 7.2 Hz, 2H), 1.31 – 1.24 (m, 16H), 0.86 (t, J = 6.8 Hz, 3H); ^{13}C NMR (100 MHz, $CDCl_3$) δ 160.10, 147.61, 132.13, 129.90, 119.72, 118.13, 114.21, 110.26, 55.39. 50.49, 31.95, 30.40, 29.61, 29.58, 29.45, 29.36, 29.07, 26.55, 22.73, 14.17; IR (KBr): υ 2954, 2919, 2848, 1647, 1456, 1232, 1042 cm^{-1}; HRMS (ESI) calcd for $[C_{20}H_{31}N_3NaO]^+$ ($[M+Na]^+$): 352.2359, found: 352.2360.

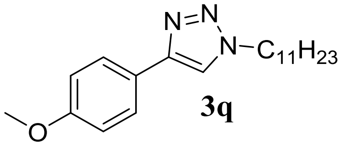

3q

Following the general procedure for Scheme 2, **3q** was obtained as a light yellow solid (126 mg, 77% yield); mp. 66.5 – 67.5 °C; ^{1}H NMR (400 MHz, $CDCl_3$) δ 7.82 – 7.71 (m, 2H), 7.65 (s, 1H), 7.01 – 6.90 (m, 2H), 4.37 (t, J = 7.2 Hz, 2H), 3.84 (s, 3H), 1.93 (t, J = 7.2 Hz, 2H), 1.33 – 1.23 (m, 16H), 0.87 (t, J = 6.8 Hz, 3H); ^{13}C NMR (100 MHz, $CDCl_3$) δ 159.67, 147.71, 127.11, 132.61, 118.72, 114.35, 55.45, 50.53, 32.01, 30.50, 29.68, 29.64, 29.51, 29.42, 29.15, 26.64, 22.80, 14.24; IR (KBr): υ 2952, 2917, 2846, 1647, 1541, 1457, 1021 cm^{-1}; HRMS (ESI) calcd for $[C_{20}H_{31}N_3NaO]^+$ ($[M+Na]^+$): 352.2359, found: 352.2363.

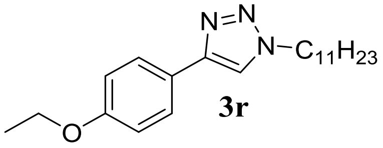

3r

Following the general procedure for Scheme 2, **3r** was obtained as a bright white solid (125 mg, 73% yield); mp. 102 – 103 °C; ^{1}H NMR (400 MHz, $CDCl_3$) δ 7.75 – 7.68 (m, 2H), 7.64 (s, 1H), 6.95 – 6.88 (m, 2H), 4.33 (t, J = 7.2 Hz, 2H), 4.04 (q, J= 7.0 Hz, 2H), 1.90 (t, J = 7.2 Hz, 2H), 1.40 (t, J = 7.0 Hz, 3H), 1.33 – 1.20 (m, 16H), 0.86 (t, J = 6.8 Hz, 3H); ^{13}C NMR (100 MHz, $CDCl_3$) δ 158.96, 147.64, 127.00, 123.40, 118.69, 114.81, 63.53, 50.41, 31.94, 30.41, 29.61, 29.58, 29.45, 29.36, 29.08, 26.56, 22.72, 14.87, 14.16; IR (KBr): υ 2975, 2917, 2846, 1652, 1465, 1250, 1114 cm^{-1}; HRMS (EI) calcd for $[C_{23}H_{34}N_3O][M+H]^+$: 344.2696, found: 344.2693.

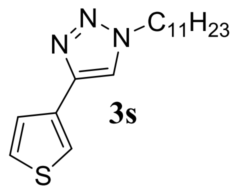

3s

Following the general procedure for Scheme 2, **3s** was obtained as a bright white solid (116 mg, 76% yield); mp. 94 – 95 °C; ^{1}H NMR (400 MHz, $CDCl_3$) δ 7.66 (dd, *J* = 2.9, 1.3 Hz, 1H), 7.64 (s, 1H), 7.45 (dd, *J* = 5.0, 1.3 Hz, 1H), 7.36 (dd, *J* = 5.0, 3.0 Hz, 1H), 4.36 (t, *J* = 7.2 Hz, 2H), 1.91 (t, *J* = 7.2 Hz, 2H), 1.33 – 1.22 (m, 16H), 0.87 (t, *J* = 6.8 Hz, 3H); ^{13}C NMR (100 MHz, $CDCl_3$) δ 143.96, 132.04, 126.36, 125.89, 120.99, 119.35, 50.47, 31.97, 30.43, 29.64, 29.60, 29.47, 29.39, 29.10, 26.56, 22.76, 14.21; IR (KBr): υ 2951, 2916, 2848, 1558, 1465, 1217, 783 cm^{-1}; HRMS (ESI) calcd for $[C_{17}H_{27}N_3NaS]^+$ ($[M+Na]^+$): 328.1818, found: 328.1818.

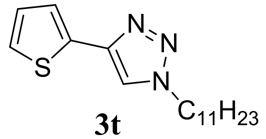

3t

Following the general procedure for Scheme 2, **3t** was obtained as an off-white solid (109 mg, 71% yield); mp. 67 – 68 °C; ^{1}H NMR (400 MHz, $CDCl_3$) δ 7.65 (s, 1H), 7.36 (dd, *J* = 3.6, 1.1 Hz, 1H), 7.27 (dd, *J* = 5.0, 1.1 Hz, 1H), 7.06 (dd, *J* = 5.1, 3.6 Hz, 1H), 4.35 (t, *J* = 7.3 Hz, 2H), 1.92 (t, *J* = 7.2 Hz, 2H), 1.32 – 1.24 (m, 16H), 0.87 (t, *J* = 6.7 Hz, 3H); ^{13}C NMR (100 MHz, $CDCl_3$) δ 142.86, 133.22, 127.67, 124.98, 124.08, 119.04, 50.57, 31.97, 30.40, 29.64, 29.60, 29.47, 29.39, 29.09, 26.56, 22.76, 14.20; IR (KBr): υ 2952, 2917, 2847, 1558, 1465, 1219, 686 cm^{-1}; HRMS (ESI) calcd for $[C_{17}H_{27}N_3NaS\ ]^+$ ($[M+Na]^+$): 328.1818, found: 328.1818.

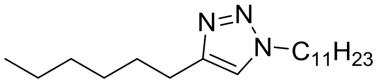

3u

Following the general procedure for Scheme 2, **3u** was obtained as a dull white solid (127.5 mg, 83% yield); mp. = 56–57 °C; ^{1}H NMR (600 MHz, $CDCl_3$) δ 7.22 (s, 1H), 4.31 – 4.20 (m, 2H), 2.65 (t, *J* = 7.5 Hz, 2H), 1.82 (t, *J* = 7.8 Hz, 2H), 1.61 (p, *J* = 8.0 Hz, 2H), 1.30 – 1.17 (m, 22H), 0.85 – 0.80 (m, 6H); ^{13}C NMR (150 MHz, $CDCl_3$) δ 148.38, 120.40, 50.17, 31.91, 31.62, 30.39, 29.58, 29.54, 29.50, 29.42, 29.33, 29.04, 28.96, 26.53, 25.74, 22.70, 22.59, 14.12, 14.08; IR (KBr): υ 2953, 2916, 2848, 1636, 1468, 1212, 1154, 1061 cm^{-1}; HRMS (ESI) calcd for $[C_{19}H_{37}N_3Na]^+$ ($[M+Na]^+$): 330.2880, found: 330.2880.

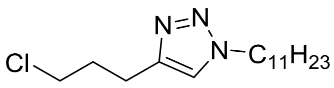

3v

Following the general procedure for Scheme 2, **3v** was obtained as an off-White solid (102 mg, 68% yield); mp. = 48–49 °C. ^{1}H NMR (600 MHz, $CDCl_3$) δ 7.30 (s, 1H), 4.54 – 3.92 (m, 2H), 3.55 (t, *J* = 6.4 Hz, 2H), 3.04 – 2.70 (m, 2H), 2.30 – 2.01 (m, 2H), 1.91 – 1.75 (m, 2H), 1.46 – 1.15 (m, 16H), 0.85 (t, *J* = 7.1 Hz, 3H); ^{13}C NMR (150 MHz, $CDCl_3$) δ 146.30, 121.02, 50.31, 44.29, 31.96, 31.95, 30.40, 29.62, 29.57, 29.45, 29.37, 29.06, 26.57, 22.74, 22.72, 14.19; IR (KBr): υ 2952, 2916, 2847, 1646, 1489, 1305, 1212 842 cm^{-1};HRMS (ESI) calcd for $[C_{16}H_{30}ClN_3Na]^+$ ($[M+Na]^+$): 322.2020, found: 322.2020.

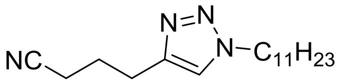

3w

Following the general procedure for Scheme 2, **3w** was obtained as an off-White solid (118 mg, 81% yield), mp. = 60–61 °C; ^{1}H NMR (600 MHz, $CDCl_3$) δ 7.31 (s, 1H), 4.27 (t, *J* = 7.3 Hz, 2H), 2.82 (t, *J* = 7.5 Hz, 2H), 2.38 (t, *J* = 7.1 Hz, 2H), 2.03 (t, *J* = 7.2 Hz, 2H), 1.84 (t, *J* = 7.9 Hz, 2H), 1.28 – 1.17 (m, 16H), 0.82 (t, *J* = 8.5 Hz 3H); ^{13}C NMR (150 MHz, $CDCl_3$) δ 145.44, 121.14, 119.42, 50.29, 31.87, 30.31, 29.54, 29.50, 29.37, 29.29, 28.98, 26.49, 24.92, 24.20, 22.66, 16.47, 14.11; IR (KBr): υ 2953, 2919, 2847, 2246, 1636, 1463, 1372, 1210 cm^{-1}; HRMS (ESI) calcd for $[C_{17}H_{30}N_4Na]^+$ ($[M+Na]^+$): 313.2363, found: 313.2363.

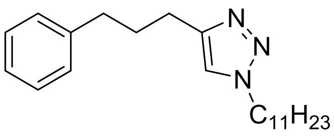

3x

Following the general procedure for Scheme 2, **3x** was obtained as a yellowish solid (133 mg, 78% yield); mp. = 40–41 °C; ^{1}H NMR (600 MHz, $CDCl_3$) δ 7.30 – 7.25 (m, 3H), 7.23 – 7.17 (m, 3H), 4.30 (td, *J* = 7.5, 1.4 Hz, 2H), 2.76 (t, *J* = 7.9 Hz, 2H), 2.69 (t, *J* = 8.1 Hz, 2H), 2.05 –2.00 (m, 2H), 1.88 (t, *J* = 7.2 Hz, 2H), 1.37 – 1.21 (m, 16H), 0.89 (t, *J* = 7.6 Hz, 3H); ^{13}C NMR (150 MHz, $CDCl_3$) δ 141.93, 128.48, 128.33, 125.82, 120.51, 50.17, 35.40, 31.89, 31.13, 30.35, 29.56, 29.52, 29.40, 29.31, 29.02, 26.51, 25.22, 22.68, 14.13; IR (KBr): υ 2953, 2917, 2847, 1646, 1461, 1211, 1146, 1056 cm^{-1}; HRMS (ESI) calcd for $[C_{22}H_{35}N_3Na]^+$ ($[M+Na]^+$): 364.2723, found: 364.2723.

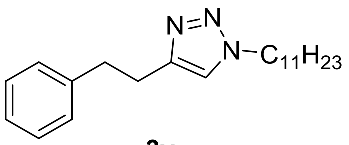

3y

Following the general procedure for Scheme 2, **3y** was obtained as an off-White solid (136 mg, 83% yield); mp. = 55–56 °C; ^{1}H NMR (600 MHz, $CDCl_3$) δ 7.24 (t, *J* = 7.4 Hz, 2H), 7.16 (dd, *J* = 13.0, 7.7 Hz, 3H), 7.06 (s, 1H), 4.23 (t, *J* = 7.2 Hz, 2H), 3.09 – 2.88 (m, 2H), 1.81– 1.79 (m, 2H), 1.31 – 1.08 (m, 16H), 0.86 (t, *J* = 7.1 Hz, 3H); ^{13}C NMR (150 MHz, $CDCl_3$) δ 147.14, 141.26, 128.53, 128.38, 126.09, 120.82, 50.17, 35.66, 31.92, 30.36, 29.60, 29.56, 29.42, 29.34, 29.04, 27.57, 26.47, 22.71, 14.16; IR (KBr): υ 2952, 2917, 2847, 1636, 1463, 1211, 1152, 1059 cm^{-1}; HRMS (ESI) calcd for $[C_{21}H_{33}N_3Na]^+$ ($[M+Na]^+$): 350.2567, found: 350.2567.

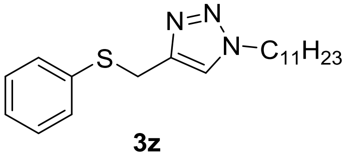

3z

Following the general procedure for Scheme 2, **3z** was obtained as an off-White solid (138 mg, 80% yield); mp. = 76–77 °C; ^{1}H NMR (600 MHz, $CDCl_3$) δ 7.31 (d, *J* = 8.3 Hz, 2H), 7.28 (s, 1H), 7.24 (t, *J* = 7.7 Hz, 2H), 7.16 (t, *J* = 8.2 Hz, 1H), 4.27 – 4.19 (m, 4H), 1.80 (t, *J* = 7.4 Hz, 2H), 1.29 – 1.19 (m, 16H), 0.86 (t, *J* = 7.74 Hz, 3H); ^{13}C NMR (150 MHz, $CDCl_3$) δ 144.86, 135.62, 129.55, 129.00, 126.47, 121.90, 50.39, 31.94, 30.28, 29.61, 29.56, 29.42, 29.35, 29.02, 28.93, 26.44, 22.73, 14.18; IR (KBr): υ 2952, 2917, 2847, 1635, 1464, 1437, 1240, 1060 cm^{-1}; HRMS (ESI) calcd for $[C_{20}H_{31}N_3SNa]^+$ ($[M+Na]^+$): 368.2131, found: 368.2131.

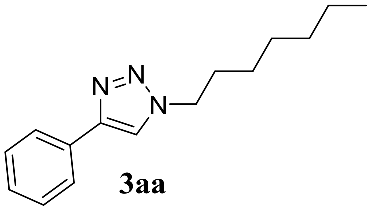

3aa

Following the general procedure for Scheme 3, **3aa** was obtained as a white solid (89 mg, 73% yield); mp. 63 –64 °C; ^{1}H NMR (400 MHz, $CDCl_3$) δ 7.83 (dd, *J* = 7.2, 1.7 Hz, 2H), 7.74 (s, 1H), 7.42 (t, *J* = 7.6 Hz, 2H), 7.36 – 7.27 (m, 1H), 4.38 (t, *J* = 7.3 Hz, 2H), 1.94 (dd, *J* = 8.8, 5.7 Hz, 2H), 1.34 – 1.26 (m, 8H), 0.87 (t, *J* = 7.1 Hz, 3H); ^{13}C NMR (100 MHz, $CDCl_3$) δ 147.82, 130.86, 128.93, 128.18, 125.80, 119.51, 50.55, 31.68, 30.47, 28.79, 26.57, 22.63, 14.13; IR (KBr): υ 2952, 2925, 2852, 1616, 1464, 1216, 1078 cm^{-1}; HRMS (ESI) calcd for $[C_{15}H_{21}N_3Na]^+$ ($[M+Na]^+$): 266.1628, found: 266.1629.

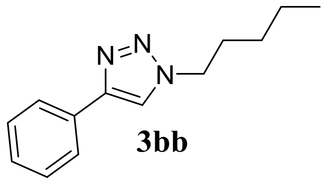

3bb

Following the general procedure for Scheme 3, **3bb** was obtained as a yellowish solid (78 mg, 72% yield); mp. 64 – 65 °C; ^{1}H NMR (400 MHz, $CDCl_3$) δ 7.87 – 7.80 (m, 2H), 7.74 (s, 1H), 7.46 – 7.39 (m, 2H), 7.35 – 7.29 (m, 1H), 4.39 (t, *J* = 7.3 Hz, 2H), 1.99 – 1.91 (m, 2H), 1.39 – 1.30 (m, 4H), 0.91 (t, *J* = 6.9 Hz, 3H); ^{13}C NMR (100 MHz, $CDCl_3$) δ 147.86, 130.86, 128.96, 128.21, 125.82, 119.51, 50.56, 30.18, 28.74, 22.24, 13.98; IR (KBr): υ 2951, 2922, 2855, 1646, 1464, 1216, 1077 cm^{-1}; HRMS (ESI) calcd for $[C_{13}H_{17}N_3Na]^+$ ($[M+Na]^+$): 238.1315, found: 238.1318.

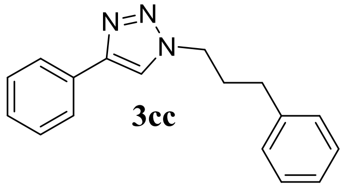

3cc

Following the general procedure for Scheme 3, **3cc** was obtained as a light yellow solid (118 mg, 90% yield); mp. 70 – 71 °C; ^{1}H NMR (400 MHz, $CDCl_3$) δ 7.87 – 7.80 (m, 2H), 7.72 (s, 1H), 7.48 – 7.39 (m, 2H), 7.39 – 7.27 (m, 3H), 7.25 – 7.15 (m, 3H), 4.40 (t, *J* = 7.1 Hz, 2H), 2.69 (t, *J* = 7.5 Hz, 2H), 2.36 – 2.24 (m, 2H); ^{13}C NMR (100 MHz, $CDCl_3$) δ 147.87, 140.25, 130.77, 128.95, 128.74, 128.57, 128.24, 126.49, 125.80, 119.65, 49.68, 32.62, 31.78; IR (KBr): υ 3027, 2922, 2851, 1684, 1456, 1224, 1077 cm^{-1}; HRMS (ESI) calcd for $[C_{17}H_{17}N_3Na]^+$ ($[M+Na]^+$): 286.1315, found: 286.1307.

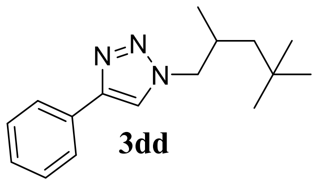

3dd

Following the general procedure for Scheme 3, **3dd** was obtained as a bright white solid (89 mg, 69% yield); mp. 99 – 100 °C; ^{1}H NMR (400 MHz, $CDCl_3$) δ 7.84 (dd, *J* = 8.3, 1.3 Hz, 2H), 7.73 (s, 1H), 7.45 – 7.39 (m, 2H), 7.35 – 7.30 (m, 1H), 4.27 (dd, *J* = 13.5, 6.6 Hz, 1H), 4.12 (dd, *J* = 13.5, 8.0 Hz, 1H), 2.21 – 2.11 (m, 1H), 1.32 (dd, *J* = 14.1, 3.5 Hz, 1H), 1.16 (dd, *J* = 14.1, 6.5 Hz, 1H), 0.97 (d, *J* = 6.7 Hz, 3H), 0.89 (s, 9H); ^{13}C

NMR (100 MHz, $CDCl_3$) δ 147.71, 130.86, 128.95, 128.20, 125.83, 120.02, 57.84, 47.87, 31.28, 31.08, 29.89, 20.45; IR (KBr): υ 3087, 2955, 1647, 1464, 1226, 1082 cm^{-1}; HRMS (ESI) calcd for $[C_{16}H_{23}N_3Na]^+$ ($[M+Na]^+$): 280.1784, found: 280.1784.

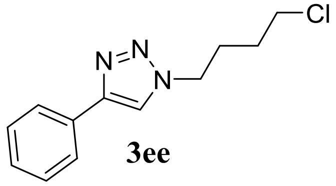

3ee

Following the general procedure for Scheme 3, **3ee** was obtained as a white solid (86 mg, 73% yield); mp. 51 – 52 °C; ^{1}H NMR (400 MHz, $CDCl_3$) δ 7.83 – 7.79 (m, 2H), 7.77 (s, 1H), 7.40 (td, *J* = 8.5, 7.8, 2.0 Hz, 2H), 7.34 – 7.29 (m, 1H), 4.41 (t, *J* = 7.0 Hz, 2H), 3.54 (t, *J* = 6.3 Hz, 2H), 2.10 (dt, *J* = 14.8, 7.0 Hz, 2H), 1.83 – 1.76 (m, 2H); ^{13}C NMR (100 MHz, $CDCl_3$) δ 147.90, 130.58, 128.90, 128.22, 125.71, 119.61, 49.55, 44.08, 29.22, 27.59; IR (KBr): υ 2956, 2922, 2850, 1652, 1223, 1077, 746 cm^{-1}; HRMS (ESI) calcd for $[C_{12}H_{14}ClN_3Na]^+$ ($[M+Na]^+$): 258.0768, found: 258.0769.

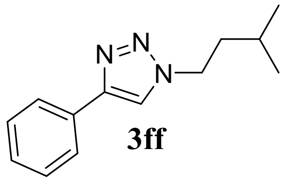

3ff

Following the general procedure for Scheme 3, **3ff** was obtained as an off-white solid (84 mg, 78% yield); mp. 77.7 – 78.7 °C; ^{1}H NMR (400 MHz, $CDCl_3$) δ 7.85 – 7.81 (m, 2H), 7.75 (s, 1H), 7.41 (t, *J* = 7.5 Hz, 2H), 7.34 – 7.30 (m, 1H), 4.43 – 4.38 (m, 2H), 1.84 (dt, *J* = 8.6, 7.1 Hz, 2H), 1.62 (dt, *J* = 13.4, 6.7 Hz, 1H), 0.98 (d, *J* = 6.7 Hz, 6H); ^{13}C NMR (100 MHz, $CDCl_3$) δ 147.83, 130.83, 128.91, 128.15, 125.77, 119.45, 48.84, 39.17, 25.61, 22.30; IR (KBr): υ 3082, 2956, 1733, 1558, 1447, 1222, 1078 cm^{-1}; HRMS (ESI) calcd for $[C_{13}H_{17}N_3Na]^+$ ($[M+Na]^+$): 238.1315, found: 238.1316.

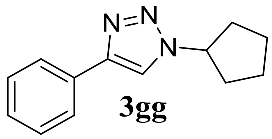

3gg

Following the general procedure for Scheme 3, **3gg** was obtained as a yellow solid (93 mg, 87% yield); mp. 68 –69 °C; ^{1}H NMR (400 MHz, $CDCl_3$) δ 7.83 – 7.80 (m, 2H), 7.75 (s, 1H), 7.42 – 7.38 (m, 2H), 7.33 – 7.28 (m, 1H), 4.95 (tt, *J* = 7.6, 6.2 Hz, 1H), 2.31 – 2.23 (m, 2H), 2.12 – 2.04 (m, 2H), 1.94– 1.88 (m, 2H), 1.82 – 1.73

(m, 2H); ^{13}C NMR (100 MHz, $CDCl_3$) δ 147.53, 130.87, 128.80, 128.00, 125.66, 118.09, 61.91, 33.44, 24.11; IR (KBr): υ 3096, 2955, 2872, 1652, 1455, 1224, 1080 cm^{-1}; HRMS (ESI) calcd for $[C_{13}H_{15}N_3Na]^+$ ($[M+Na]^+$): 236.1158, found: 236.1158.

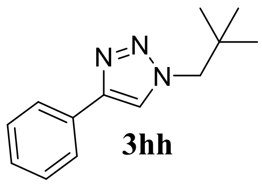

3hh

Following the general procedure for Scheme 3, **3hh** was obtained as a white solid (83 mg, 77% yield); mp. 108 –109 °C; ^1H NMR (400 MHz, $CDCl_3$) δ 7.88 – 7.81 (m, 2H), 7.71 (s, 1H), 7.46 – 7.39 (m, 2H), 7.33 (d, J = 7.4 Hz, 1H), 4.18 (s, 2H), 1.02 (s, 9H); ^{13}C NMR (100 MHz, $CDCl_3$) δ 147.31, 130.81, 128.93, 128.16, 125.80, 120.87, 61.94, 32.76, 27.62; IR (KBr): υ 2933, 2856, 1655, 1541, 1463, 1226, 1079 cm^{-1}; HRMS (ESI) calcd for $[C_{13}H_{17}N_3Na]^+$ ($[M+Na]^+$): 238.1315, found: 238.1316.

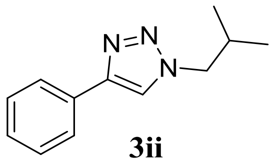

3ii

Following the general procedure for Scheme 3, **3ii** was obtained as a light yellow solid (93 mg, 92% yield); mp. 62 – 63 °C; ^1H NMR (400 MHz, $CDCl_3$) δ 7.88 – 7.81 (m, 2H), 7.73 (s, 1H), 7.45 – 7.39 (m, 2H), 7.35 – 7.29 (m, 1H), 4.20 (d, J = 7.2 Hz, 2H), 2.26 (dt, J = 13.7, 6.9 Hz, 1H), 0.97 (d, J = 6.7 Hz, 6H); ^{13}C NMR (100 MHz, $CDCl_3$) δ 147.68, 130.81, 128.92, 128.17, 125.77, 120.00, 57,73, 29.84, 19.95; IR (KBr): υ 2960, 2928, 2872, 1652, 1465, 1224, 1070 cm^{-1}; HRMS (ESI) calcd for $[C_{12}H_{15}N_3Na]^+$ ($[M+Na]^+$): 224.1158, found: 224.1160.

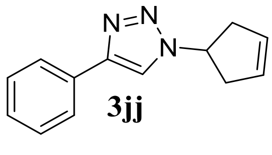

3jj

Following the general procedure for Scheme 3, **3jj** was obtained as a brown semi solid (26 mg, 60% yield); ^1H NMR (400 MHz, $CDCl_3$) δ 7.84 – 7.80 (m, 2H), 7.74 (s, 1H), 7.43 – 7.38 (m, 2H), 7.34 – 7.29 (m, 1H), 5.88 (s, 2H), 5.42 (tt, J = 8.3, 3.6 Hz, 1H), 3.12 – 3.04 (m, 2H), 2.74 (dd, J = 15.7, 3.4 Hz, 2H); ^{13}C NMR (100 MHz, $CDCl_3$) δ 148.18, 130.86, 128.91, 128.87, 128.18, 125.78, 117.17, 59.32, 40.77; IR (KBr): υ 3061, 2929,

2854, 1653, 1451, 1226, 1077 cm^{-1}; HRMS (ESI) calcd for $[C_{13}H_{13}N_3Na]^+$ ($[M+Na]^+$): 234.1002, found: 234.1001.

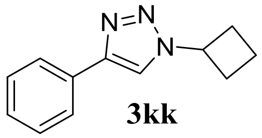

3kk

Following the general procedure for Scheme 3, **3kk** was obtained as a light white solid (95 mg, 95% yield); mp. 74 – 75 °C; ^{1}H NMR (400 MHz, $CDCl_3$) δ 7.84 – 7.79 (m, 3H), 7.39 (dd, J = 8.5, 6.8 Hz, 2H), 7.32 – 7.27 (m, 1H), 5.09 – 4.98 (m, 1H), 2.63 – 2.53 (m, 4H), 1.98 – 1.85 (m, 2H); ^{13}C NMR (100 MHz, $CDCl_3$) δ 147.53, 130.80, 128.80, 128.02, 125.68, 118.12, 54.11, 30.76, 15.02; IR (KBr): υ 2996, 2871, 1647, 1569, 1456, 1220, 1072 cm^{-1}; HRMS (ESI) calcd for $[C_{12}H_{13}N_3Na]^+$ ($[M+Na]^+$): 222.1002, found: 222.1001.

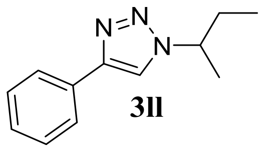

3ll

Following the general procedure for Scheme 3, **3ll** was obtained as a bright white solid (89 mg, 94% yield); mp. 54 – 55 °C; ^{1}H NMR (400 MHz, $CDCl_3$) δ 7.85 – 7.81 (m, 2H), 7.75 (s, 1H), 7.43 – 7.37 (m, 2H), 7.33 – 7.28 (m, 1H), 4.66 – 4.56 (m, 1H), 2.00 – 1.85 (m, 2H), 1.58 (d, J = 6.8 Hz, 3H), 0.87 (t, J = 7.4 Hz, 3H); ^{13}C NMR (100 MHz, $CDCl_3$) δ 147.54, 130.93, 128.86, 128.06, 125.70, 117.53, 59.01, 30.38, 21.04, 10.51; IR (KBr): υ 2930, 2854, 1653, 1533, 1452, 1226, 1077 cm^{-1}; HRMS (ESI) calcd for $[C_{12}H_{15}N_3Na]^+$ ($[M+Na]^+$): 224.1158, found: 224.1159.

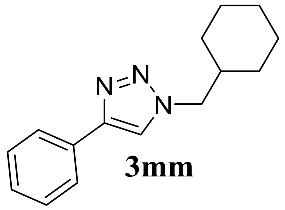

3mm

Following the general procedure for Scheme 3, **3mm** was obtained as a pale yellow solid (115 mg, 95% yield); mp. 95 – 96 °C; ^{1}H NMR (400 MHz, $CDCl_3$) δ 7.86 – 7.79 (m, 2H), 7.71 (s, 1H), 7.44 – 7.36 (m, 2H), 7.34 – 7.28 (m, 1H), 4.20 (d, J = 7.2 Hz, 2H), 1.91 – 1.88 (m, 1H), 1.74 – 1.63 (m, 4H), 1.33 – 1.08 (m, 4H), 1.06 – 0.95 (m, 2H); ^{13}C NMR (100 MHz, $CDCl_3$) δ 147.60, 130.81, 128.89, 128.12, 125.74, 120.14, 56.62, 38.88, 30.57, 26.14, 25.59; IR (KBr): υ 2922, 2849, 1507, 1457, 1221, 1085 cm^{-1}; HRMS (ESI) calcd for $[C_{15}H_{19}N_3Na]^+$ ($[M+Na]^+$): 264.1471, found: 264.1471.

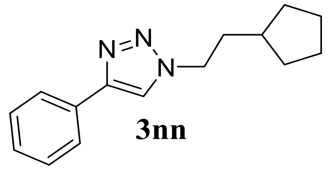

3nn

Following the general procedure for Scheme 3, **3nn** was obtained as a dull white solid (78 mg, 64% yield); mp. 98 – 99 °C; ^{1}H NMR (400 MHz, $CDCl_3$) δ 7.88 – 7.79 (m, 2H), 7.74 (s, 1H), 7.41 (dd, *J* = 8.3, 6.8 Hz, 2H), 7.35 – 7.29 (m, 1H), 4.39 (t, *J* = 7.3, Hz, 2H), 1.96 (dt, *J* = 8.6, 7.0 Hz, 2H), 1.87 – 1.47 (m, 8H), 1.15 (ddt, *J* = 8.4, 4.9, 2.0 Hz, 1H); ^{13}C NMR (100 MHz, $CDCl_3$) δ 147.81, 130.84, 128.92, 128.17, 125.79, 119.48, 49.97, 37.30, 36.69, 32.48, 25.16; IR (KBr): υ 2949, 2871, 1647, 1457, 1220, 1086 cm^{-1}; HRMS (ESI) calcd for $[C_{15}H_{19}N_3Na]^+$ ($[M+Na]^+$): 264.1471, found: 264.1472.

Preliminary mechanistic studies

(a) With TEMPO

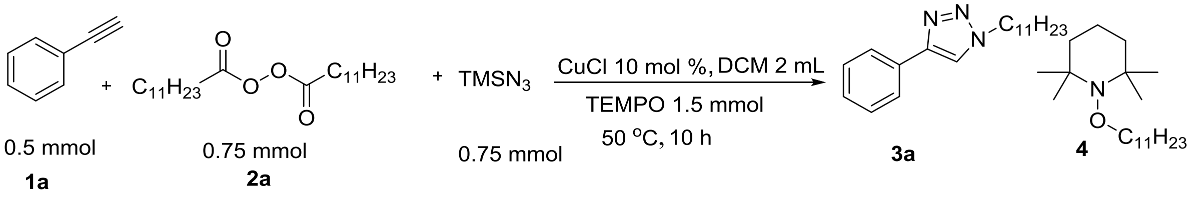

Phenyl acetylene (**1a**, 52.6 mg, 0.5 mmol), LPO (398.96 mg, 0.75 mmol), CuCl (4.9 mg, 0.05 mmol), TEMPO (1.5 mmol), $TMSN_3$ (90.4 mg, 0.75 mmol), and CH_2Cl_2 (2 mL) were added into a flame-dried Schlenk tube with a stirring bar and stirred at 50 °C for 10 hours. Then, the reaction mixture was cooled to ambient temperature, poured into saturated sodium bicarbonate solution (25 mL) and extracted with CH_2Cl_2 (3 × 25 mL) and passed through a short pad of celite. The filtrate was analyzed by GC–MS. No product (**3a**) was observed, only compound **4** was detected by GC–MS.

(b) Ring opening experiment

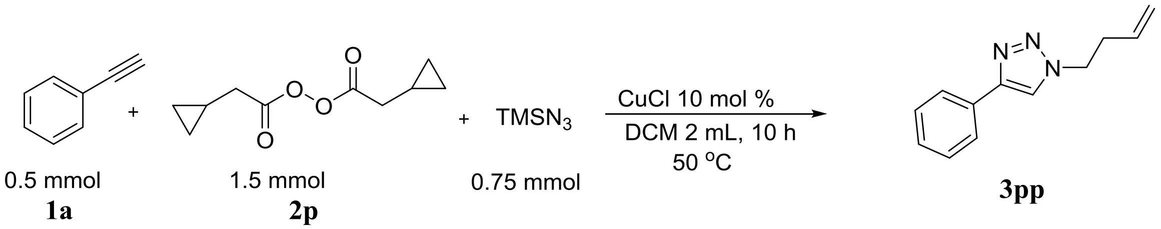

CuCl (4.9 mg, 10 mol %), $TMSN_3$ (90.4 mg, 0.75 mmol), phenylacetylene (**1a,** 52.6 mg, 0.5 mmol), diacyl peroxide **2p** (1.5 mmol) and CH_2Cl_2 (2 mL) were added to a flame-dried Schlenk tube with a stirring bar. The reaction mixture was heated to 50 °C for 10 hours, then cooled to room temperature. The solvent was removed by rotary evaporation, and the residue was chromatographed on silica gel which afforded the ring-opening product **3pp** in 88% yield.

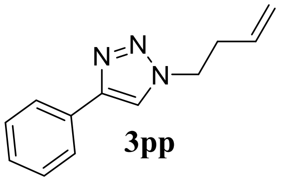

3pp

Compound **3pp** was obtained as a light brown solid (94 mg, 88% yield); mp. 53 – 54 °C; ^{1}H NMR (400 MHz, $CDCl_3$) δ 7.84 – 7.79 (m, 2H), 7.75 (s, 1H), 7.43 – 7.37 (m, 2H), 7.34 – 7.29 (m, 1H), 5.87 – 5.68 (m, 1H), 5.12 (dt, *J* = 2.3, 1.4 Hz, 1H), 5.10 – 5.03 (m, 1H), 4.44 (t, *J* = 7.1 Hz, 2H), 2.71 – 2.65 (m, 2H); ^{13}C NMR (100 MHz, $CDCl_3$) δ 147.71, 133.23, 130.75, 128.90, 128.16, 125.75, 119.74, 118.58, 49.80, 34.51; IR (KBr): υ 3096, 2927, 2854, 1646, 1450, 1225, 1080 cm^{-1}; HRMS (ESI) calcd for $[C_{12}H_{13}N_3Na]^+$ ($[M+Na]^+$): 222.1002, found: 222.1000.

References

1. Li, Y.; Han, Y.; Xiong, H.; Zhu, N.; Qian, B.; Ye, C.; Kantchev, E. A. B.; Bao, H. *Org. Lett.*, **2016**, *18*, 392.
2. Sheldon, R. A.; Kochi, J. K. *J. Am. Chem. Soc.*, **1970**, *92*, 4395.

Copies of ^{1}H, ^{13}C, and ^{19}F NMR spectra

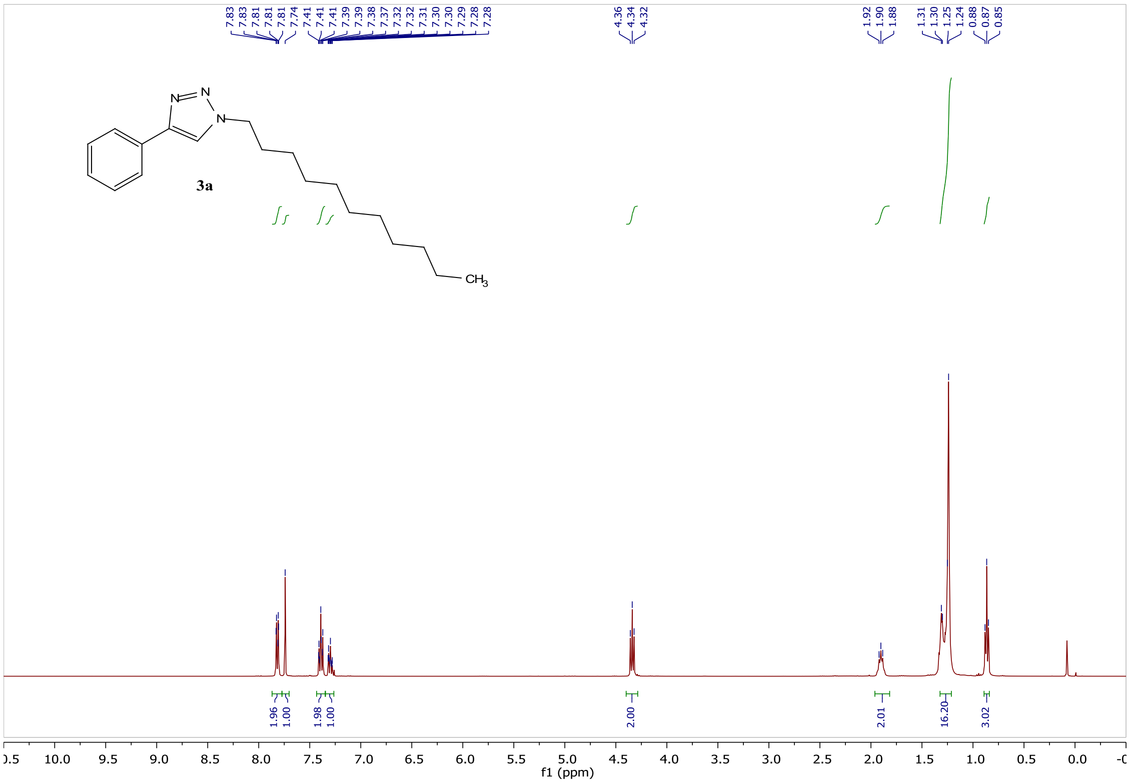

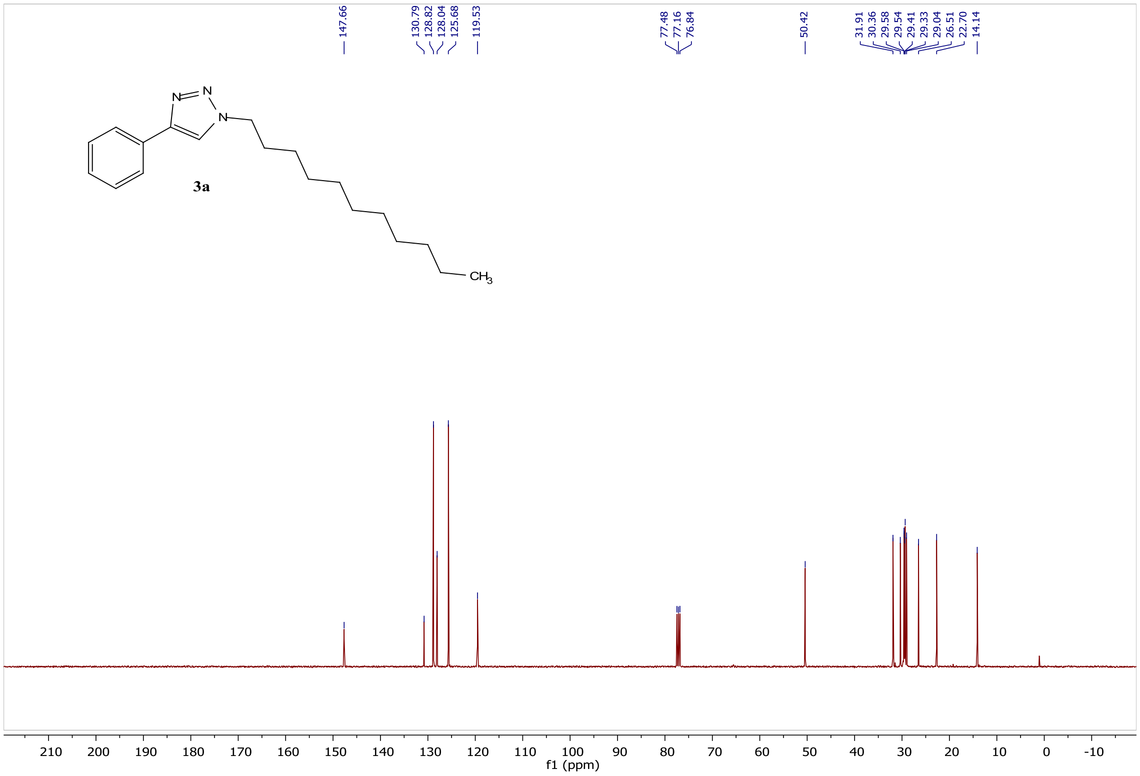

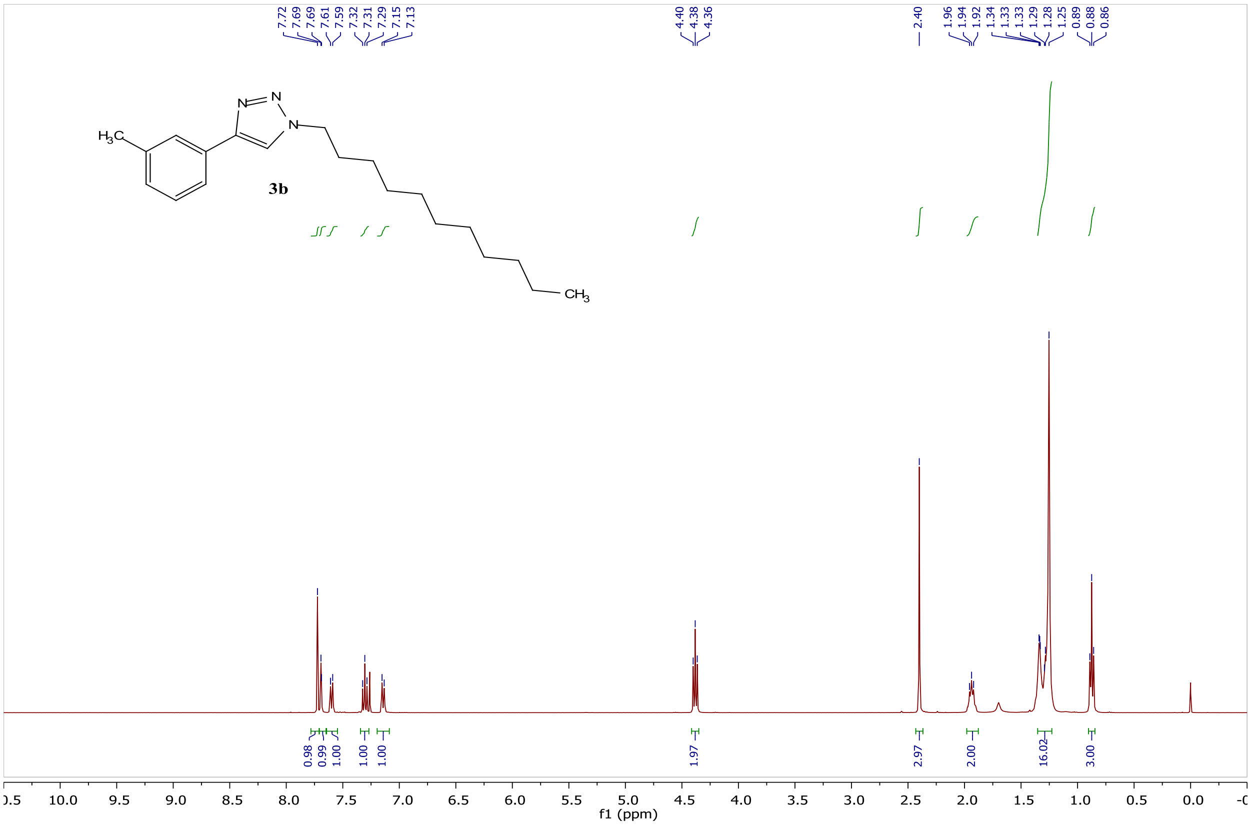
7.72
7.69
7.69
7.61
7.59
7.32
7.31
7.29
7.15
7.13
4.40
4.38
4.36
2.40
1.96
1.94
1.92
1.34
1.33
1.33
1.29
1.28
1.25
0.89
0.88
0.86
H_3C
N
N
N
3b
CH_3
0.98
0.99
1.00
1.00
1.00
1.97
2.97
2.00
16.02
3.00
f1 (ppm)

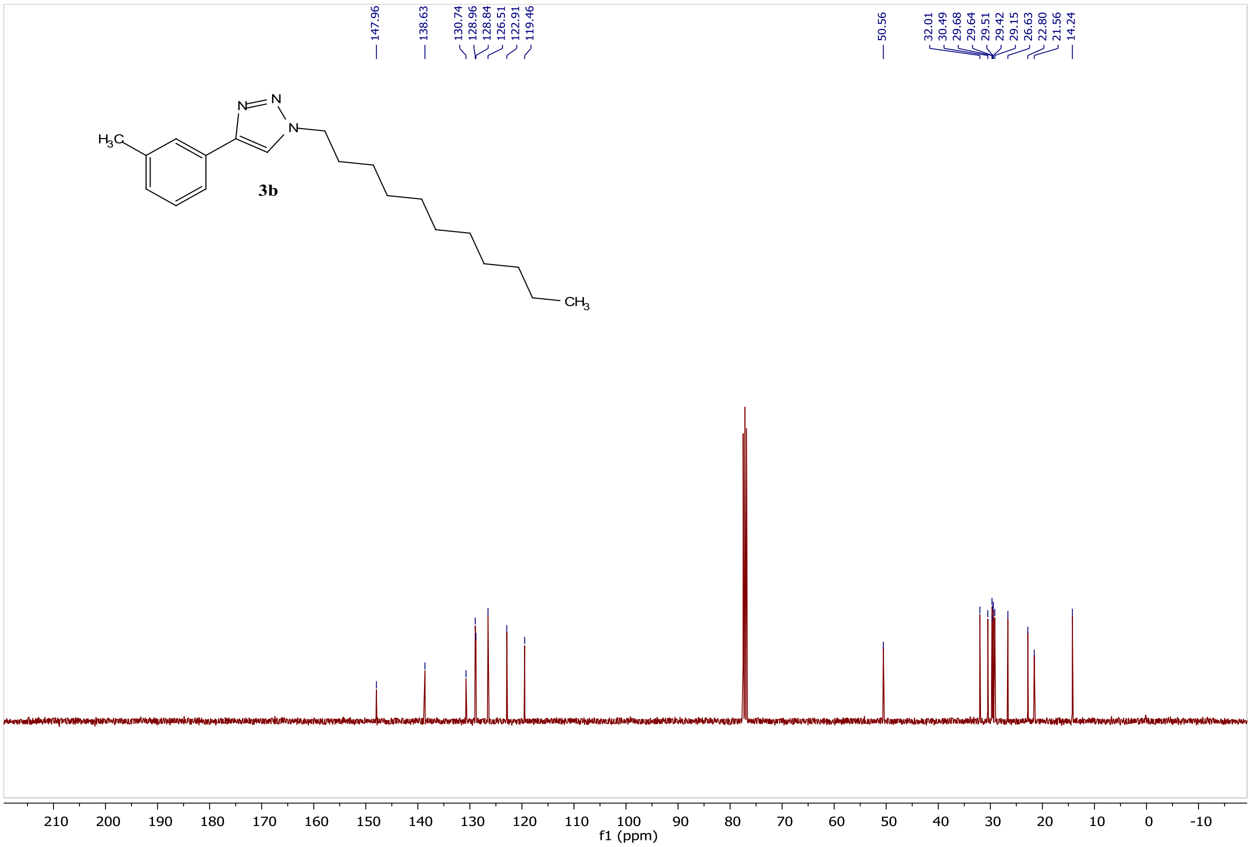
147.96
138.63
130.74
128.96
128.84
126.51
122.91
119.46
50.56
32.01
30.49
29.68
29.64
29.51
29.42
29.15
26.63
22.80
21.56
14.24
H_3C
N
N
N
3b
CH_3
f1 (ppm)

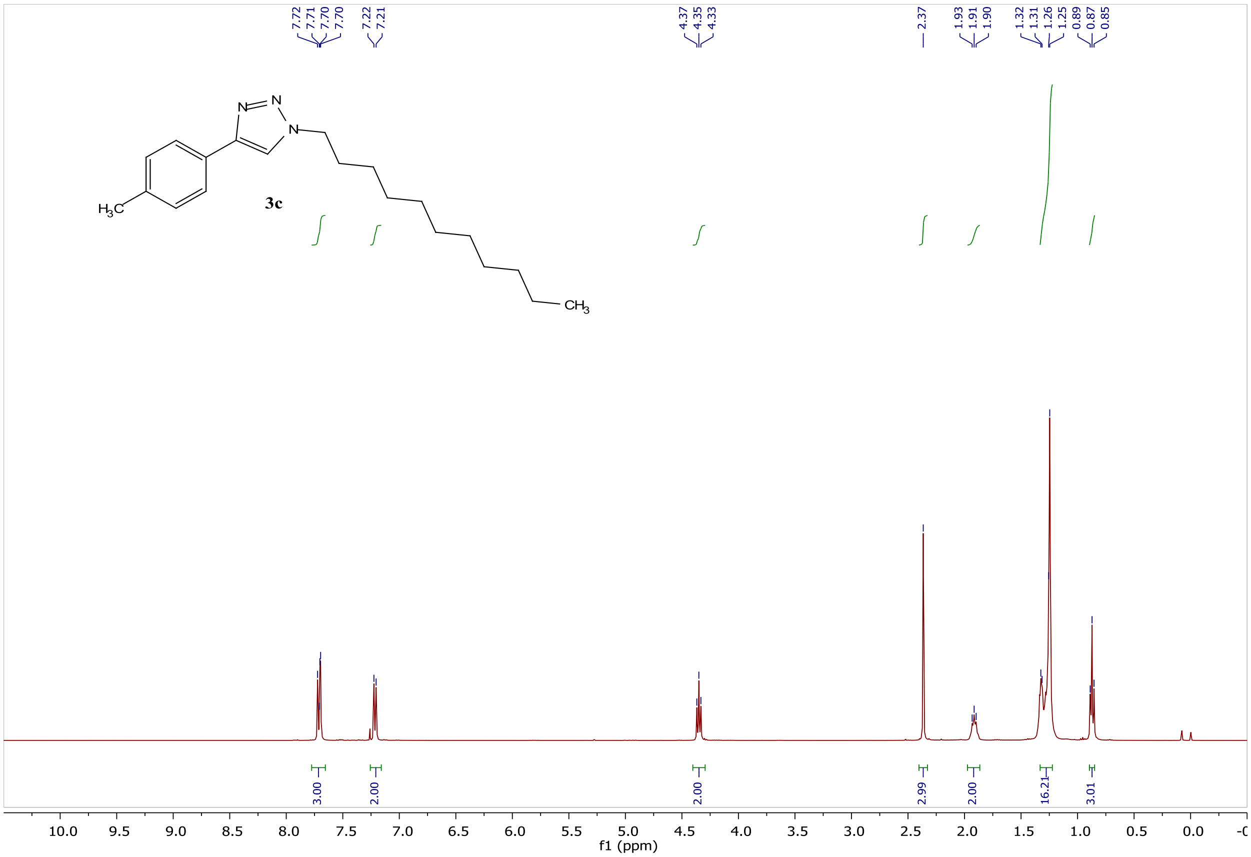
7.72
7.71
7.70
7.70
7.22
7.21
4.37
4.35
4.33
2.37
1.93
1.91
1.90
1.32
1.31
1.26
1.25
0.89
0.87
0.85
3c
3.00
2.00
2.00
2.99
2.00
16.21
3.01
f1 (ppm)

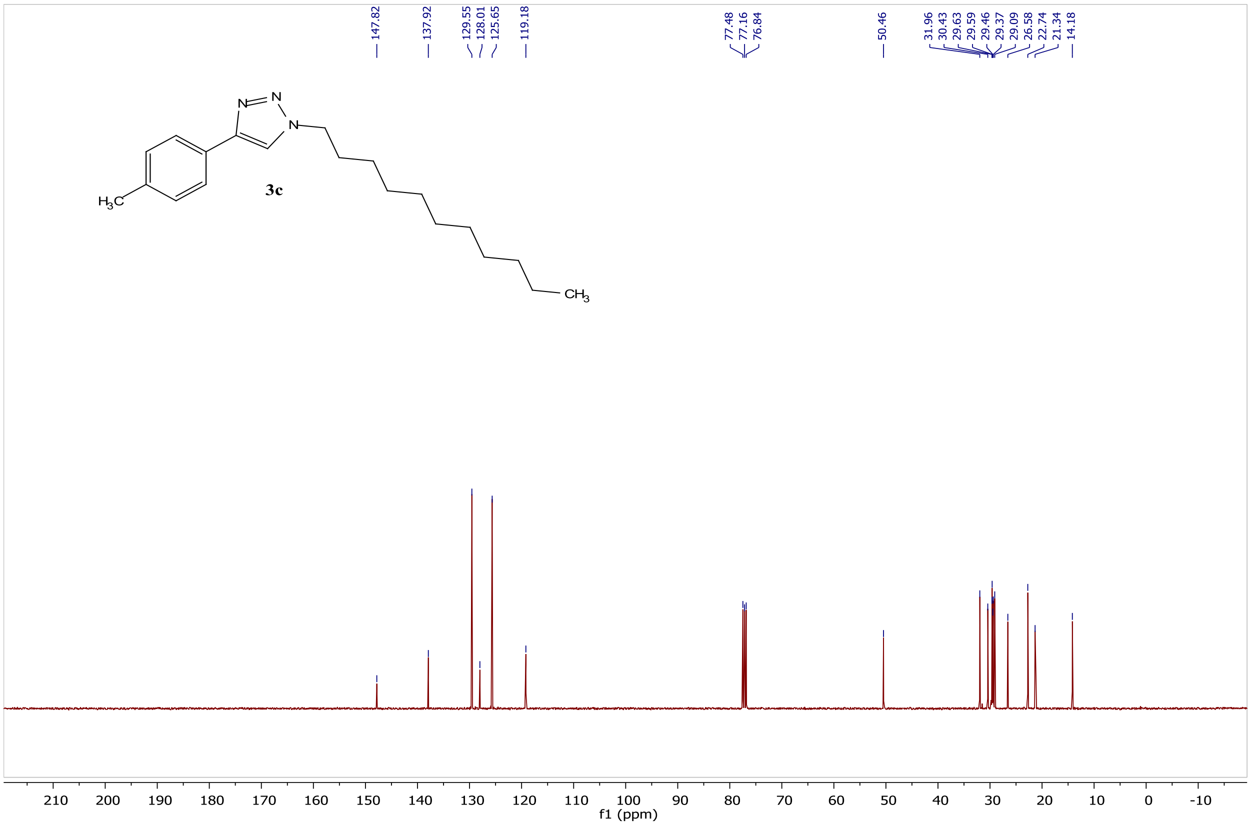
147.82
137.92
129.55
128.01
125.65
119.18
77.48
77.16
76.84
50.46
31.96
30.43
29.63
29.59
29.46
29.37
29.09
26.58
22.74
21.34
14.18
3c
f1 (ppm)

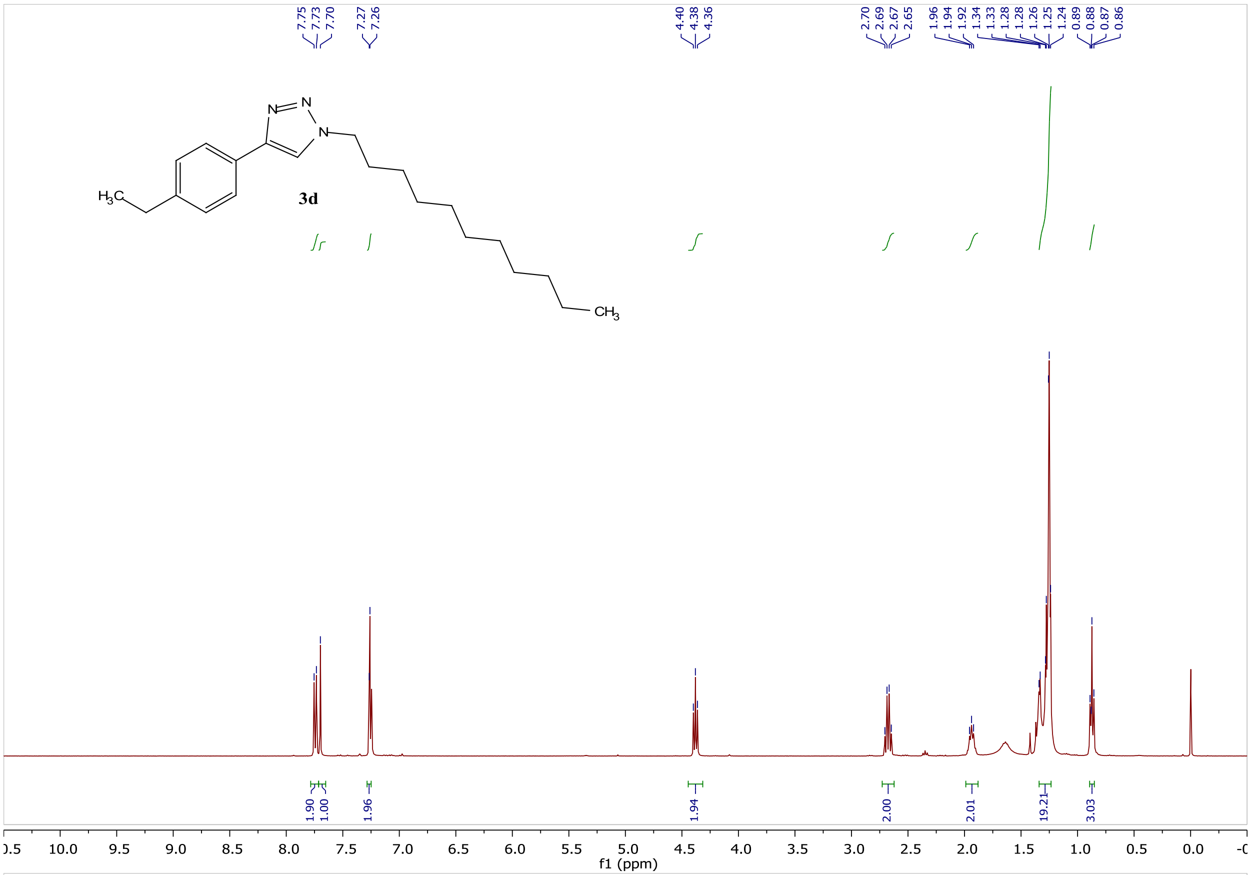

7.75
7.73
7.70
7.27
7.26
4.40
4.38
4.36
2.70
2.69
2.67
2.65
1.96
1.94
1.92
1.34
1.33
1.28
1.28
1.26
1.25
1.24
0.89
0.88
0.87
0.86
3d
1.90
1.00
1.96
1.94
2.00
2.01
19.21
3.03
f1 (ppm)

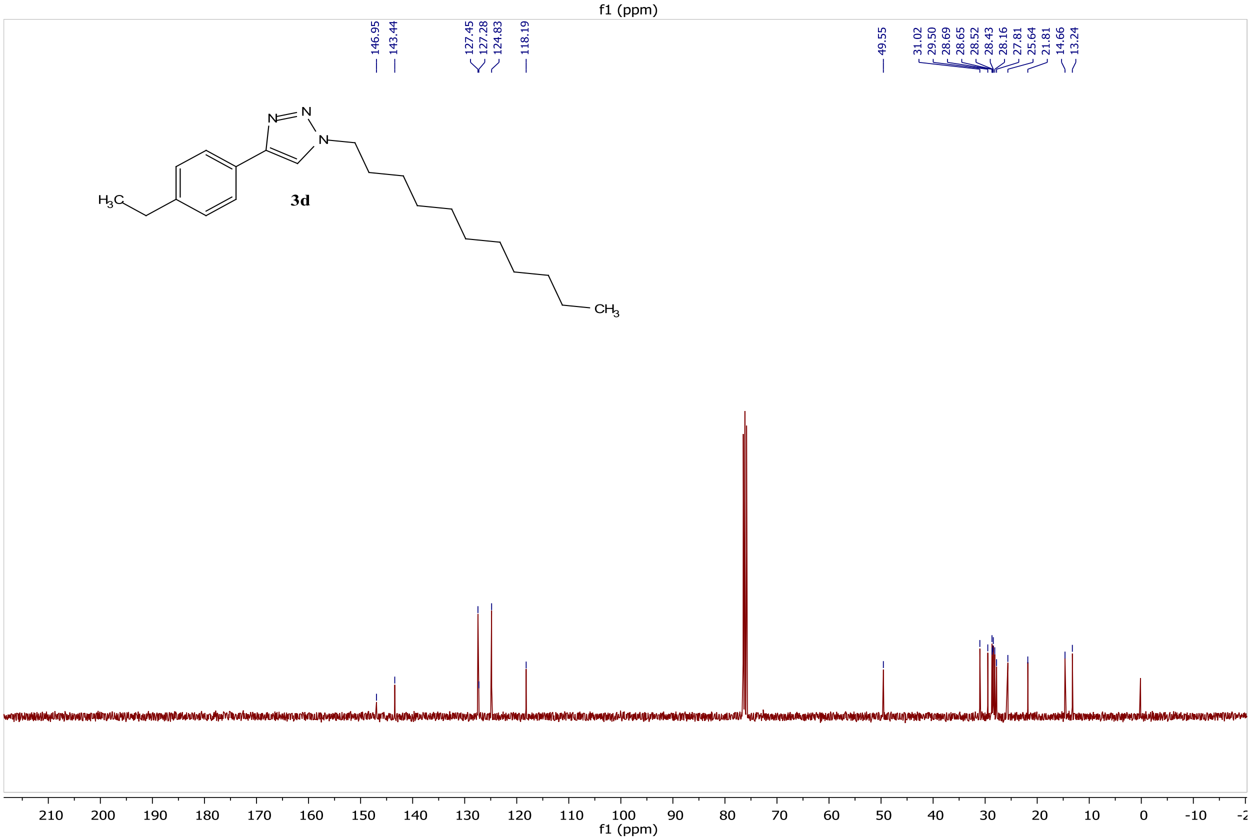

146.95
143.44
127.45
127.28
124.83
118.19
49.55
31.02
29.50
28.69
28.65
28.52
28.43
28.16
27.81
25.64
21.81
14.66
13.24
3d
f1 (ppm)

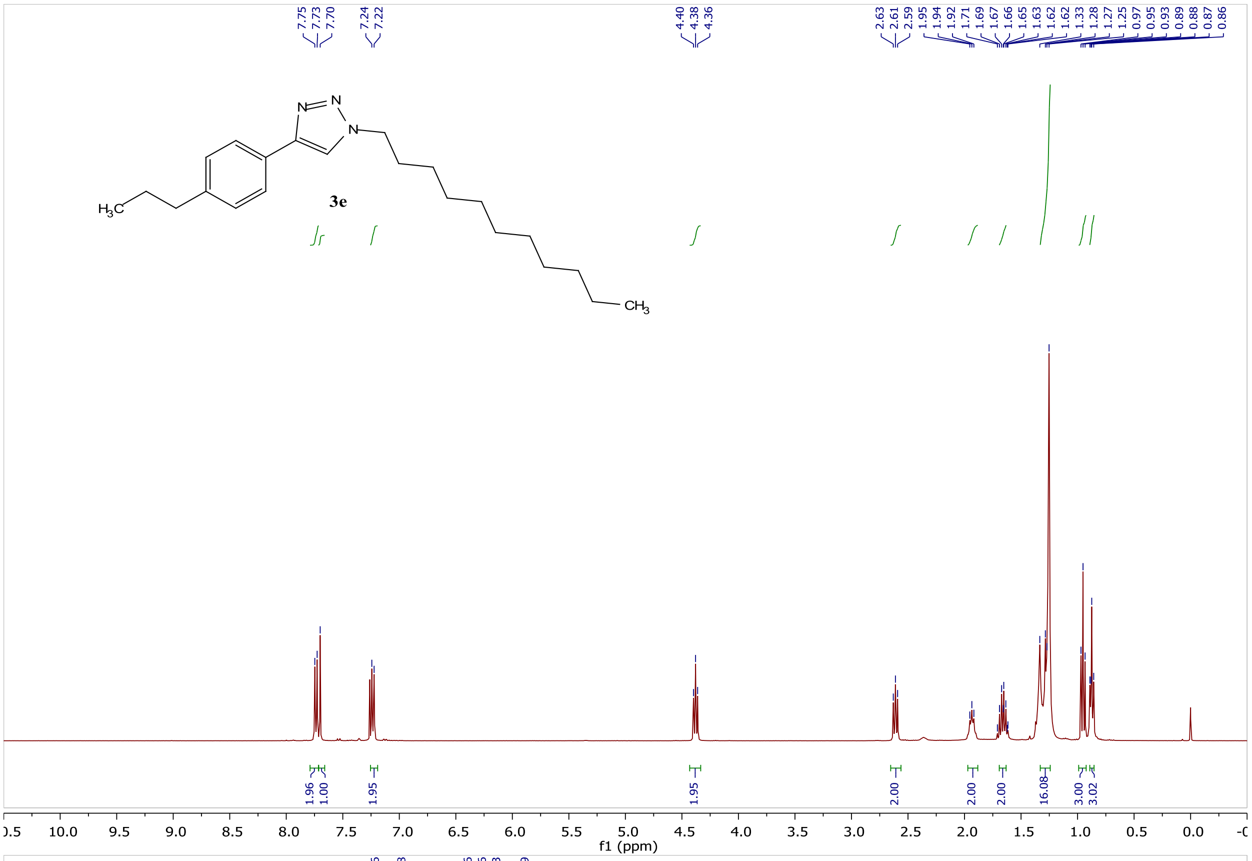
7.75
7.73
7.70
7.24
7.22
4.40
4.38
4.36
2.63
2.61
2.59
1.95
1.94
1.92
1.71
1.69
1.67
1.66
1.65
1.63
1.62
1.62
1.33
1.28
1.27
1.25
0.97
0.95
0.93
0.89
0.88
0.87
0.86
3e
1.96
1.00
1.95
1.95
2.00
2.00
2.00
16.08
3.00
3.02
f1 (ppm)

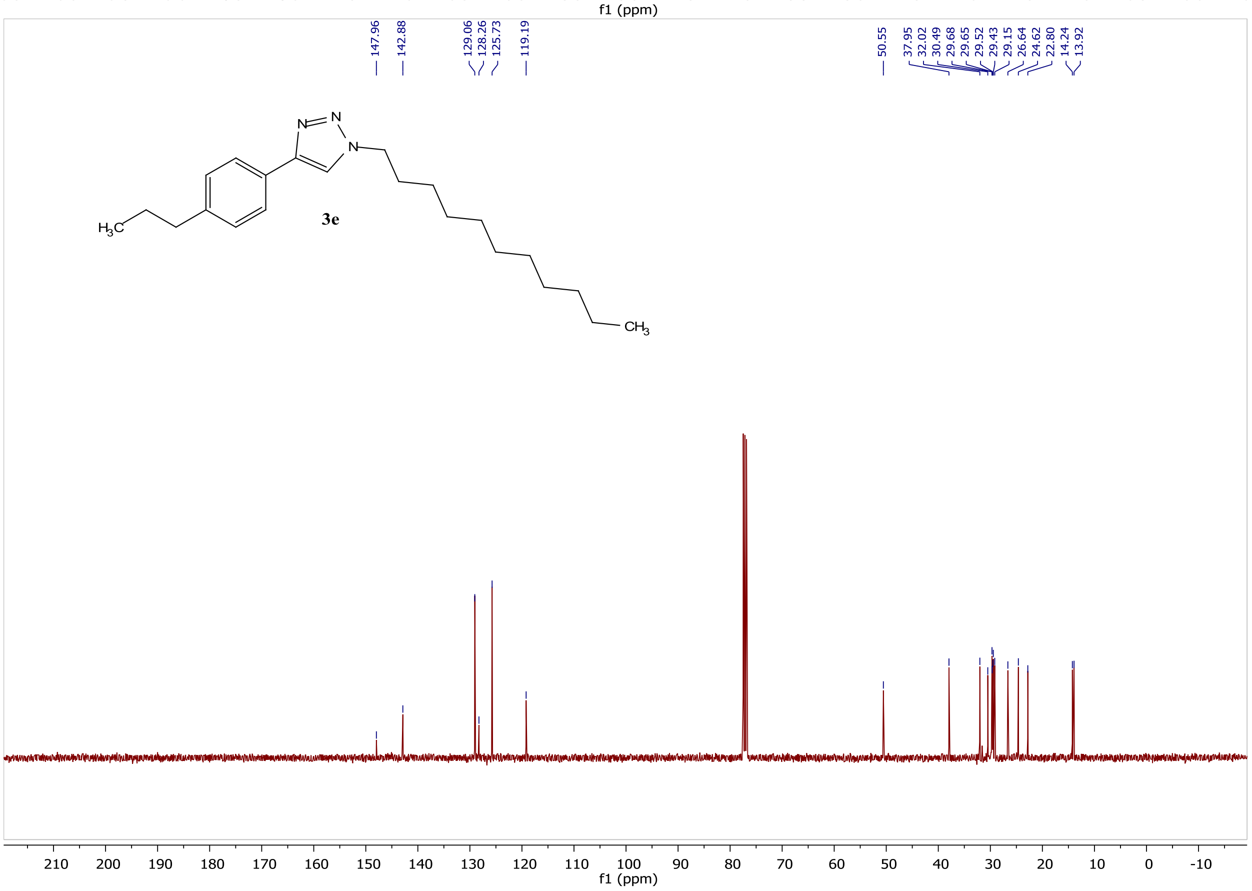
147.96
142.88
129.06
128.26
125.73
119.19
50.55
37.95
32.02
30.49
29.68
29.65
29.52
29.43
29.15
26.64
24.62
22.80
14.24
13.92
3e
f1 (ppm)

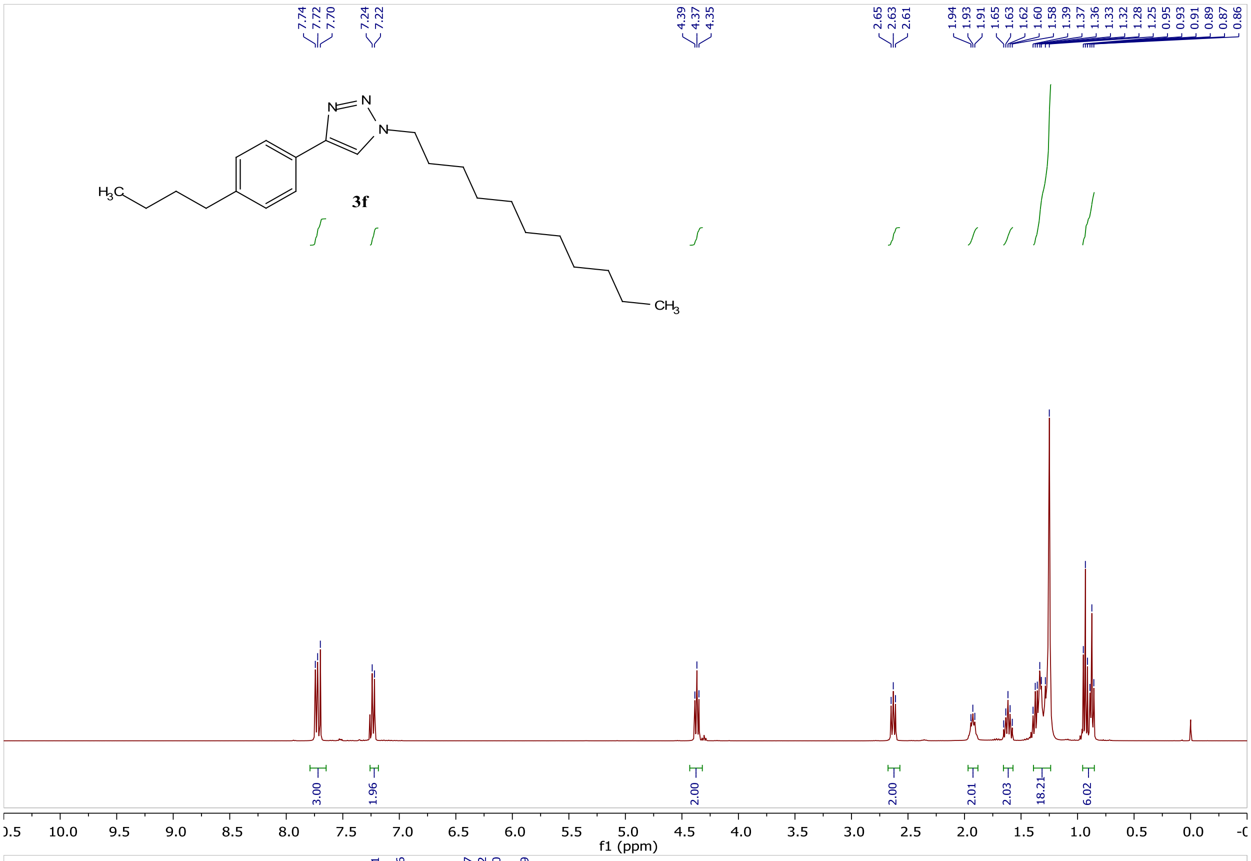
7.74
7.72
7.70
7.24
7.22
4.39
4.37
4.35
2.65
2.63
2.61
1.94
1.93
1.91
1.65
1.63
1.62
1.60
1.58
1.39
1.37
1.36
1.33
1.32
1.28
1.25
0.95
0.93
0.91
0.89
0.87
0.86
3f
3.00
1.96
2.00
2.00
2.01
2.03
18.21
6.02
f1 (ppm)

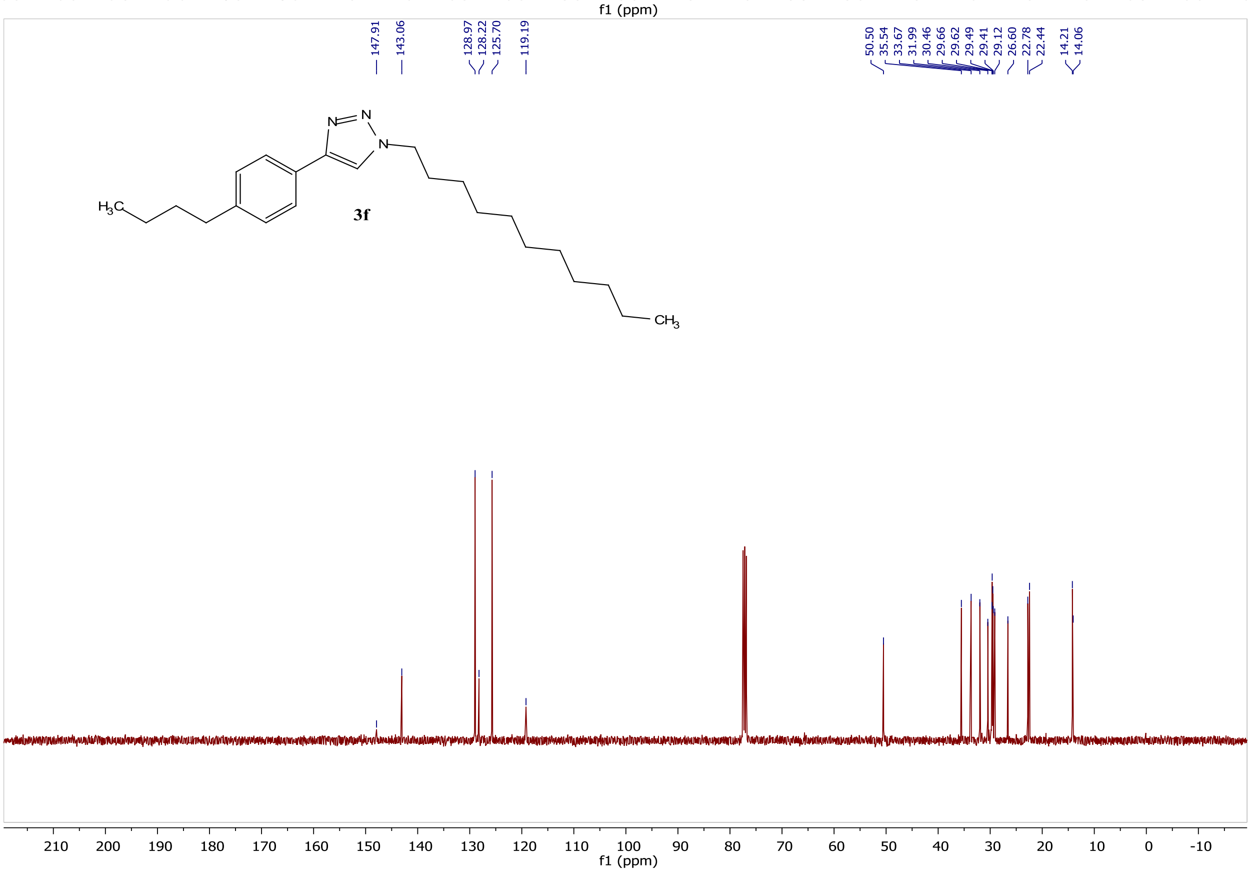
147.91
143.06
128.97
128.22
125.70
119.19
50.50
35.54
33.67
31.99
30.46
29.66
29.62
29.49
29.41
29.12
26.60
22.78
22.44
14.21
14.06
3f
f1 (ppm)

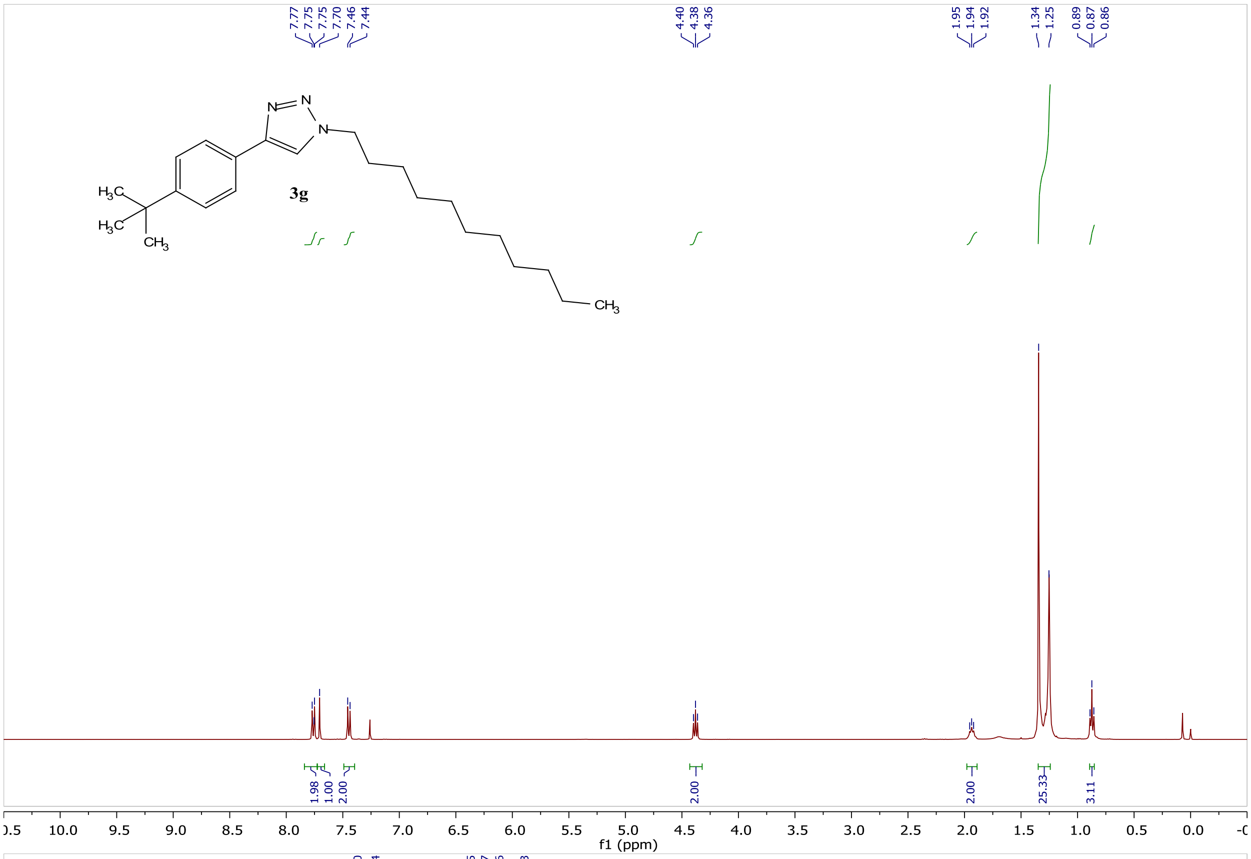
7.77
7.75
7.75
7.70
7.46
7.44
4.40
4.38
4.36
1.95
1.94
1.92
1.34
1.25
0.89
0.87
0.86
3g
1.98
1.00
2.00
2.00
2.00
25.33
3.11
f1 (ppm)

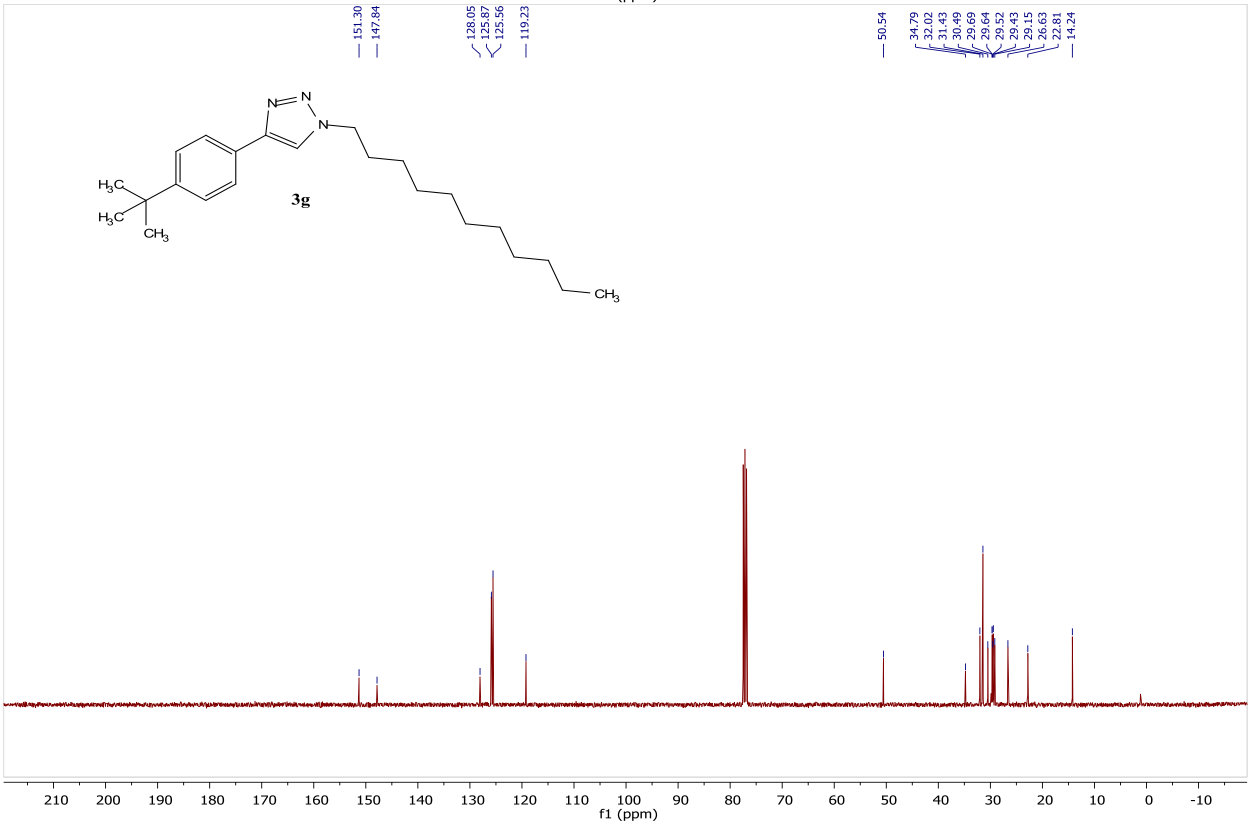
151.30
147.84
128.05
125.87
125.56
119.23
50.54
34.79
32.02
31.43
30.49
29.69
29.64
29.52
29.43
29.15
26.63
22.81
14.24
3g
f1 (ppm)

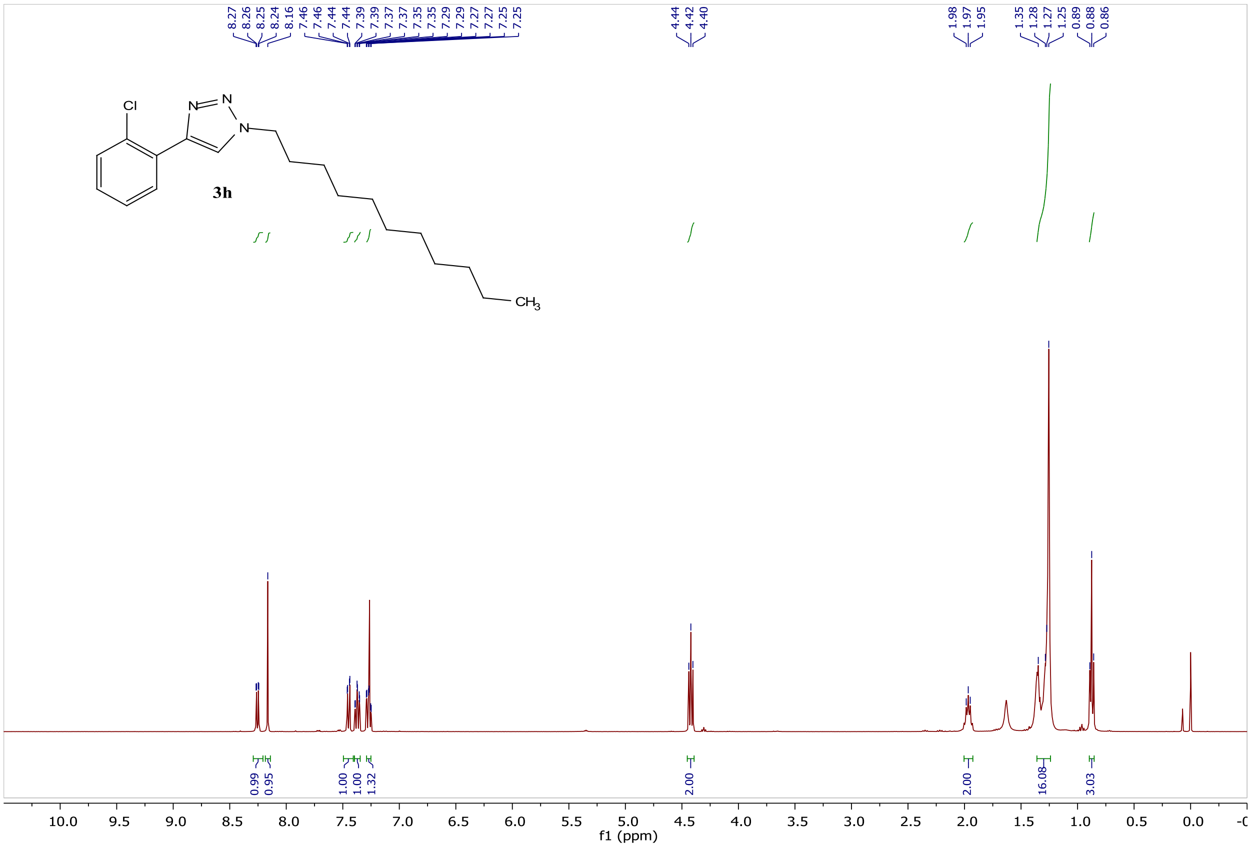
8.27
8.26
8.25
8.24
8.16
7.46
7.46
7.44
7.44
7.39
7.39
7.37
7.37
7.35
7.35
7.29
7.29
7.27
7.27
7.25
7.25
4.44
4.42
4.40
1.98
1.97
1.95
1.35
1.28
1.27
1.25
0.89
0.88
0.86
Cl
N
N
N
3h
CH3
0.99
0.95
1.00
1.00
1.32
2.00
2.00
16.08
3.03
10.0 9.5 9.0 8.5 8.0 7.5 7.0 6.5 6.0 5.5 5.0 4.5 4.0 3.5 3.0 2.5 2.0 1.5 1.0 0.5 0.0
f1 (ppm)

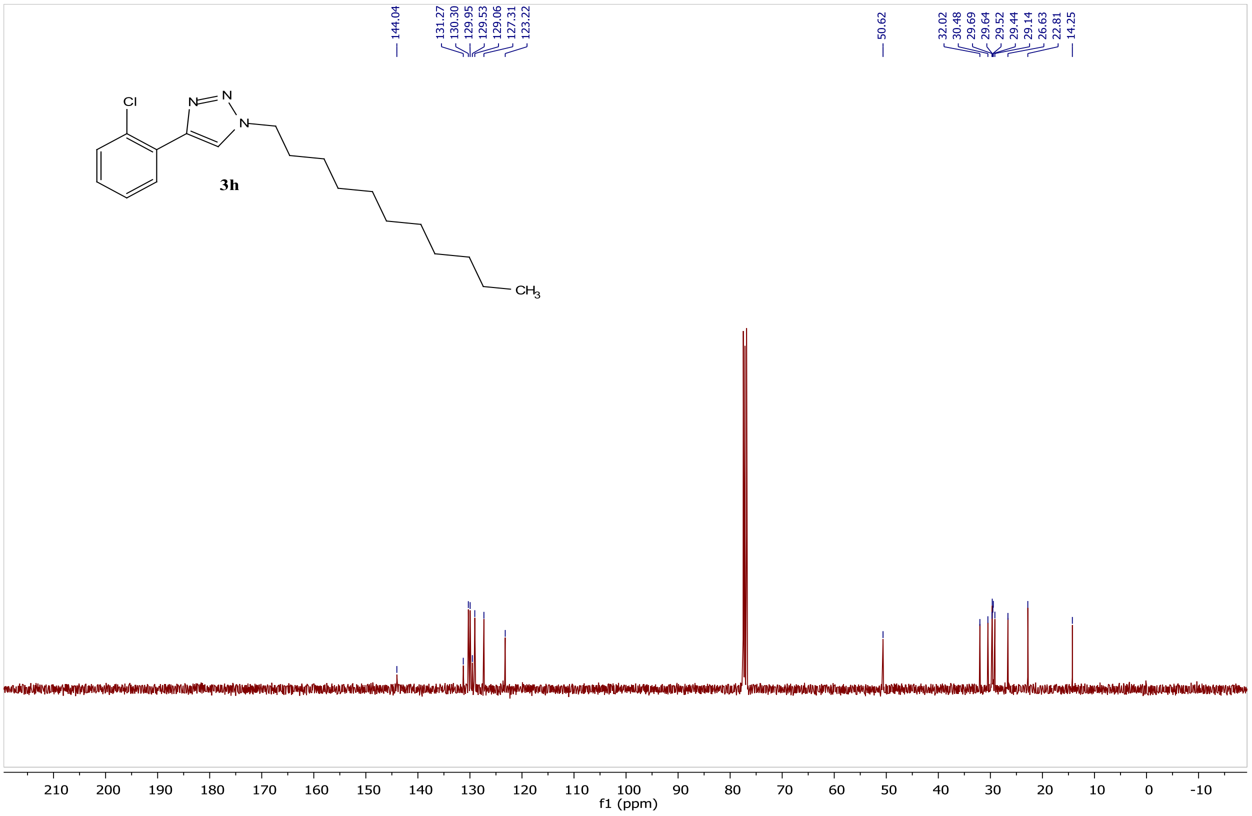
144.04
131.27
130.30
129.95
129.53
129.06
127.31
123.22
50.62
32.02
30.48
29.69
29.64
29.52
29.44
29.14
26.63
22.81
14.25
Cl
N
N
N
3h
CH3
210 200 190 180 170 160 150 140 130 120 110 100 90 80 70 60 50 40 30 20 10 0 -10
f1 (ppm)

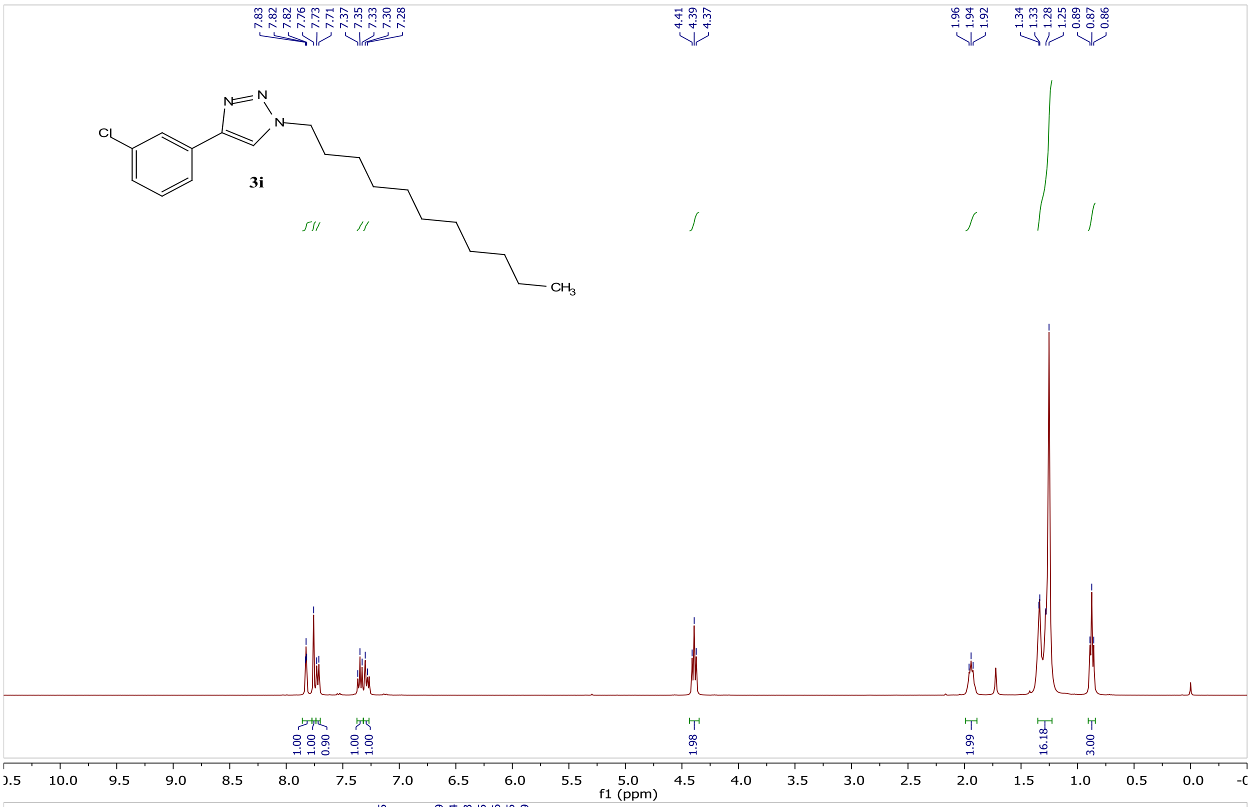

7.83
7.82
7.82
7.76
7.73
7.71
7.37
7.35
7.33
7.30
7.28
4.41
4.39
4.37
1.96
1.94
1.92
1.34
1.33
1.28
1.25
0.89
0.87
0.86
Cl
N
N
N
3i
CH3
1.00
1.00
0.90
1.00
1.00
1.98
1.99
16.18
3.00
f1 (ppm)

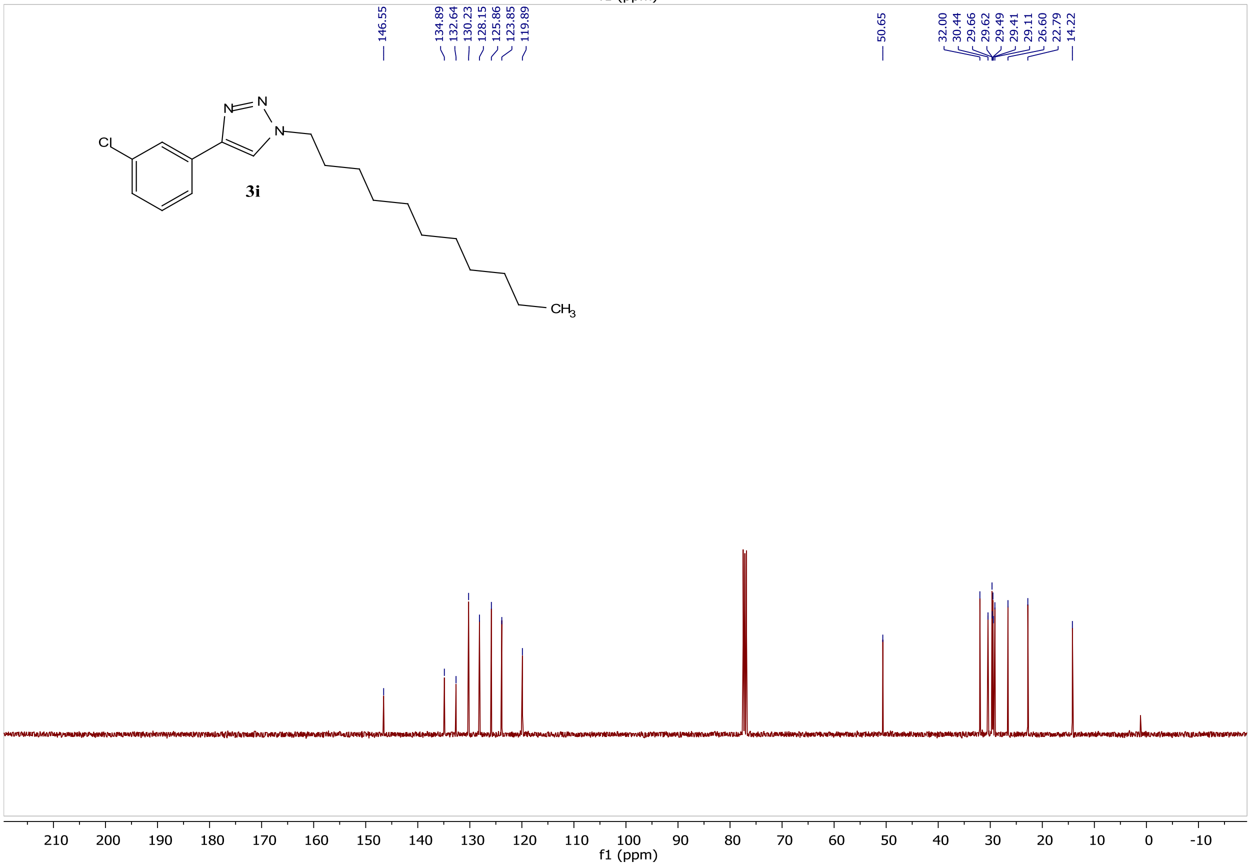

146.55
134.89
132.64
130.23
128.15
125.86
123.85
119.89
50.65
32.00
30.44
29.66
29.62
29.49
29.41
29.11
26.60
22.79
14.22
Cl
N
N
N
3i
CH3
f1 (ppm)

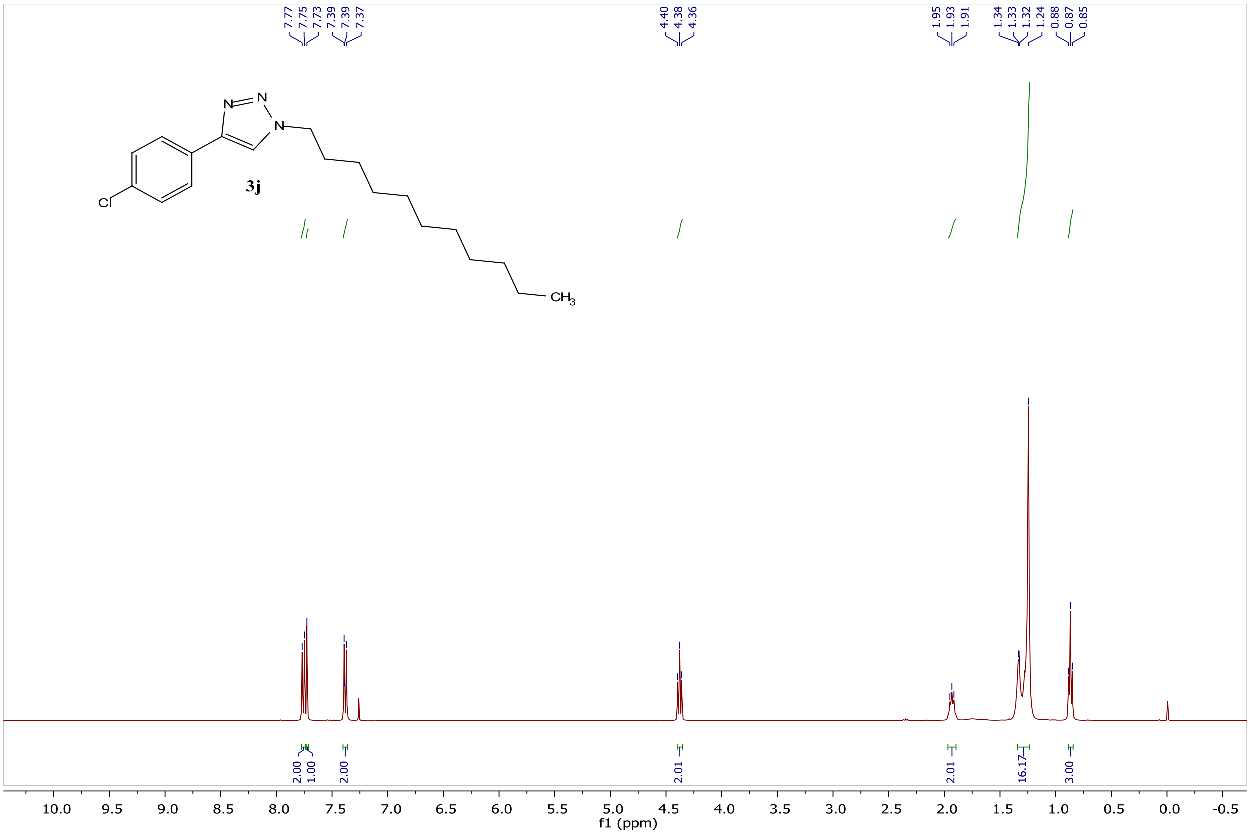
7.77
7.75
7.73
7.39
7.39
7.37
4.40
4.38
4.36
1.95
1.93
1.91
1.34
1.33
1.32
1.24
0.88
0.87
0.85
3j
Cl
N
N
N
CH3
2.00
1.00
2.00
2.01
2.01
16.17
3.00
10.0 9.5 9.0 8.5 8.0 7.5 7.0 6.5 6.0 5.5 5.0 4.5 4.0 3.5 3.0 2.5 2.0 1.5 1.0 0.5 0.0 -0.5
f1 (ppm)

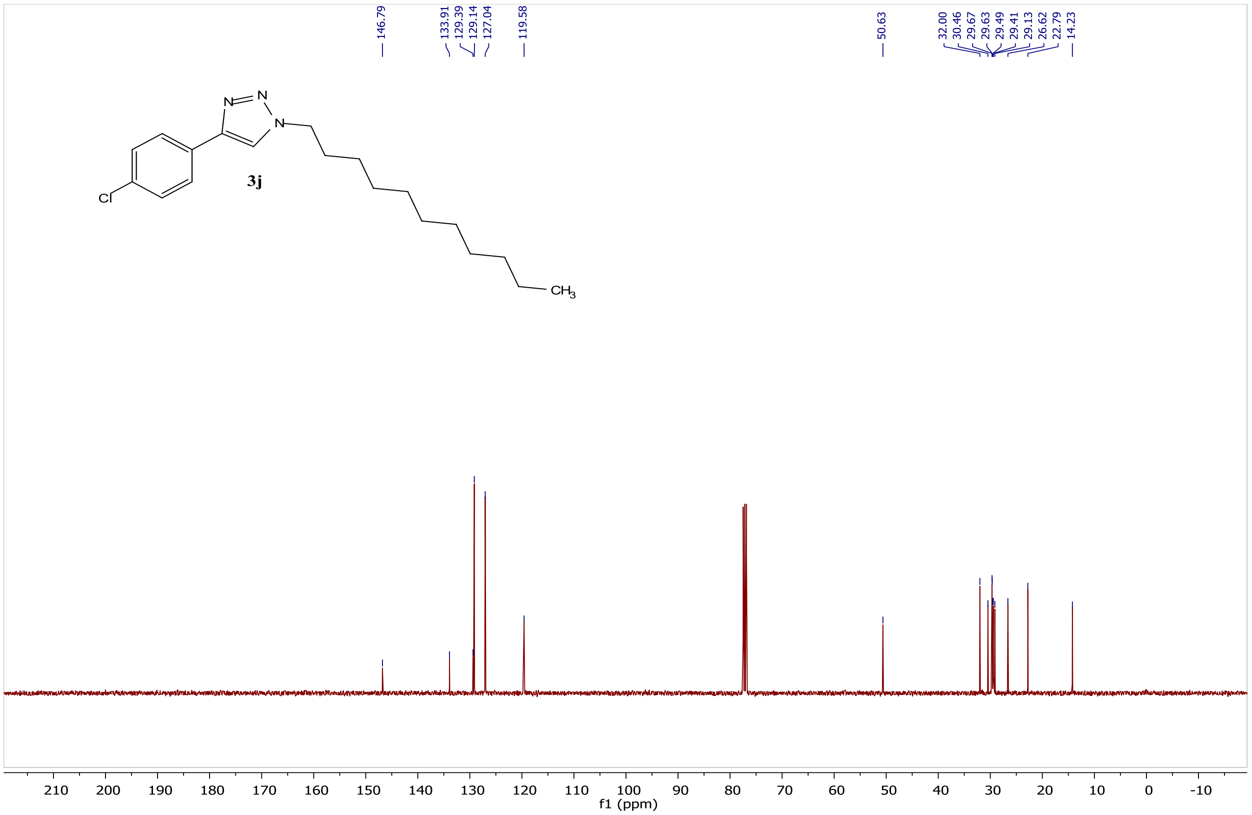
146.79
133.91
129.39
129.14
127.04
119.58
50.63
32.00
30.46
29.67
29.63
29.49
29.41
29.13
26.62
22.79
14.23
3j
Cl
N
N
N
CH3
210 200 190 180 170 160 150 140 130 120 110 100 90 80 70 60 50 40 30 20 10 0 -10
f1 (ppm)

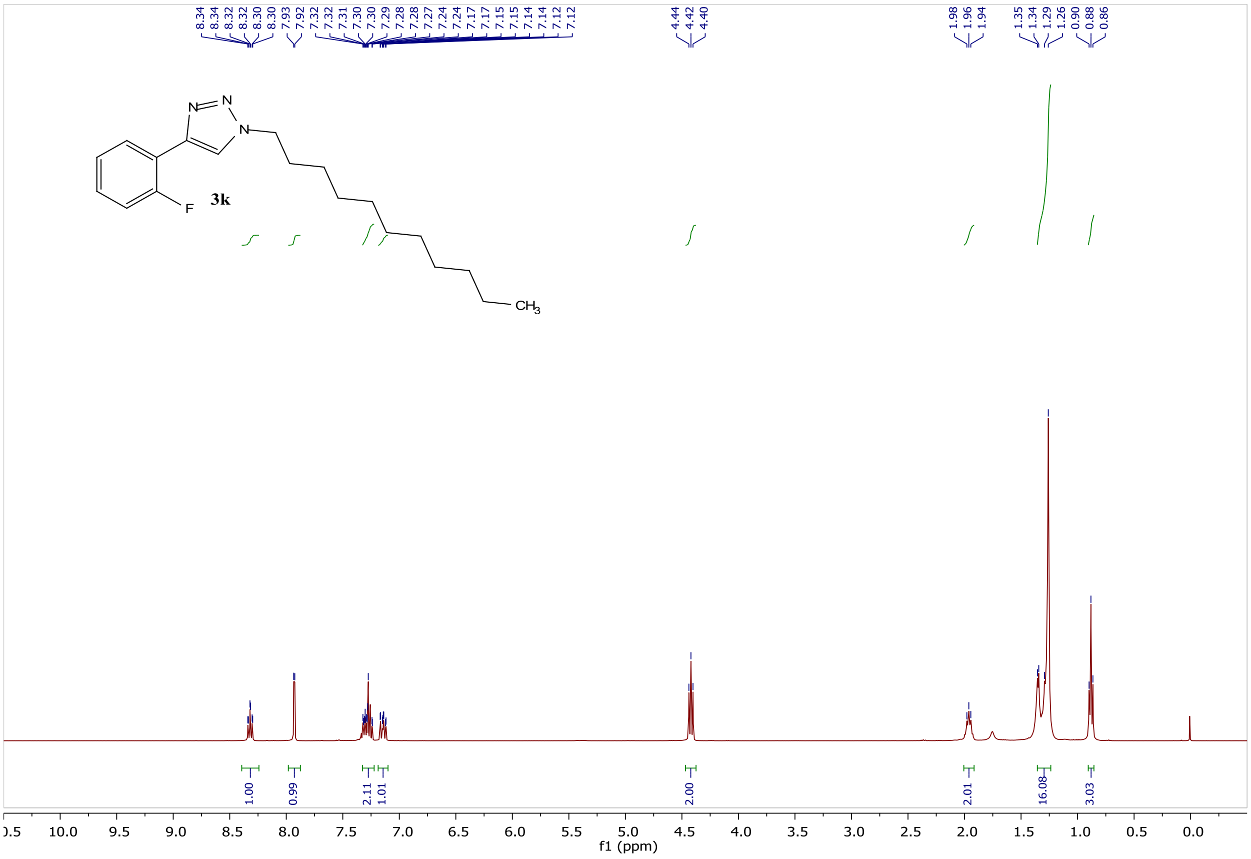
8.34
8.34
8.32
8.32
8.30
8.30
7.93
7.92
7.32
7.32
7.31
7.30
7.30
7.29
7.28
7.28
7.27
7.24
7.24
7.17
7.17
7.15
7.15
7.14
7.14
7.12
7.12
4.44
4.42
4.40
1.98
1.96
1.94
1.35
1.34
1.29
1.26
0.90
0.88
0.86
3k
CH3
1.00
0.99
2.11
1.01
2.00
2.01
16.08
3.03
f1 (ppm)

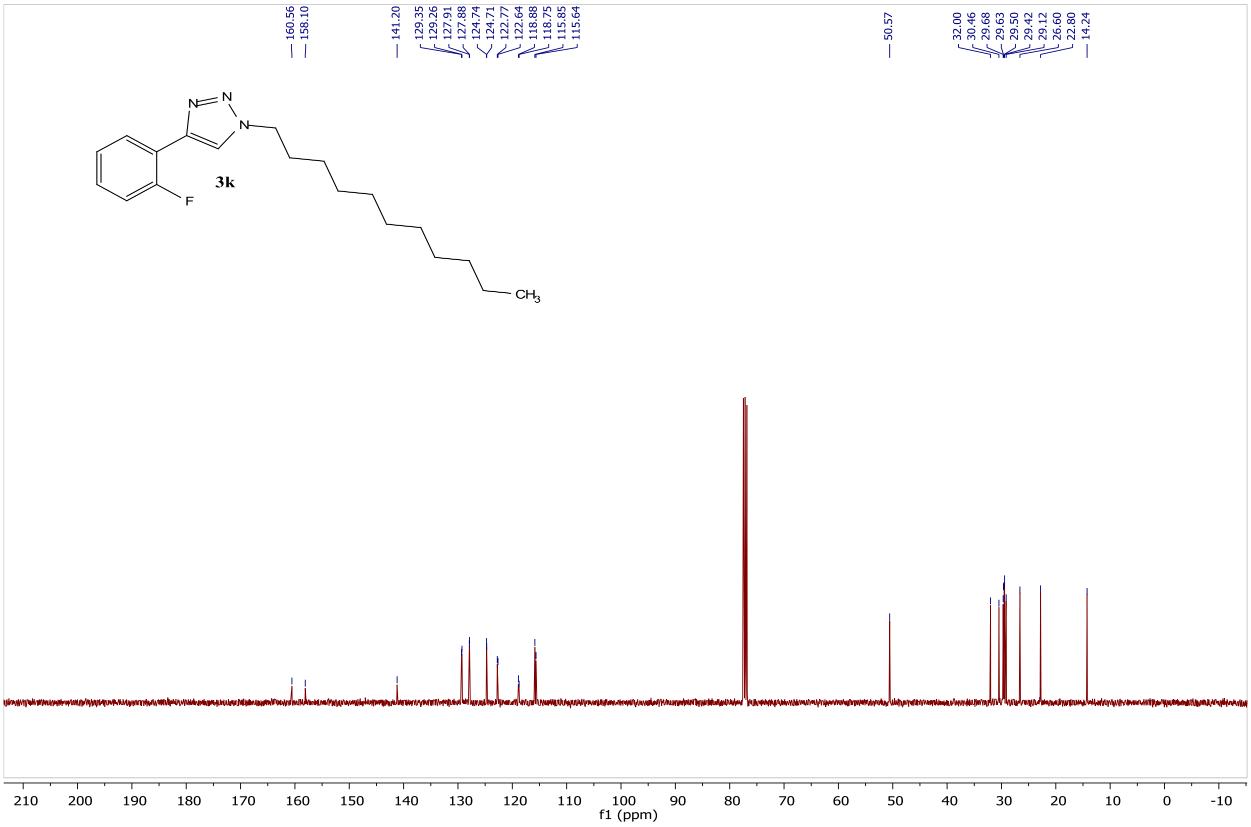
160.56
158.10
141.20
129.35
129.26
127.91
127.88
124.74
124.71
122.77
122.64
118.88
118.75
115.85
115.64
50.57
32.00
30.46
29.68
29.63
29.50
29.42
29.12
26.60
22.80
14.24
3k
CH3
f1 (ppm)

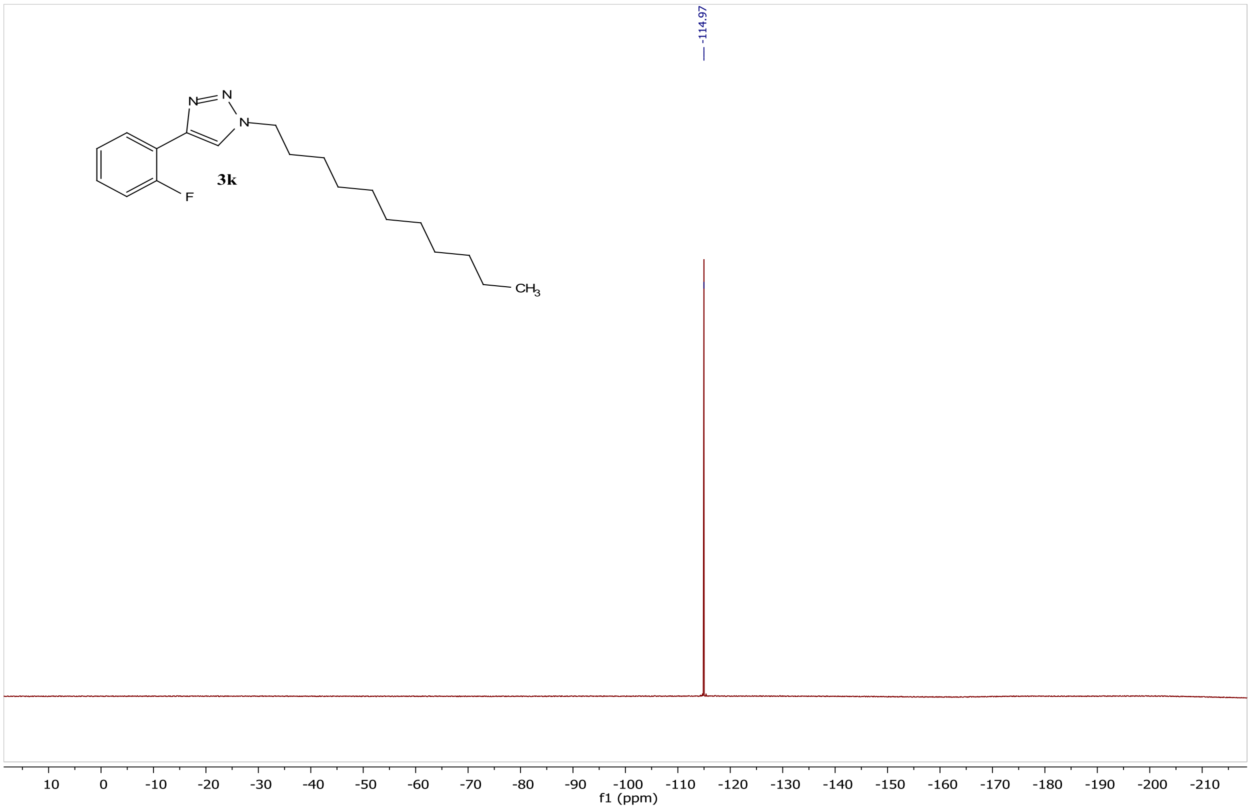
-114.97
N=N
N
3k
F
CH3
10 0 -10 -20 -30 -40 -50 -60 -70 -80 -90 -100 -110 -120 -130 -140 -150 -160 -170 -180 -190 -200 -210
f1 (ppm)

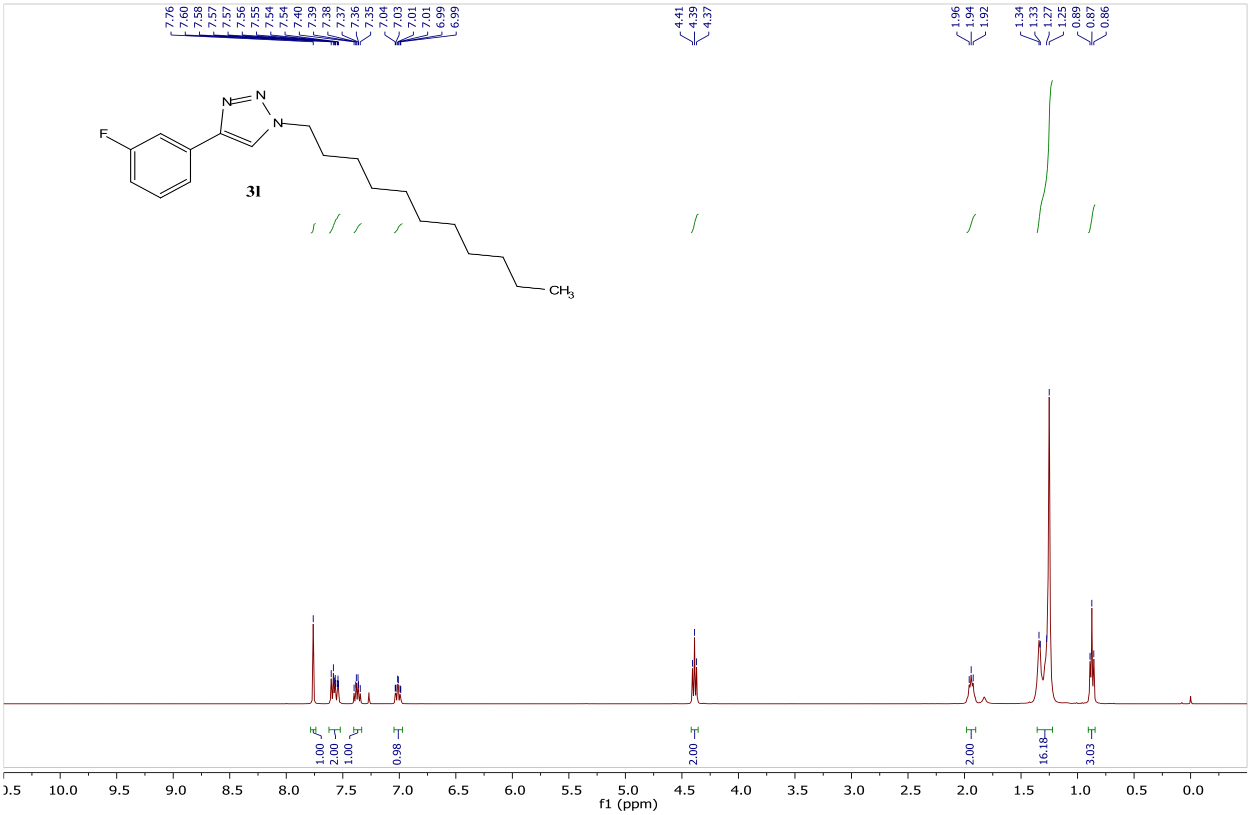
7.76 7.60 7.58 7.57 7.57 7.56 7.55 7.54 7.54 7.40 7.39 7.38 7.37 7.36 7.35 7.04 7.03 7.01 7.01 6.99 6.99
4.41 4.39 4.37
1.96 1.94 1.92
1.34 1.33 1.27 1.25 0.89 0.87 0.86
N=N
N
F
3l
CH3
1.00 2.00 1.00 0.98 2.00 2.00 16.18 3.03
10.0 9.5 9.0 8.5 8.0 7.5 7.0 6.5 6.0 5.5 5.0 4.5 4.0 3.5 3.0 2.5 2.0 1.5 1.0 0.5 0.0
f1 (ppm)

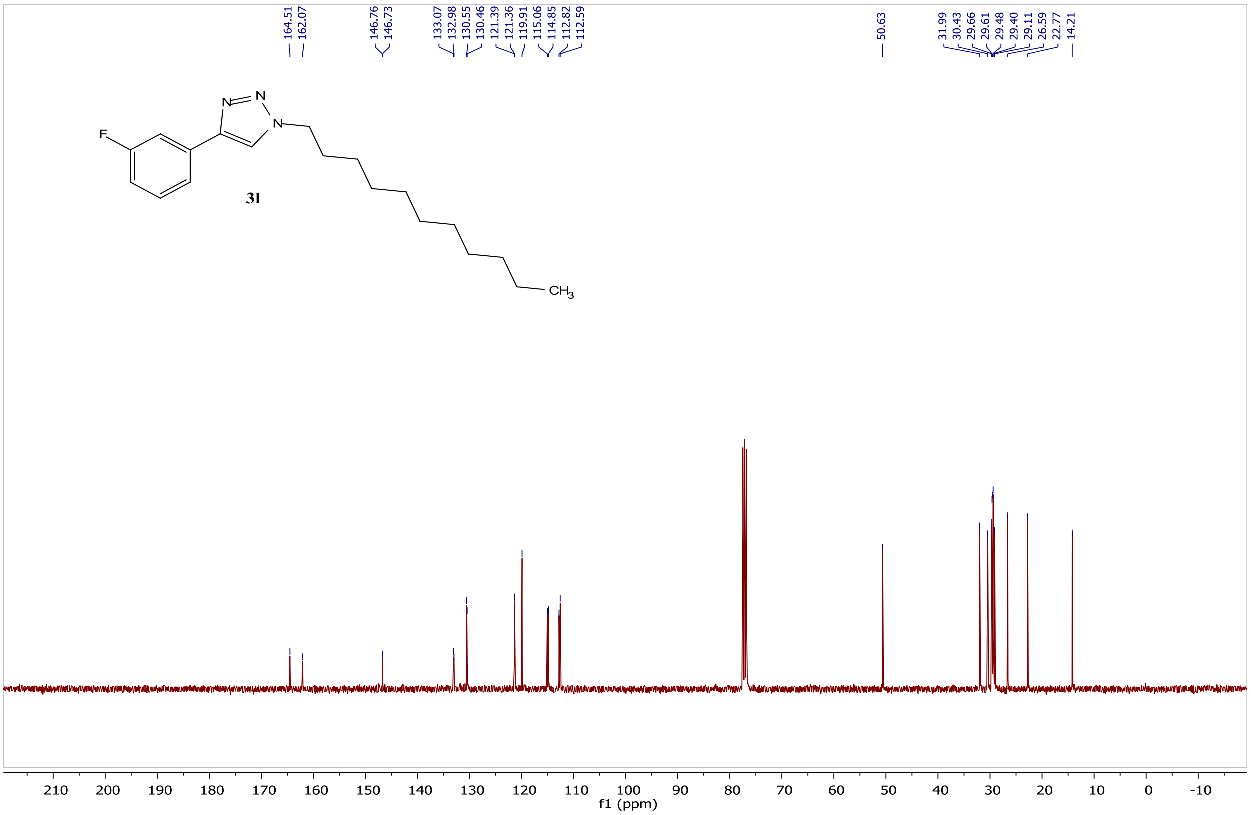

164.51
162.07
146.76
146.73
133.07
132.98
130.55
130.46
121.39
121.36
119.91
115.06
114.85
112.82
112.59
50.63
31.99
30.43
29.66
29.61
29.48
29.40
29.11
26.59
22.77
14.21
F
N
N
N
3l
CH3
f1 (ppm)

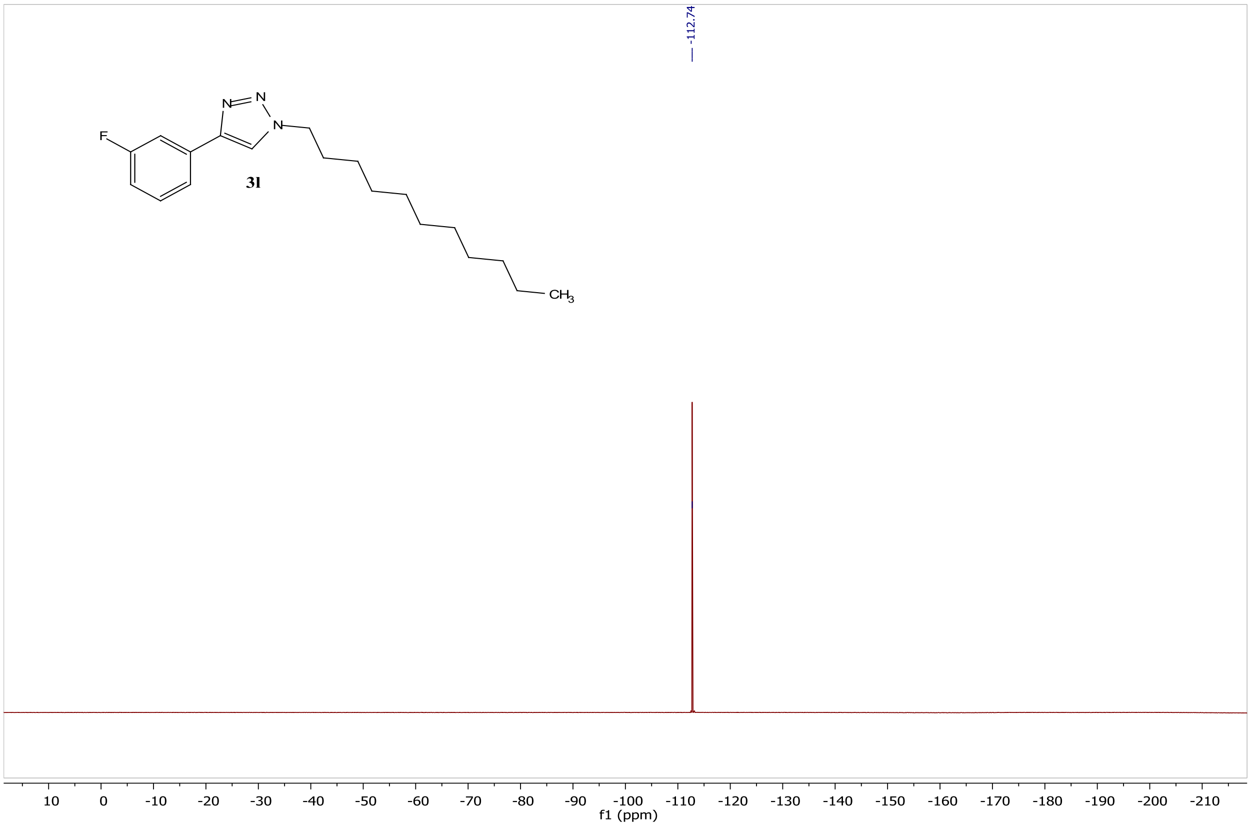

-112.74
F
N
N
N
3l
CH3
f1 (ppm)

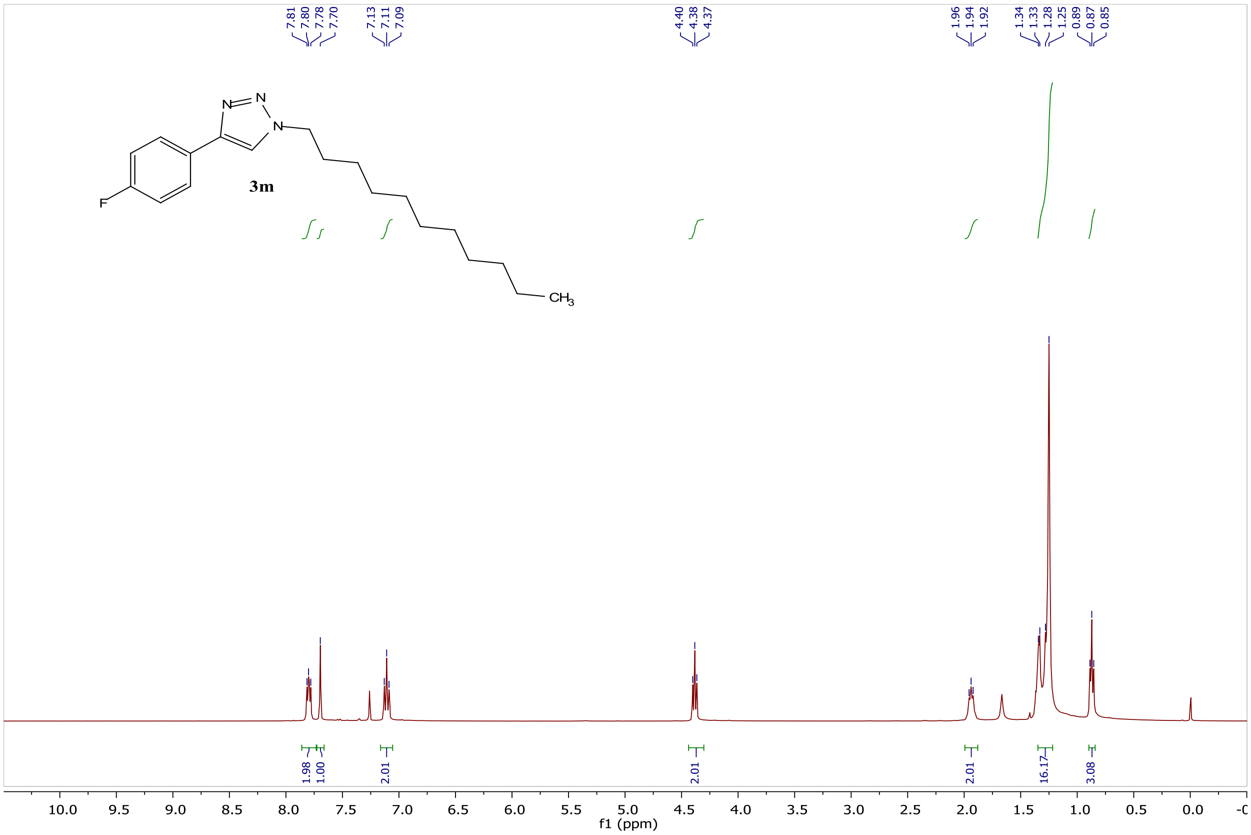
7.81
7.80
7.78
7.70
7.13
7.11
7.09
4.40
4.38
4.37
1.96
1.94
1.92
1.34
1.33
1.28
1.25
0.89
0.87
0.85
3m
F
N
N
N
CH3
1.98
1.00
2.01
2.01
2.01
16.17
3.08
10.0 9.5 9.0 8.5 8.0 7.5 7.0 6.5 6.0 5.5 5.0 4.5 4.0 3.5 3.0 2.5 2.0 1.5 1.0 0.5 0.0
f1 (ppm)

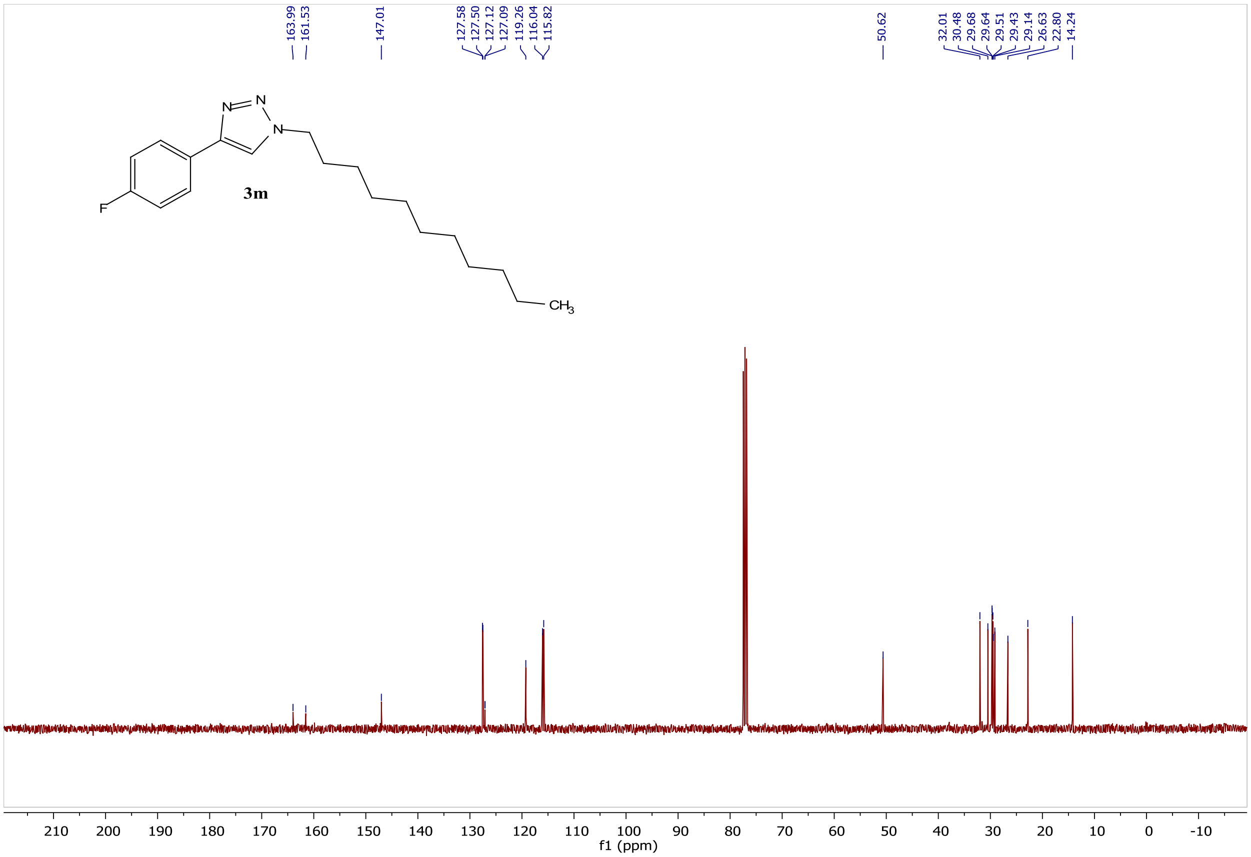
163.99
161.53
147.01
127.58
127.50
127.12
127.09
119.26
116.04
115.82
50.62
32.01
30.48
29.68
29.64
29.51
29.43
29.14
26.63
22.80
14.24
3m
F
N
N
N
CH3
210 200 190 180 170 160 150 140 130 120 110 100 90 80 70 60 50 40 30 20 10 0 -10
f1 (ppm)

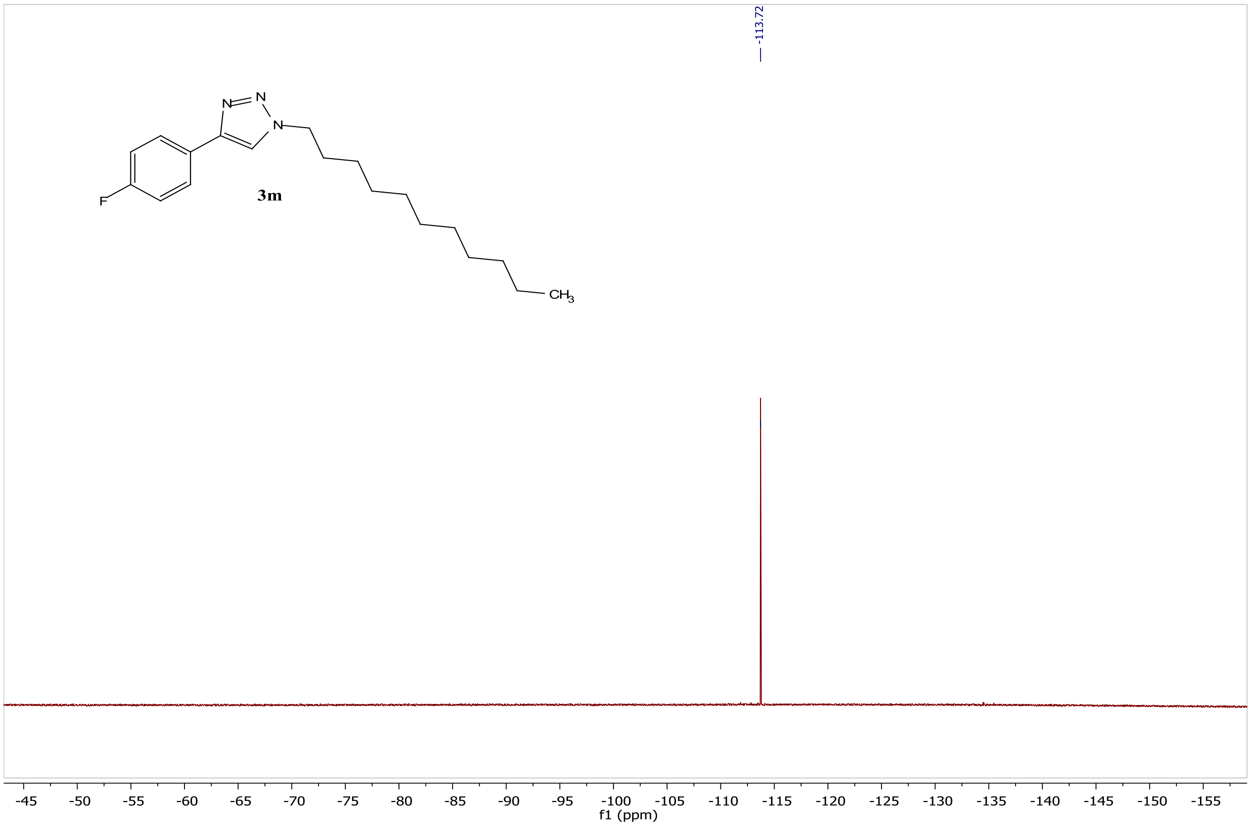

3m
CH3
-113.72
-45
-50
-55
-60
-65
-70
-75
-80
-85
-90
-95
-100
-105
-110
-115
-120
-125
-130
-135
-140
-145
-150
-155
f1 (ppm)

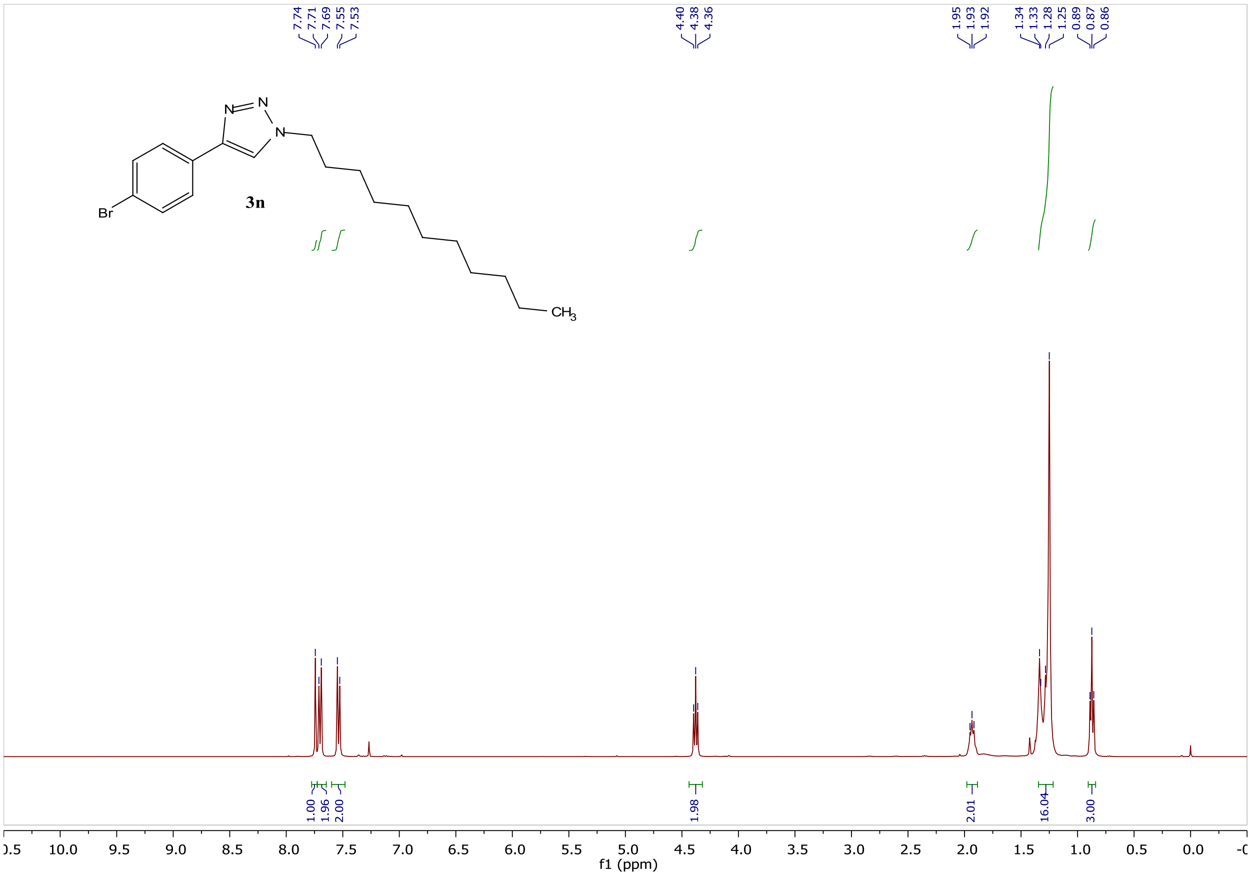
7.74
7.71
7.69
7.55
7.53
4.40
4.38
4.36
1.95
1.93
1.92
1.34
1.33
1.28
1.25
0.89
0.87
0.86
N
N
N
Br
3n
CH3
1.00
1.96
2.00
1.98
2.01
16.04
3.00
f1 (ppm)

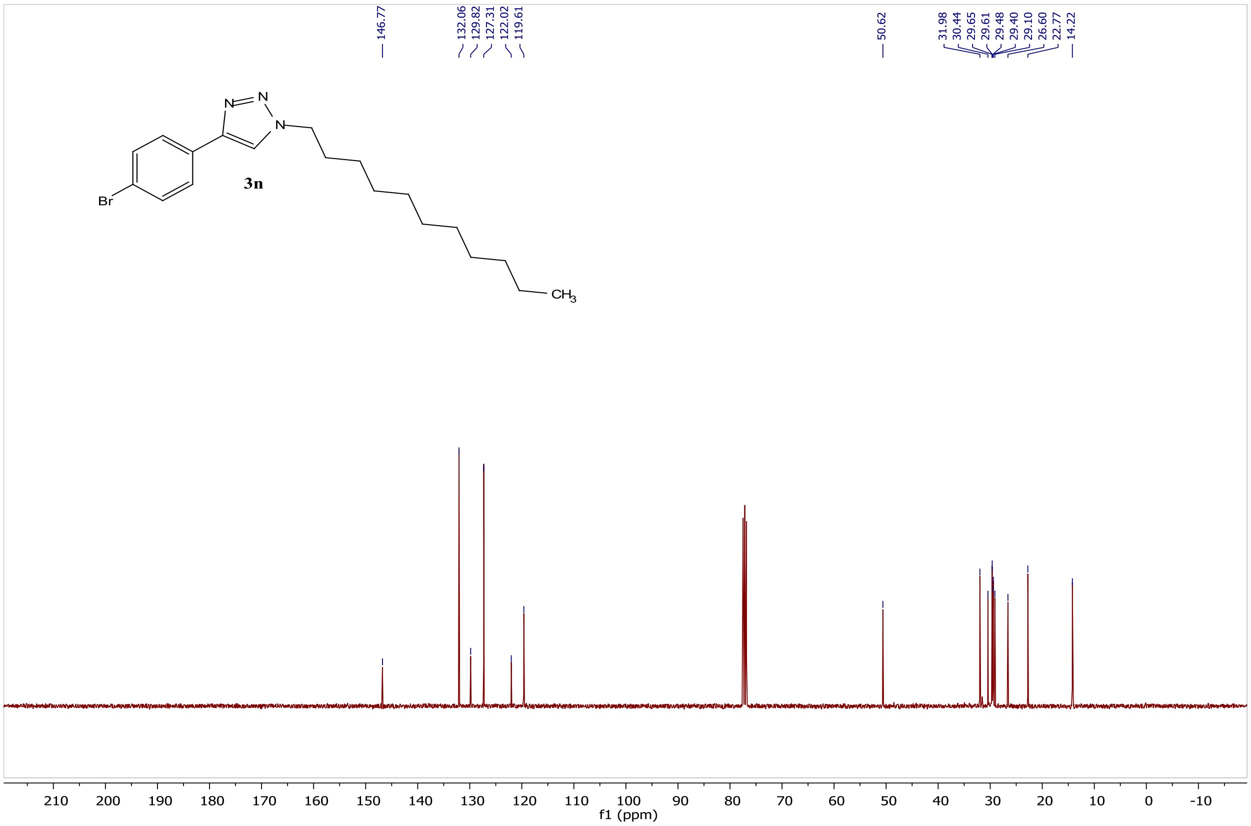
146.77
132.06
129.82
127.31
122.02
119.61
50.62
31.98
30.44
29.65
29.61
29.48
29.40
29.10
26.60
22.77
14.22
N
N
N
Br
3n
CH3
f1 (ppm)

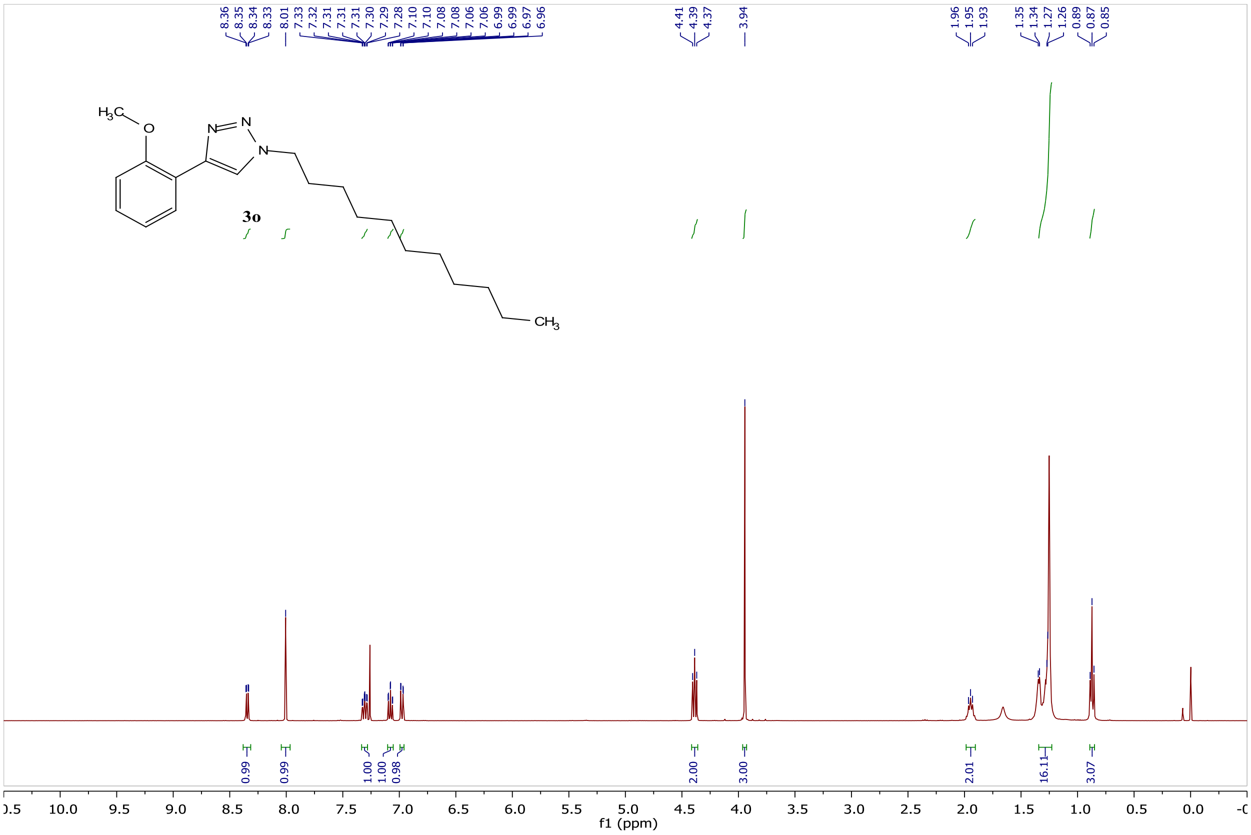
8.36
8.35
8.34
8.33
8.01
7.33
7.32
7.31
7.31
7.31
7.30
7.29
7.28
7.10
7.10
7.08
7.08
7.06
7.06
6.99
6.99
6.97
6.96
4.41
4.39
4.37
3.94
1.96
1.95
1.93
1.35
1.34
1.27
1.26
0.89
0.87
0.85
H_3C
O
N
N
N
3o
CH_3
0.99
0.99
1.00
1.00
0.98
2.00
3.00
2.01
16.11
3.07
f1 (ppm)

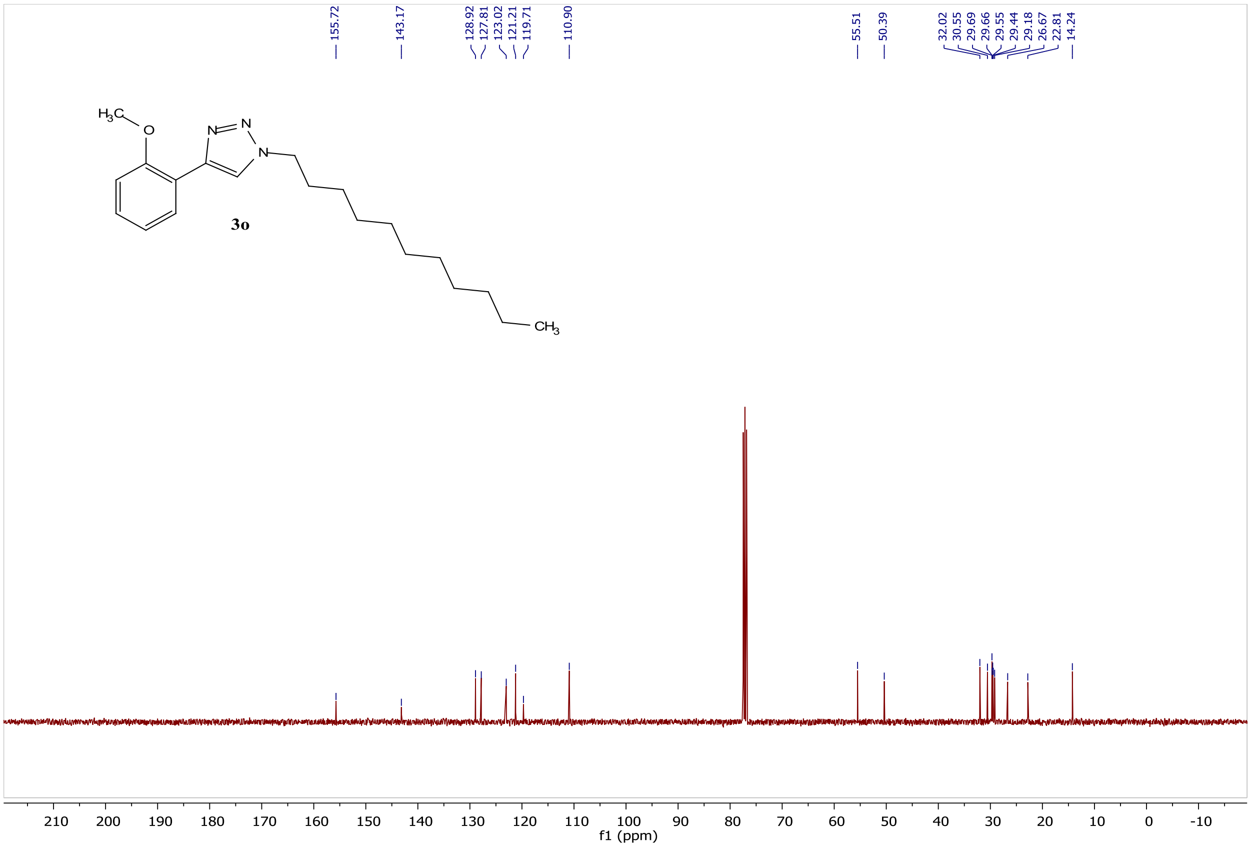
155.72
143.17
128.92
127.81
123.02
121.21
119.71
110.90
55.51
50.39
32.02
30.55
29.69
29.66
29.55
29.44
29.18
26.67
22.81
14.24
H_3C
O
N
N
N
3o
CH_3
f1 (ppm)

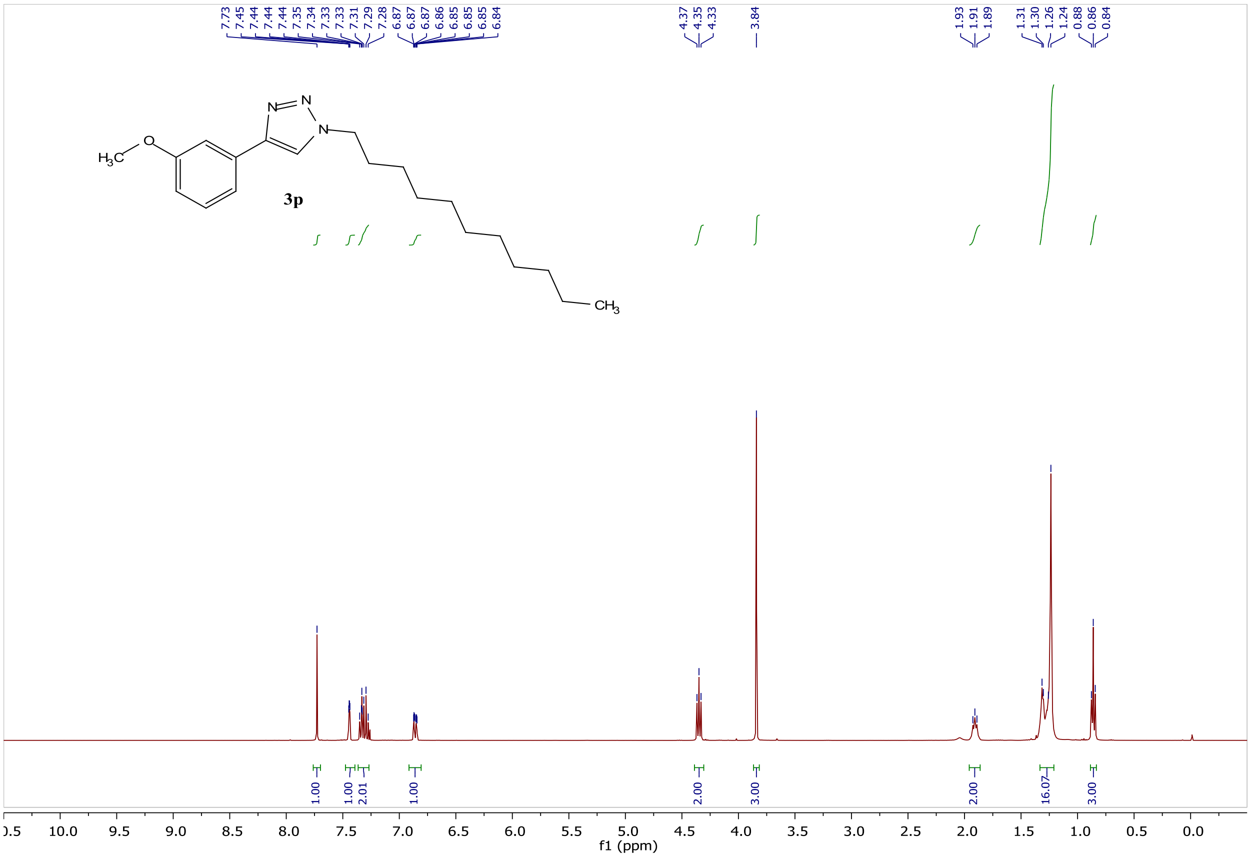
7.73
7.45
7.44
7.44
7.44
7.35
7.34
7.33
7.33
7.31
7.29
7.28
6.87
6.87
6.87
6.86
6.85
6.85
6.84
4.37
4.35
4.33
3.84
1.93
1.91
1.89
1.31
1.30
1.26
1.24
0.88
0.86
0.84
H3C
O
N
N
N
3p
CH3
1.00
1.00
2.01
1.00
2.00
3.00
2.00
16.07
3.00
f1 (ppm)

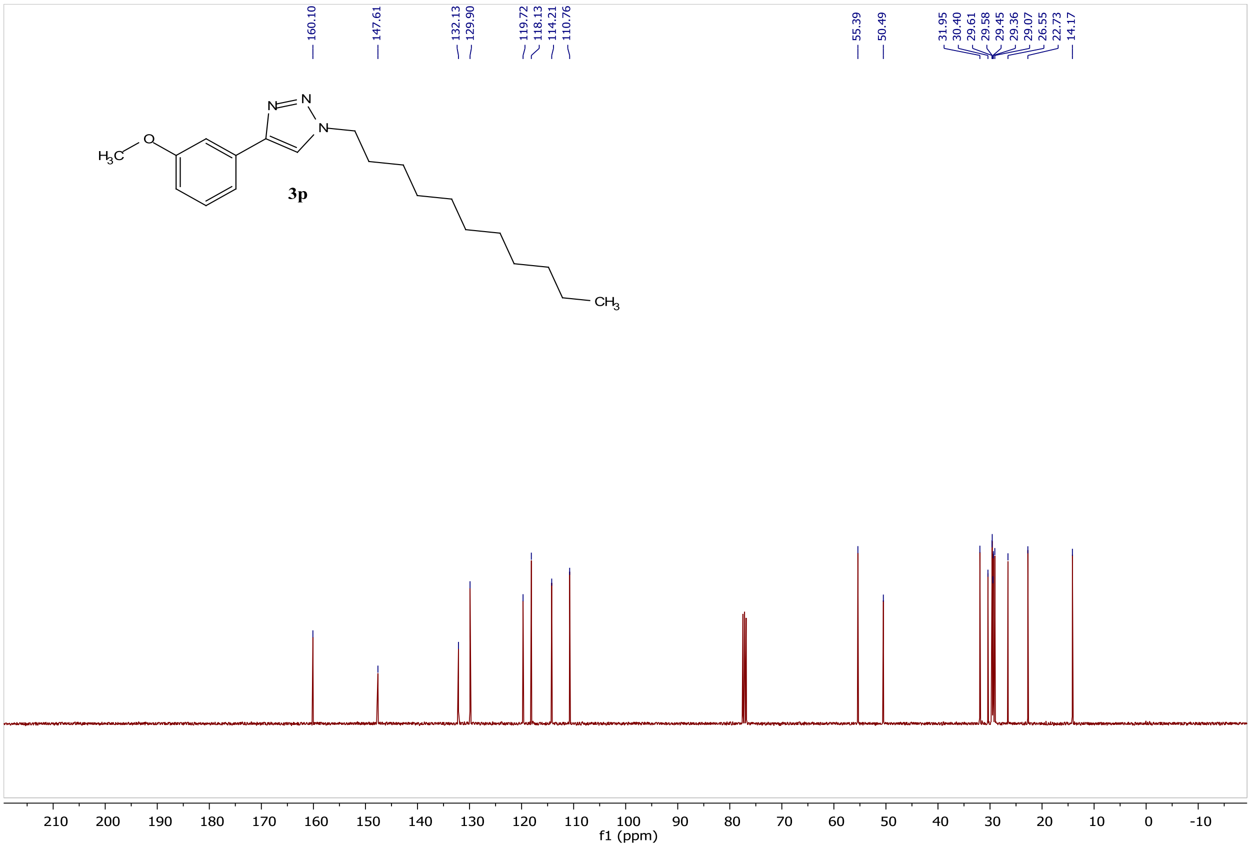
160.10
147.61
132.13
129.90
119.72
118.13
114.21
110.76
55.39
50.49
31.95
30.40
29.61
29.58
29.45
29.36
29.07
26.55
22.73
14.17
H3C
O
N
N
N
3p
CH3
f1 (ppm)

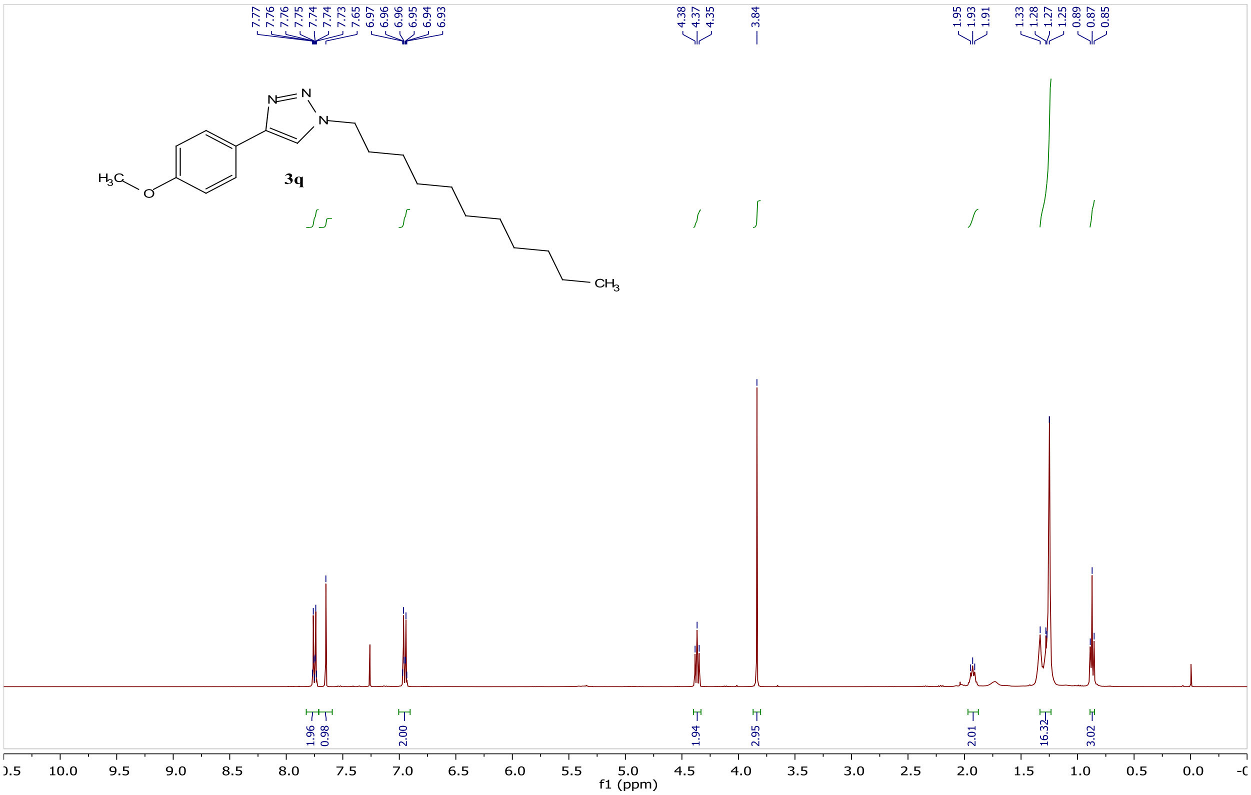
7.77
7.76
7.76
7.75
7.74
7.74
7.73
7.65
6.97
6.96
6.96
6.95
6.94
6.93
4.38
4.37
4.35
3.84
1.95
1.93
1.91
1.33
1.28
1.27
1.25
0.89
0.87
0.85
3q
1.96
0.98
2.00
1.94
2.95
2.01
16.32
3.02
f1 (ppm)

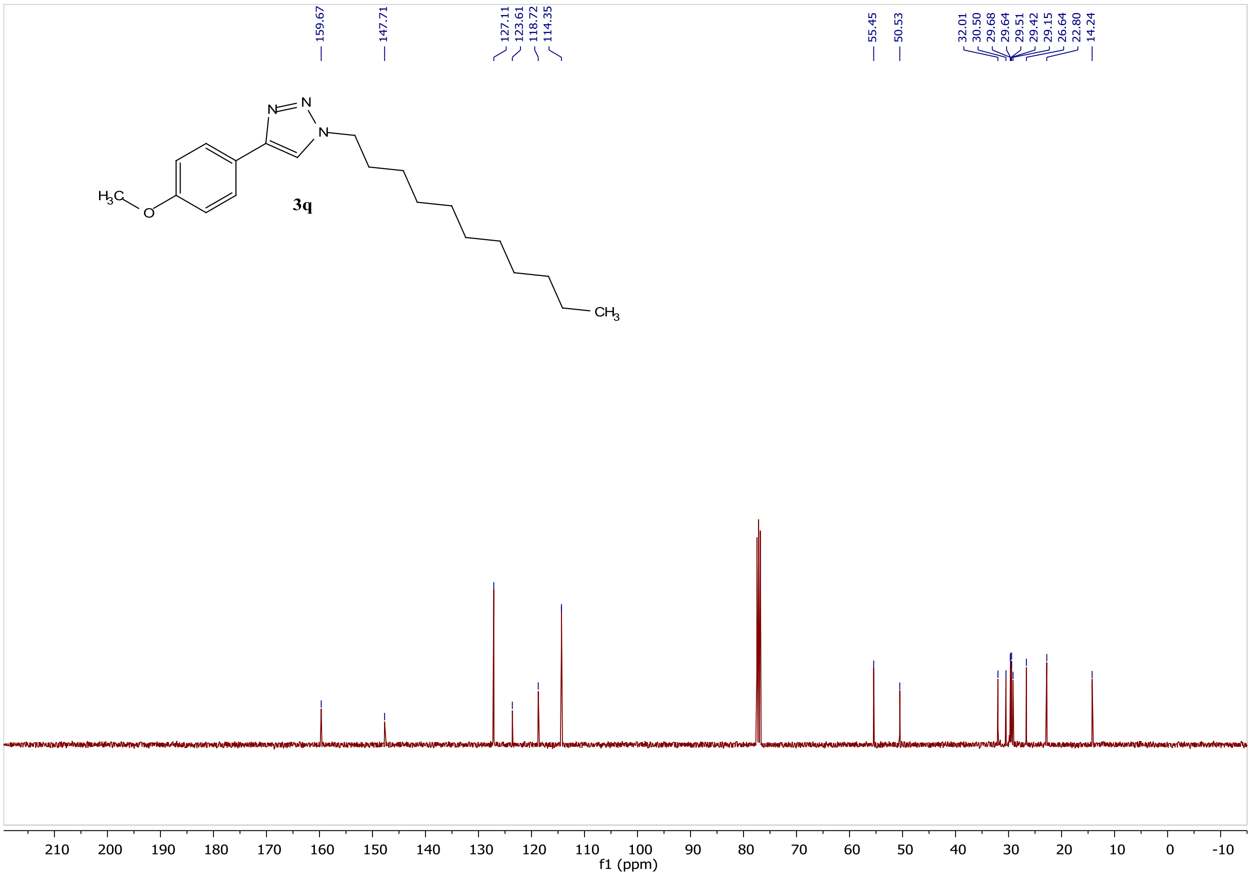
159.67
147.71
127.11
123.61
118.72
114.35
55.45
50.53
32.01
30.50
29.68
29.64
29.51
29.42
29.15
26.64
22.80
14.24
3q
f1 (ppm)

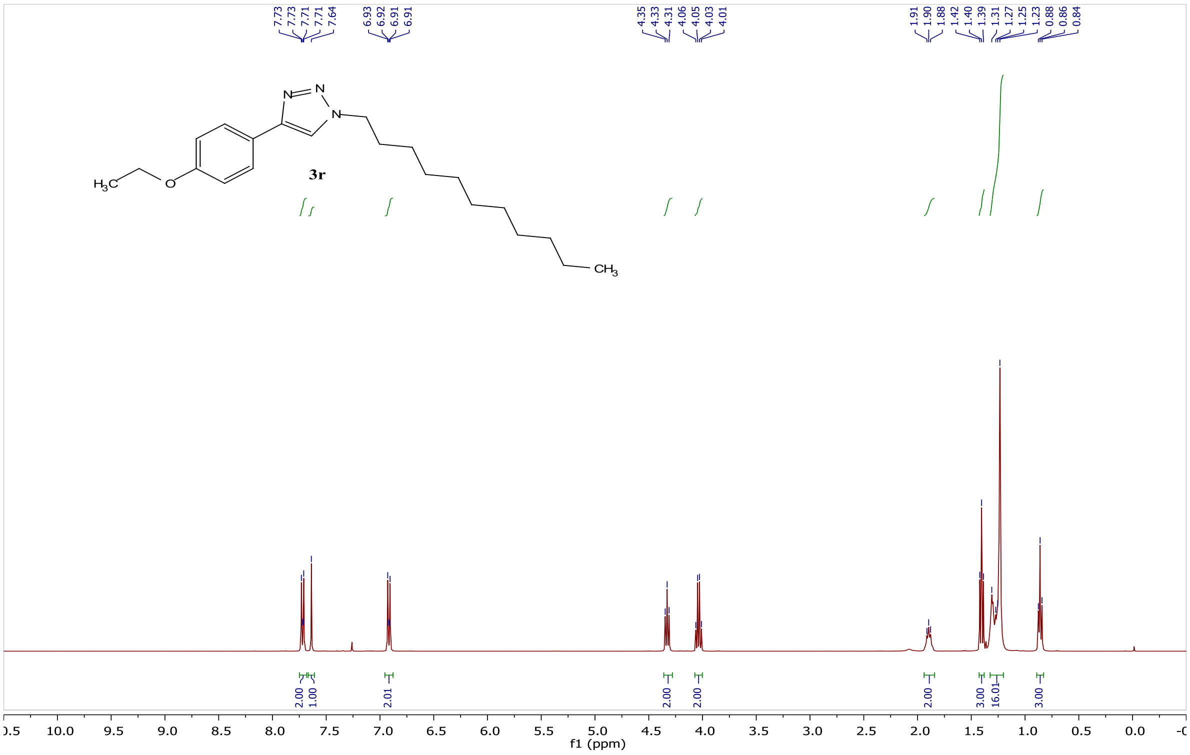

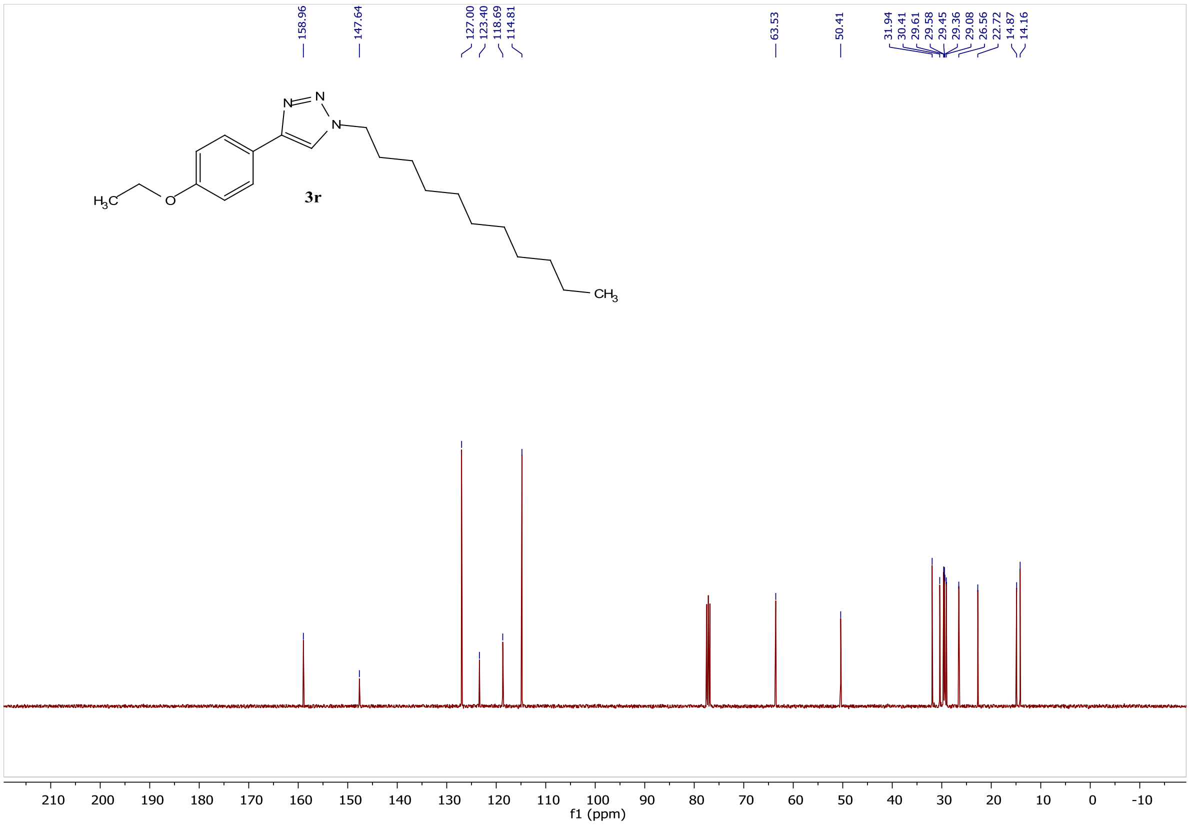

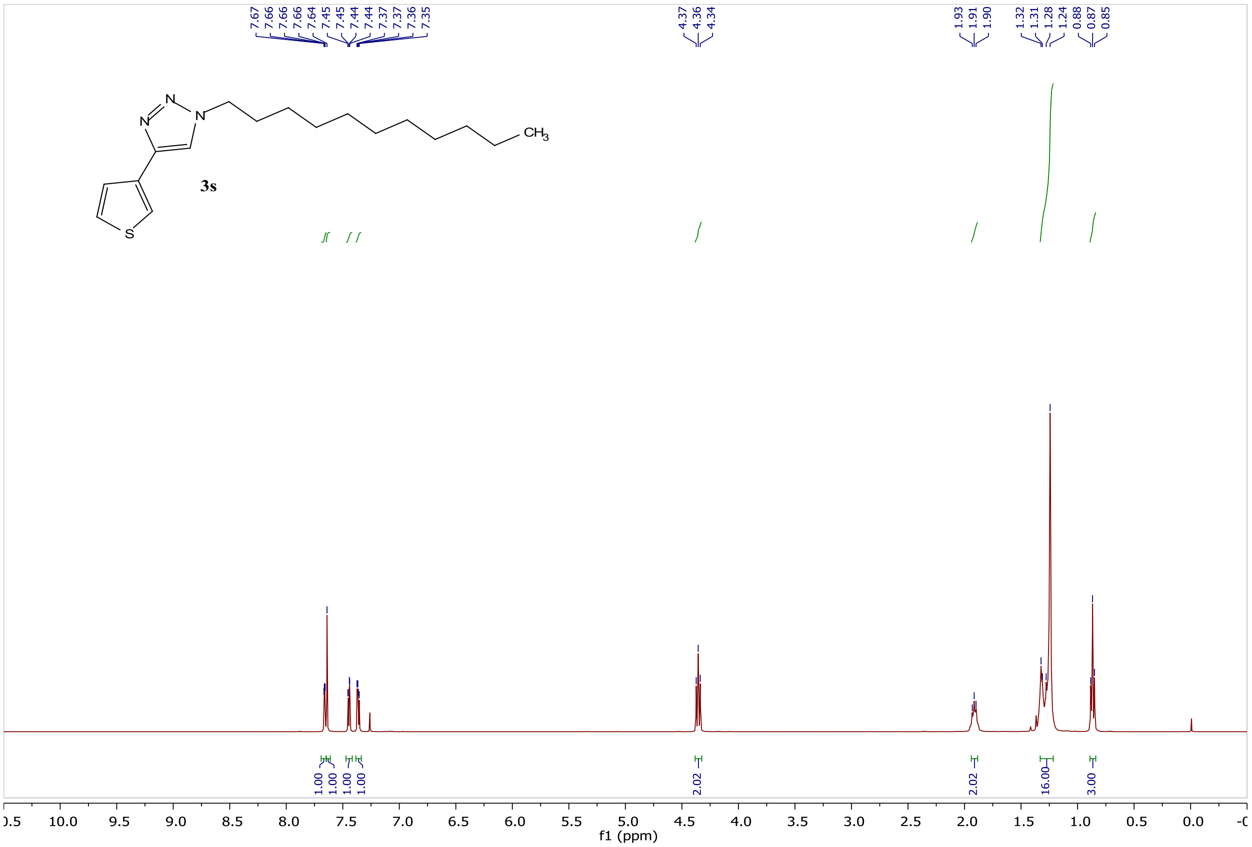

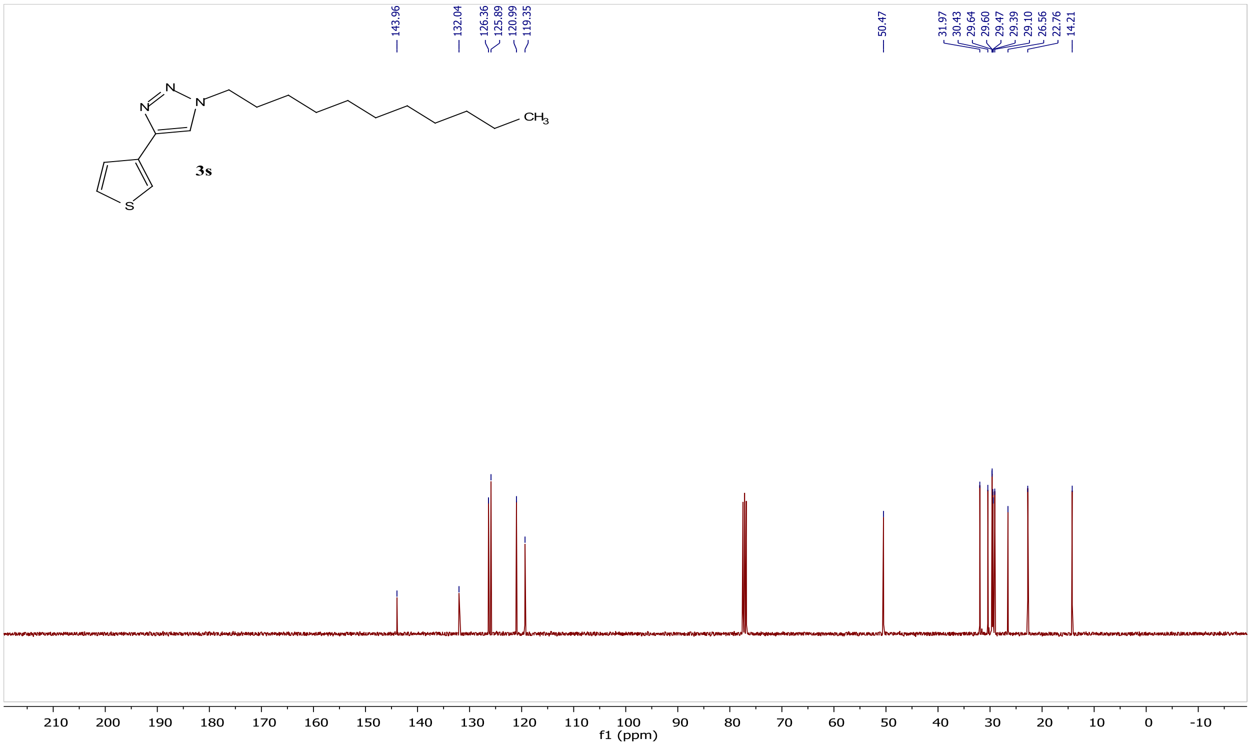

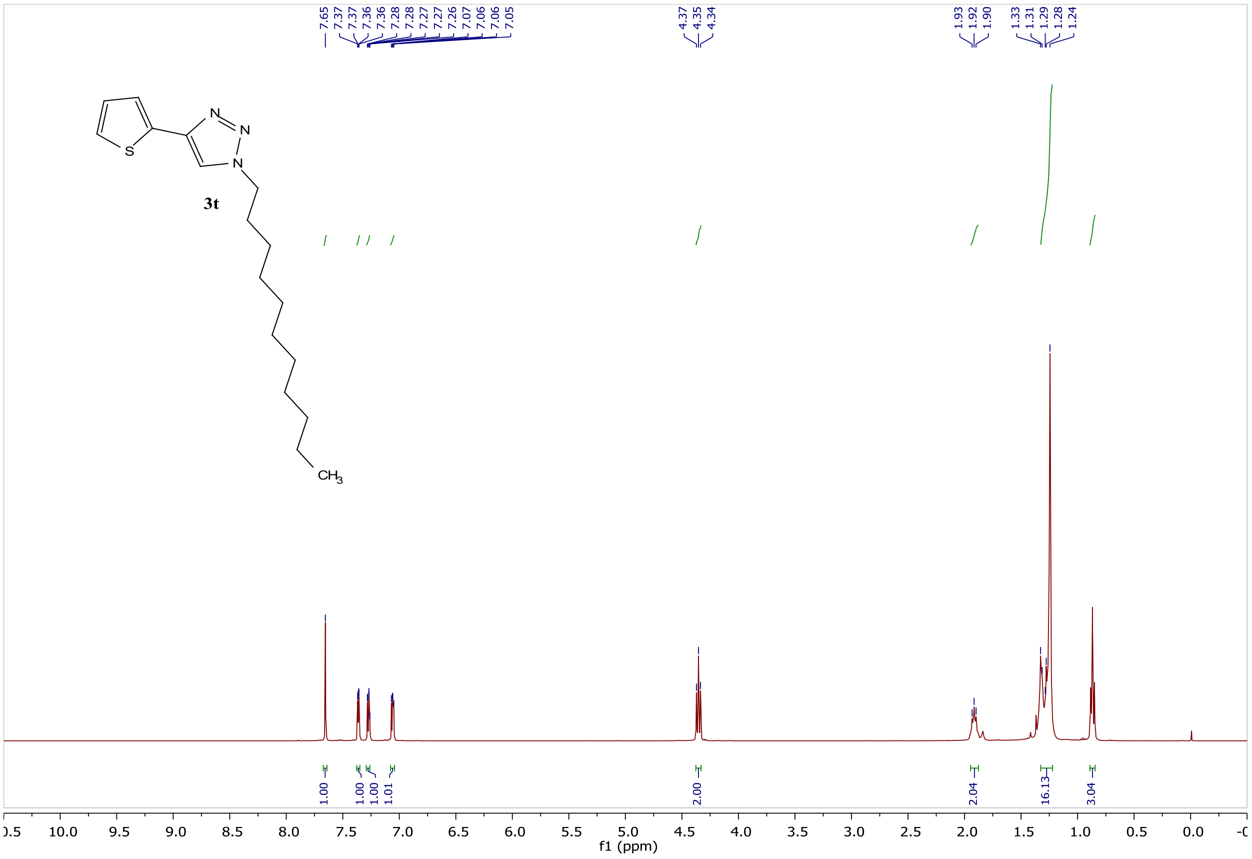
7.65
7.37
7.37
7.36
7.36
7.28
7.28
7.27
7.26
7.07
7.06
7.06
7.05
4.37
4.35
4.34
1.93
1.92
1.90
1.33
1.31
1.29
1.28
1.24
N
N
N
S
3t
CH_3
1.00
1.00
1.00
1.01
2.00
2.04
16.13
3.04
f1 (ppm)

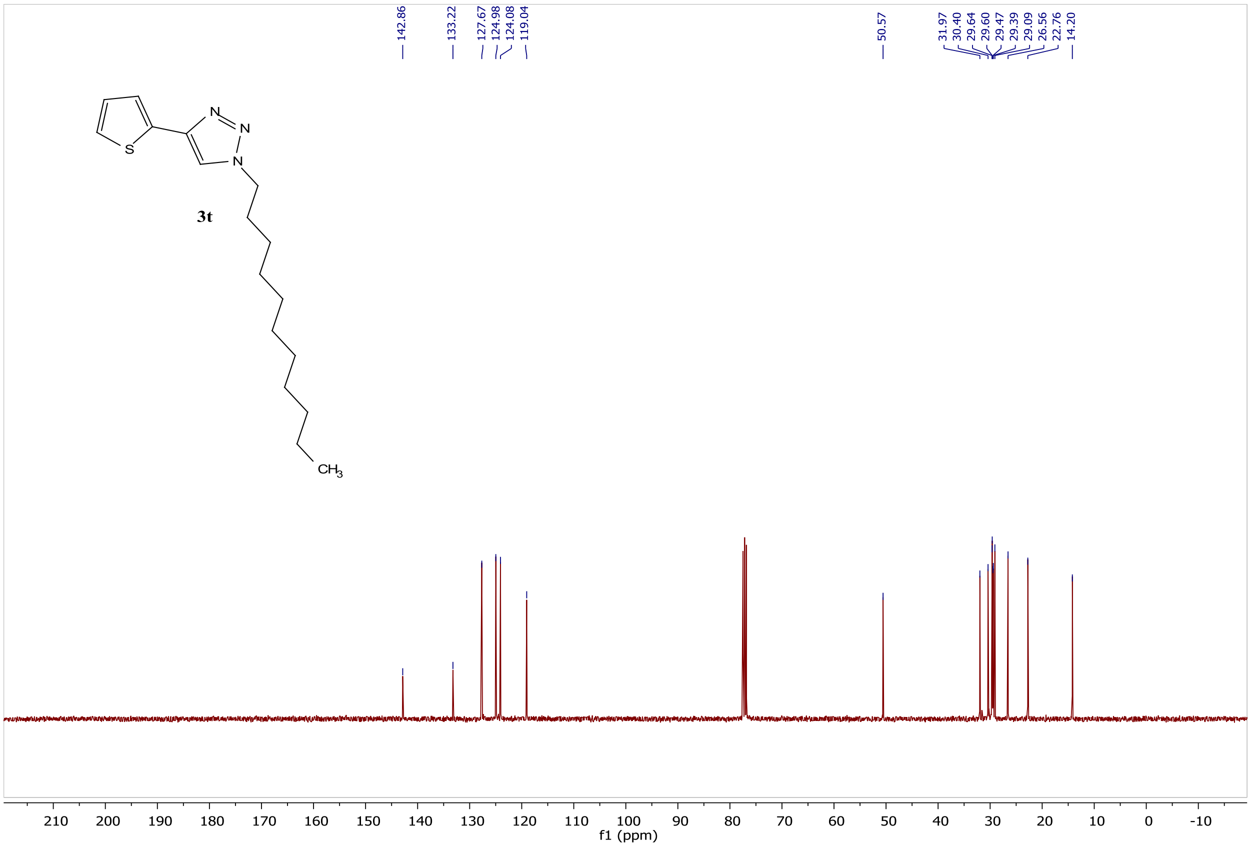
142.86
133.22
127.67
124.98
124.08
119.04
50.57
31.97
30.40
29.64
29.60
29.47
29.39
29.09
26.56
22.76
14.20
N
N
N
S
3t
CH_3
f1 (ppm)

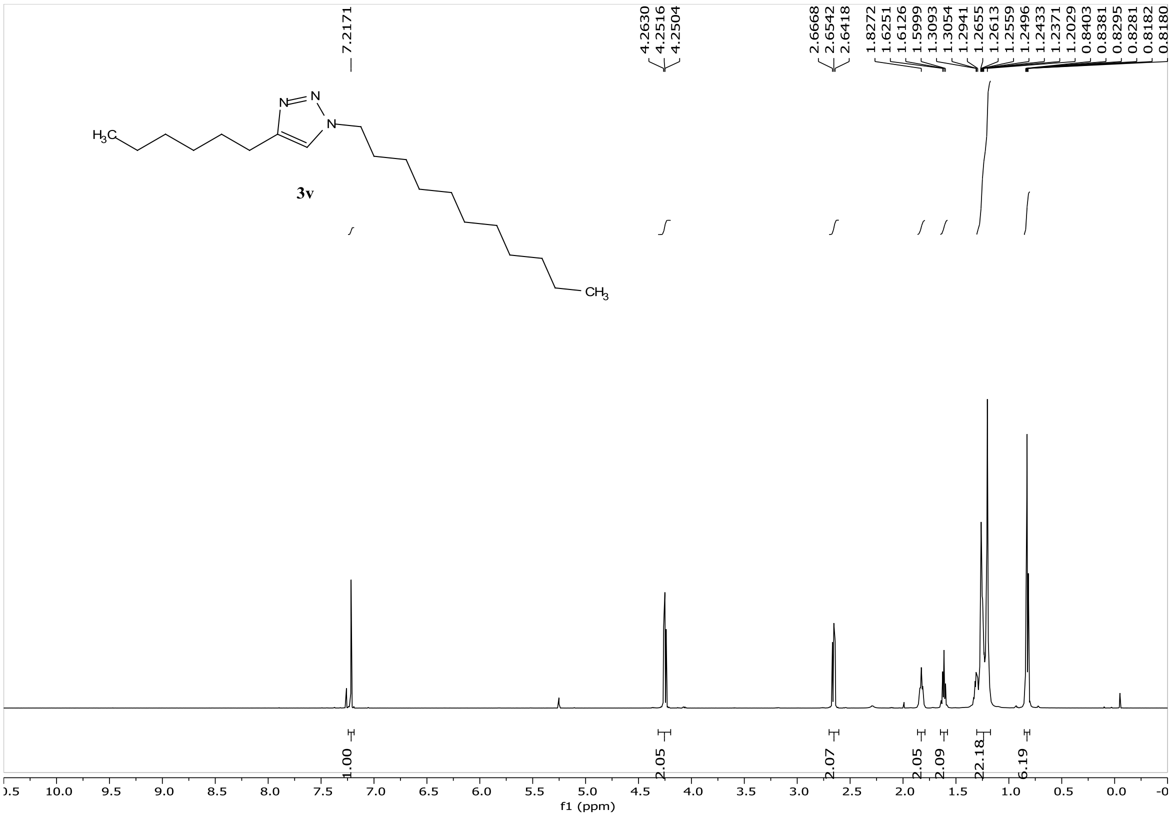
7.2171
4.2630
4.2516
4.2504
2.6668
2.6542
2.6418
1.8272
1.6251
1.6126
1.5999
1.3093
1.3054
1.2941
1.2655
1.2613
1.2559
1.2496
1.2433
1.2371
1.2029
0.8403
0.8381
0.8295
0.8281
0.8182
0.8180
H3C
N
N
N
CH3
3v
1.00
2.05
2.07
2.05
2.09
22.18
6.19
f1 (ppm)

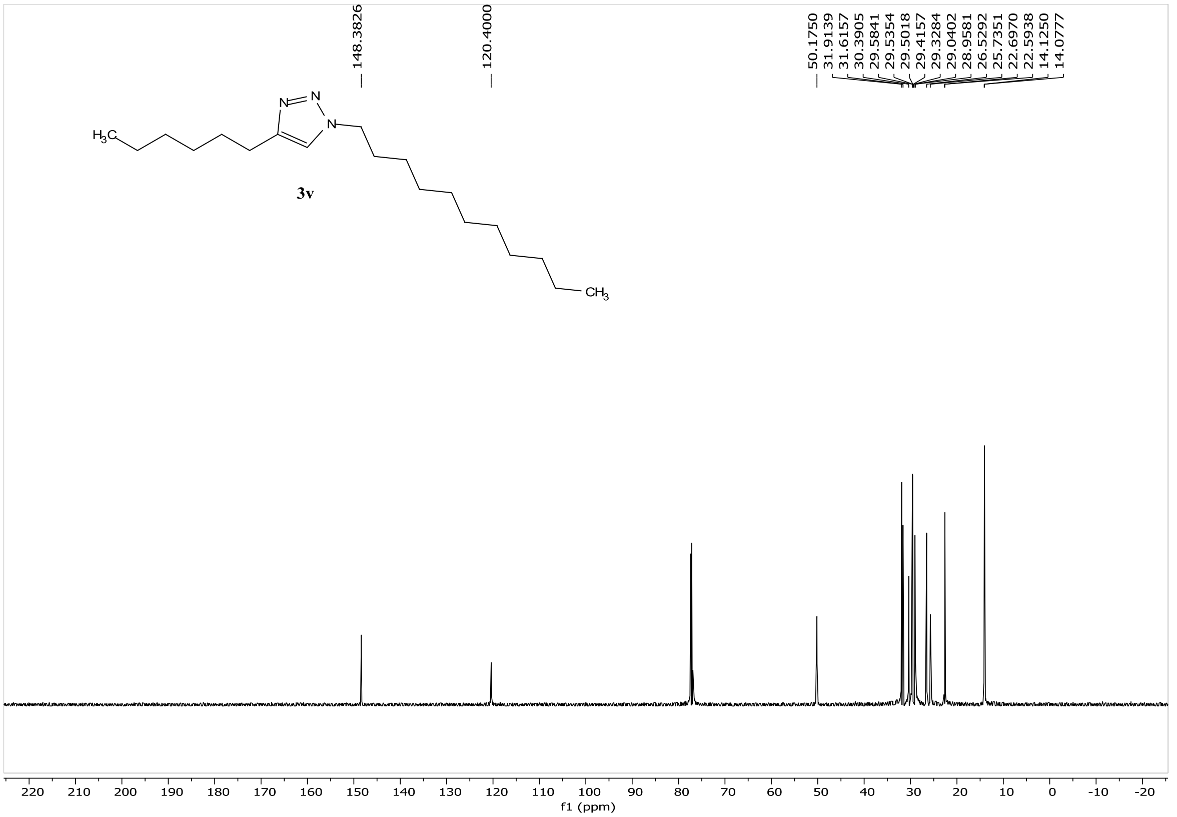
148.3826
120.4000
50.1750
31.9139
31.6157
30.3905
29.5841
29.5354
29.5018
29.4157
29.3284
29.0402
28.9581
26.5292
25.7351
22.6970
22.5938
14.1250
14.0777
H3C
N
N
N
CH3
3v
f1 (ppm)

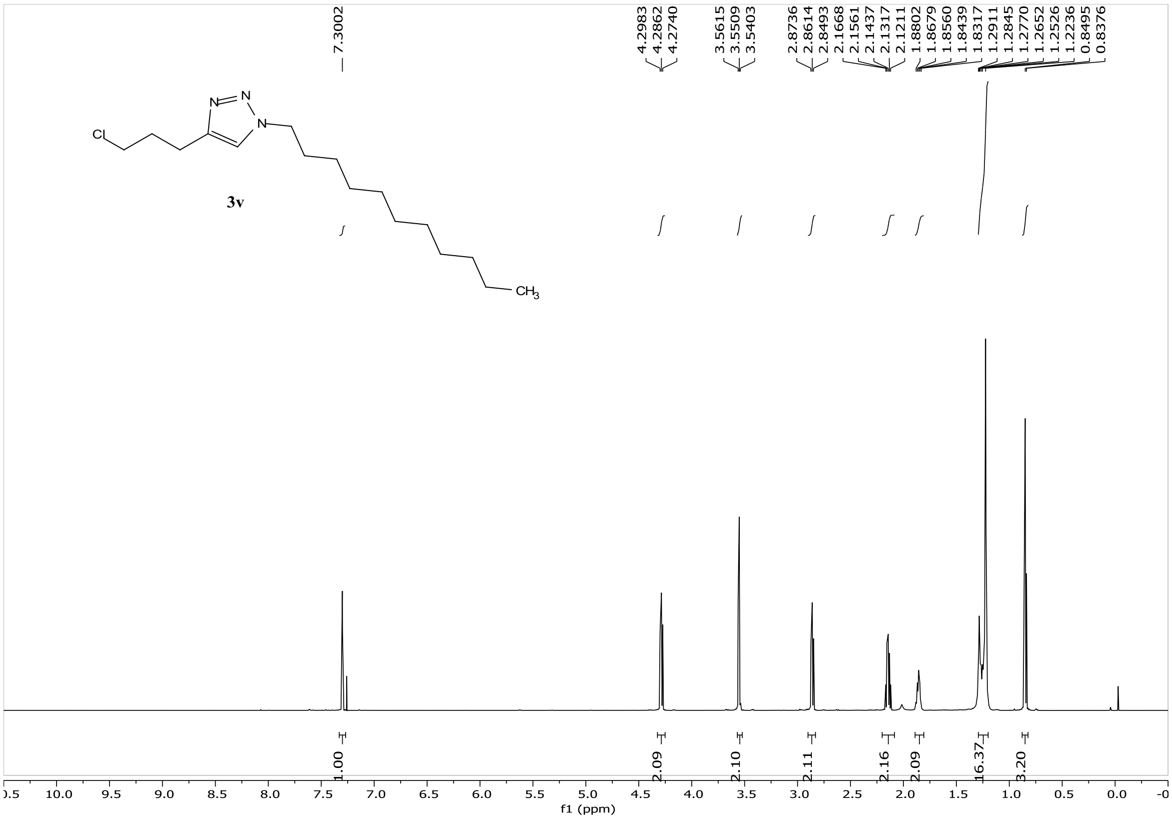
3v
f1 (ppm)

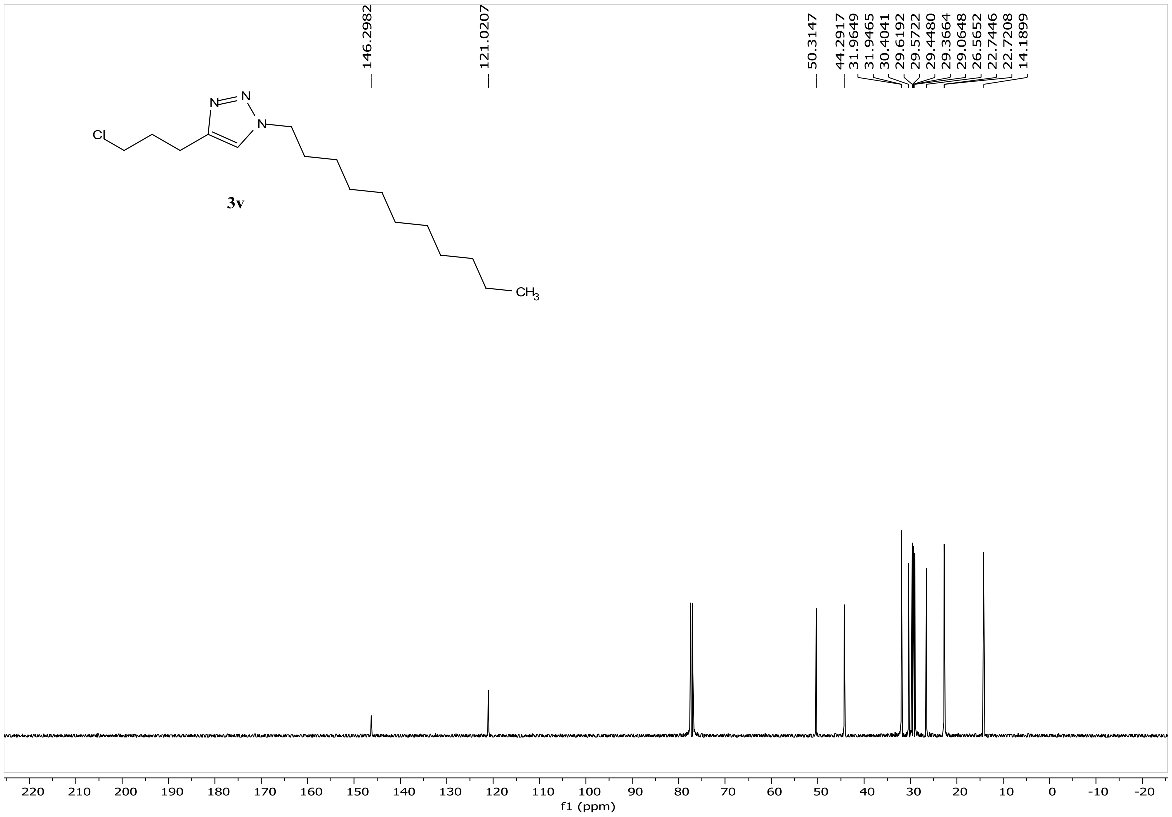
3v
f1 (ppm)

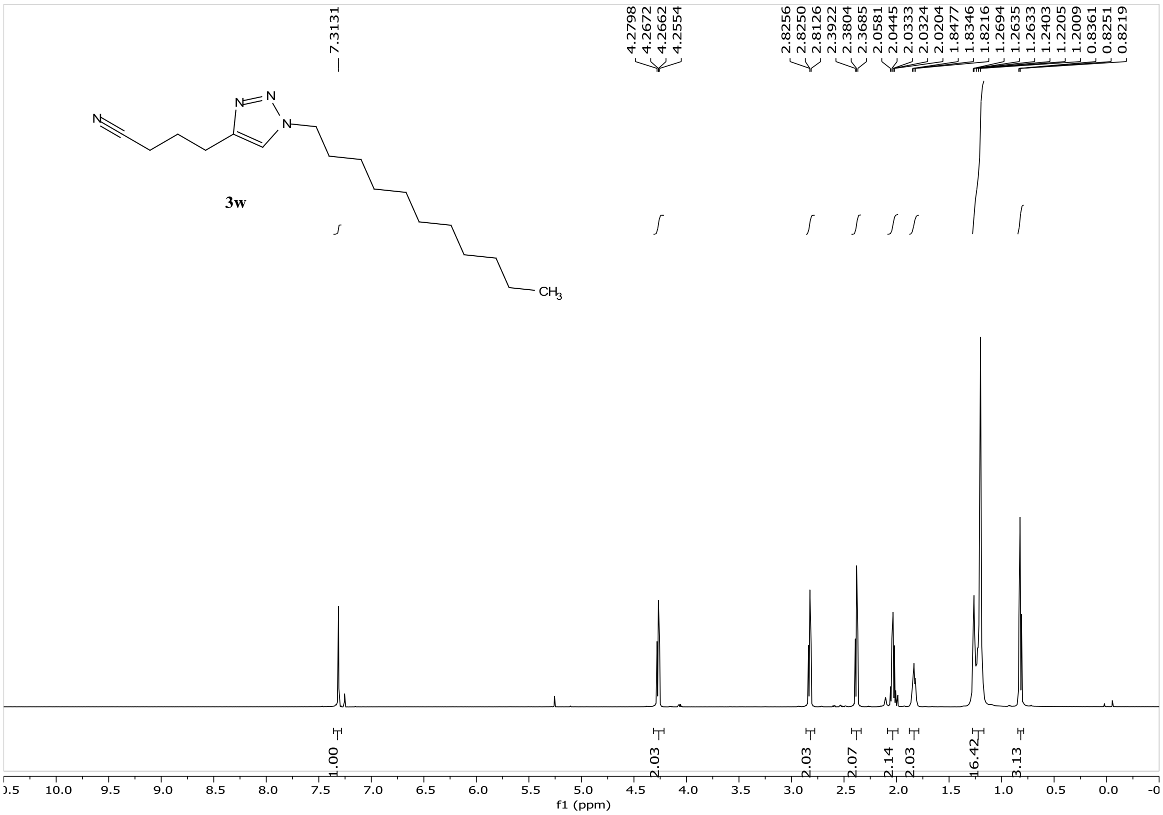
3w
7.3131
4.2798
4.2672
4.2662
4.2554
2.8256
2.8250
2.8126
2.3922
2.3804
2.3685
2.0581
2.0445
2.0333
2.0324
2.0204
1.8477
1.8346
1.8216
1.2694
1.2635
1.2633
1.2403
1.2205
1.2009
0.8361
0.8251
0.8219
1.00
2.03
2.03
2.07
2.14
2.03
16.42
3.13
f1 (ppm)

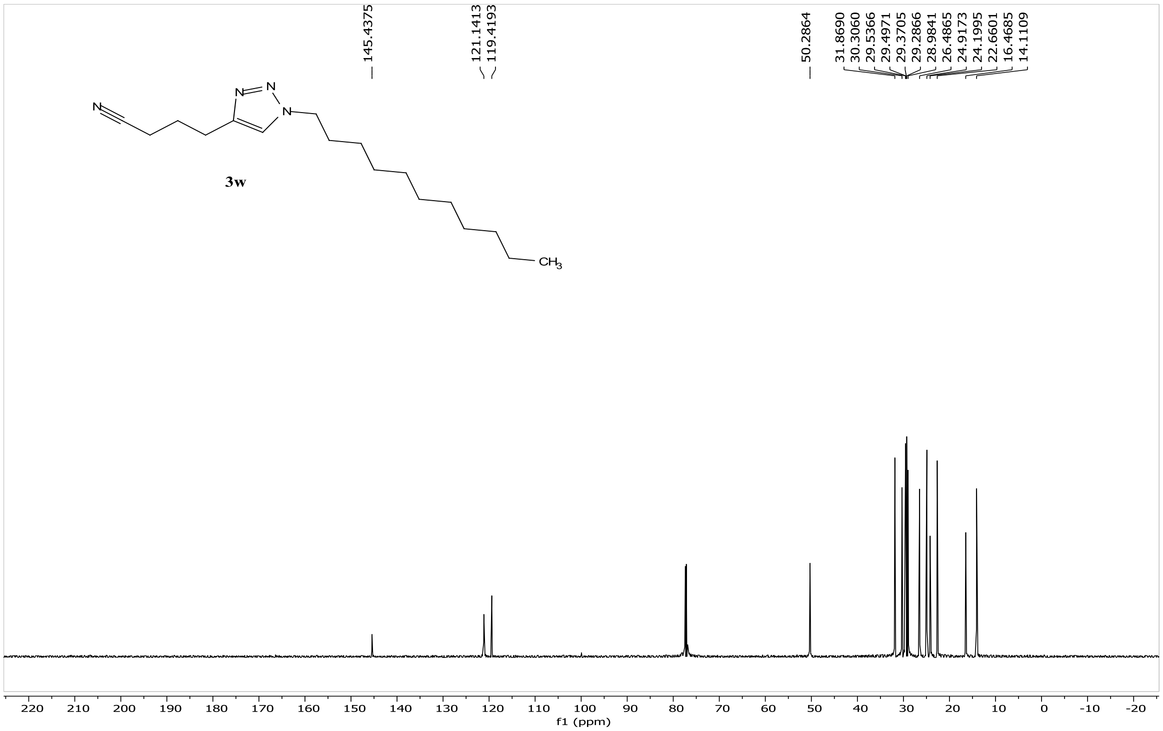
3w
145.4375
121.1413
119.4193
50.2864
31.8690
30.3060
29.5366
29.4971
29.3705
29.2866
28.9841
26.4865
24.9173
24.1995
22.6601
16.4685
14.1109
f1 (ppm)

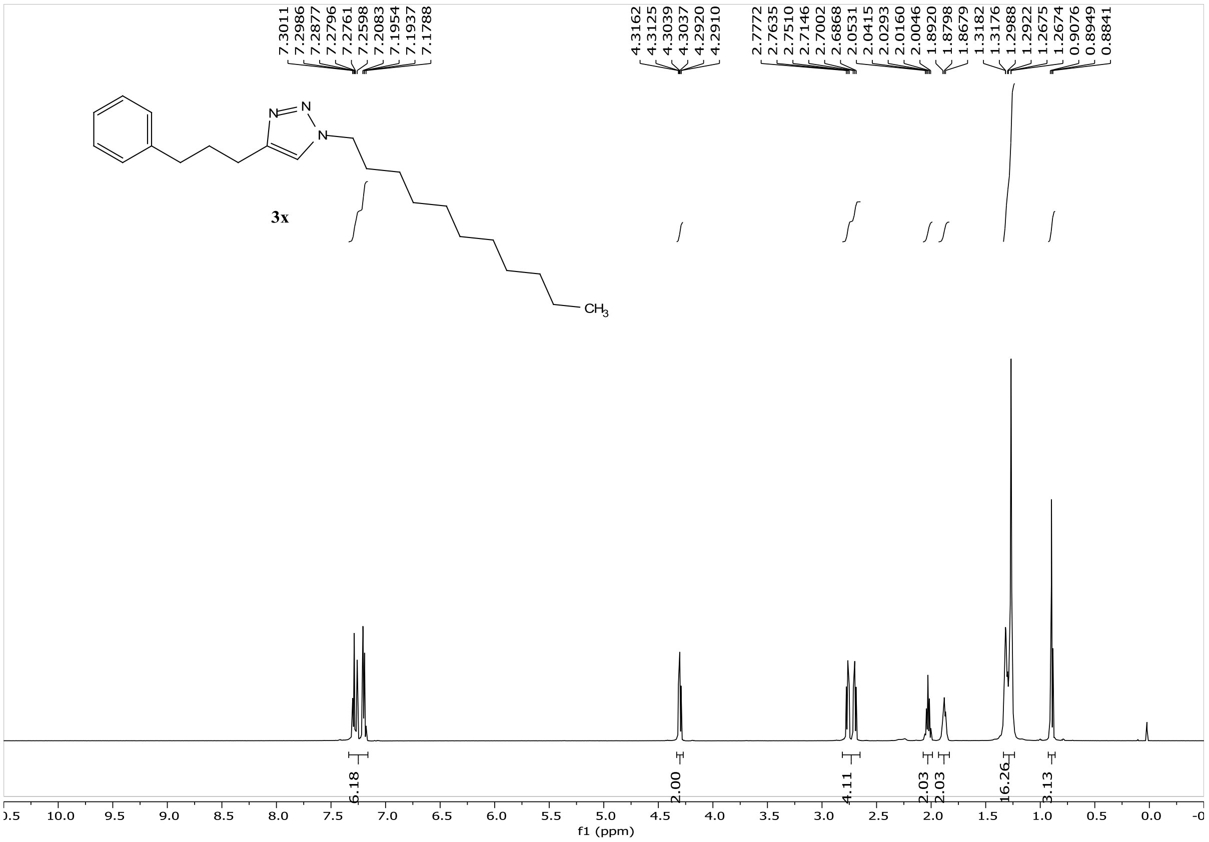
7.3011
7.2986
7.2877
7.2796
7.2761
7.2598
7.2083
7.1954
7.1937
7.1788
4.3162
4.3125
4.3039
4.3037
4.2920
4.2910
2.7772
2.7635
2.7510
2.7146
2.7002
2.6868
2.0531
2.0415
2.0293
2.0160
2.0046
1.8920
1.8798
1.8679
1.3182
1.3176
1.2988
1.2922
1.2675
1.2674
0.9076
0.8949
0.8841
3x
CH3
6.18
2.00
4.11
2.03
2.03
16.26
3.13
f1 (ppm)

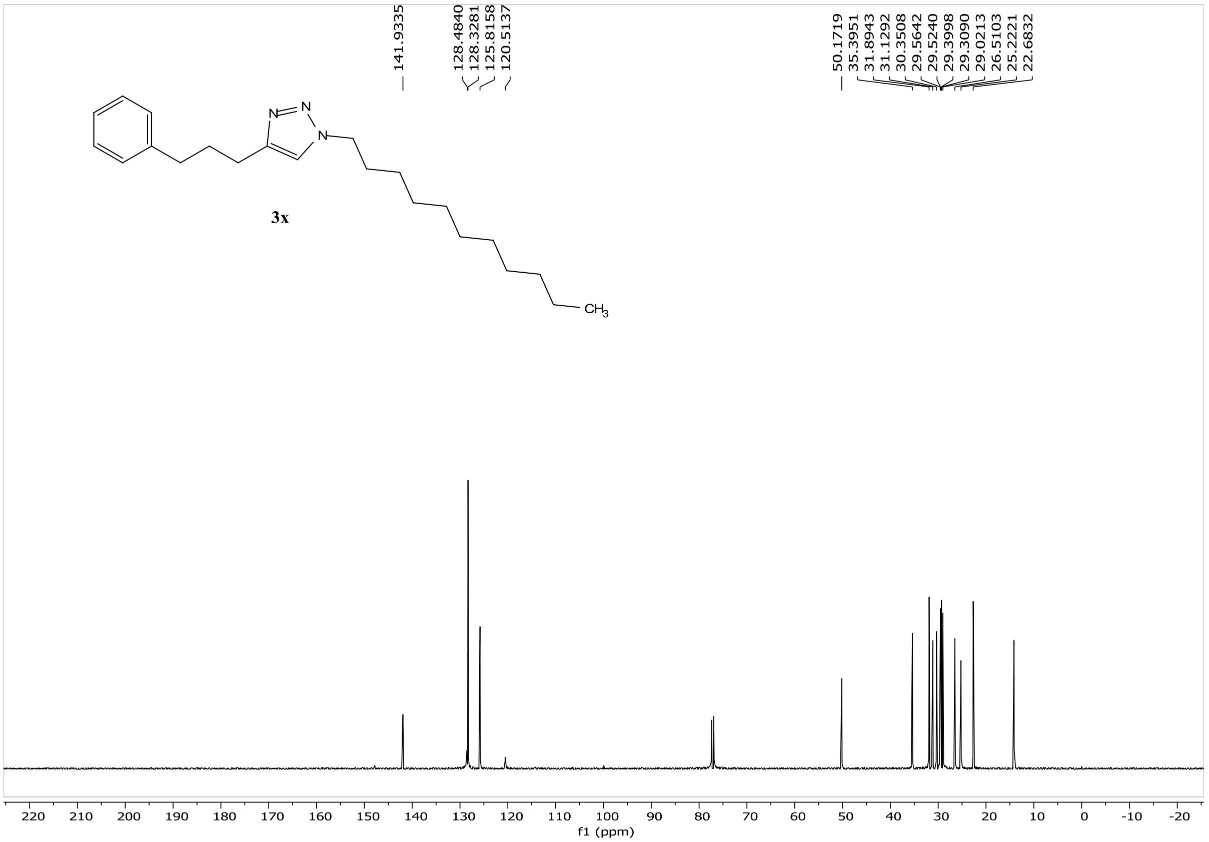
141.9335
128.4840
128.3281
125.8158
120.5137
50.1719
35.3951
31.8943
31.1292
30.3508
29.5642
29.5240
29.3998
29.3090
29.0213
26.5103
25.2221
22.6832
3x
CH3
f1 (ppm)

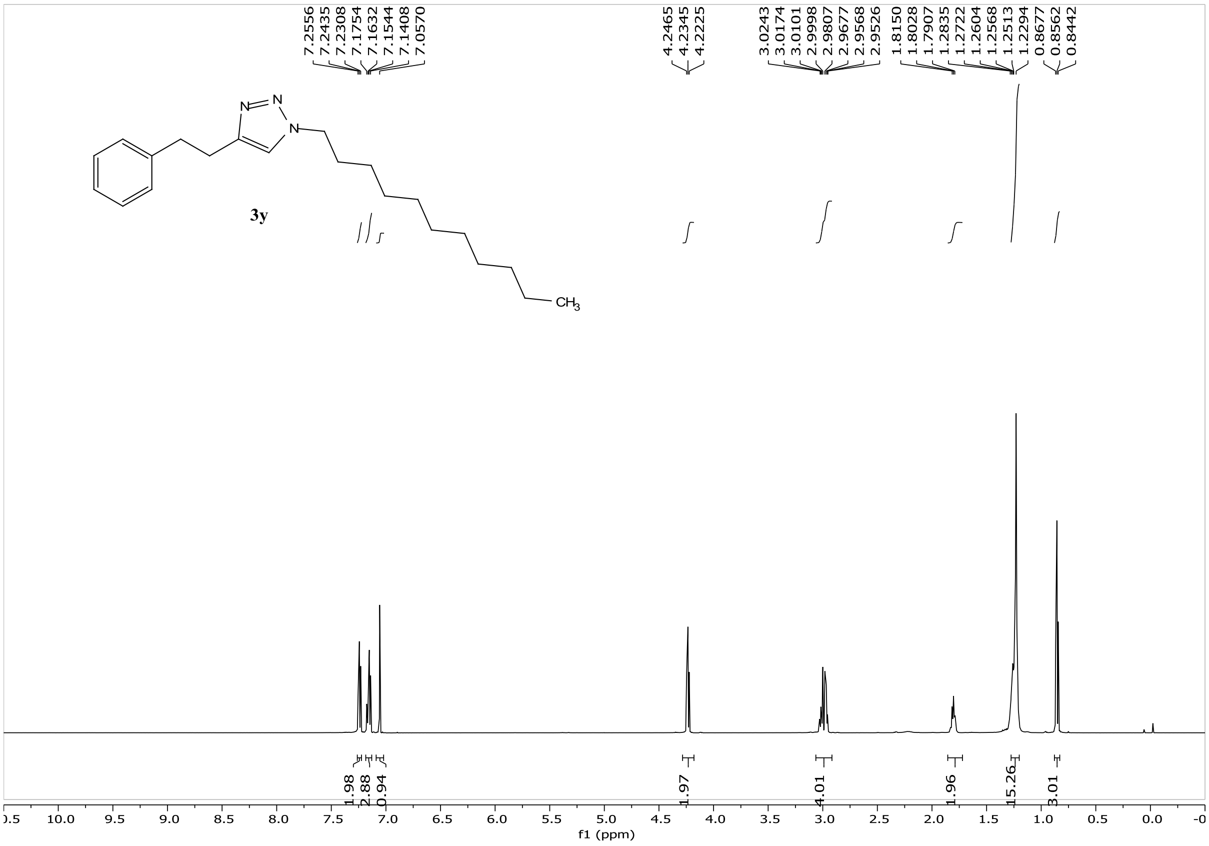
7.2556
7.2435
7.2308
7.1754
7.1632
7.1544
7.1408
7.0570
4.2465
4.2345
4.2225
3.0243
3.0174
3.0101
2.9998
2.9807
2.9677
2.9568
2.9526
1.8150
1.8028
1.7907
1.2835
1.2722
1.2604
1.2568
1.2513
1.2294
0.8677
0.8562
0.8442
3y
CH3
1.98
2.88
0.94
1.97
4.01
1.96
15.26
3.01
f1 (ppm)

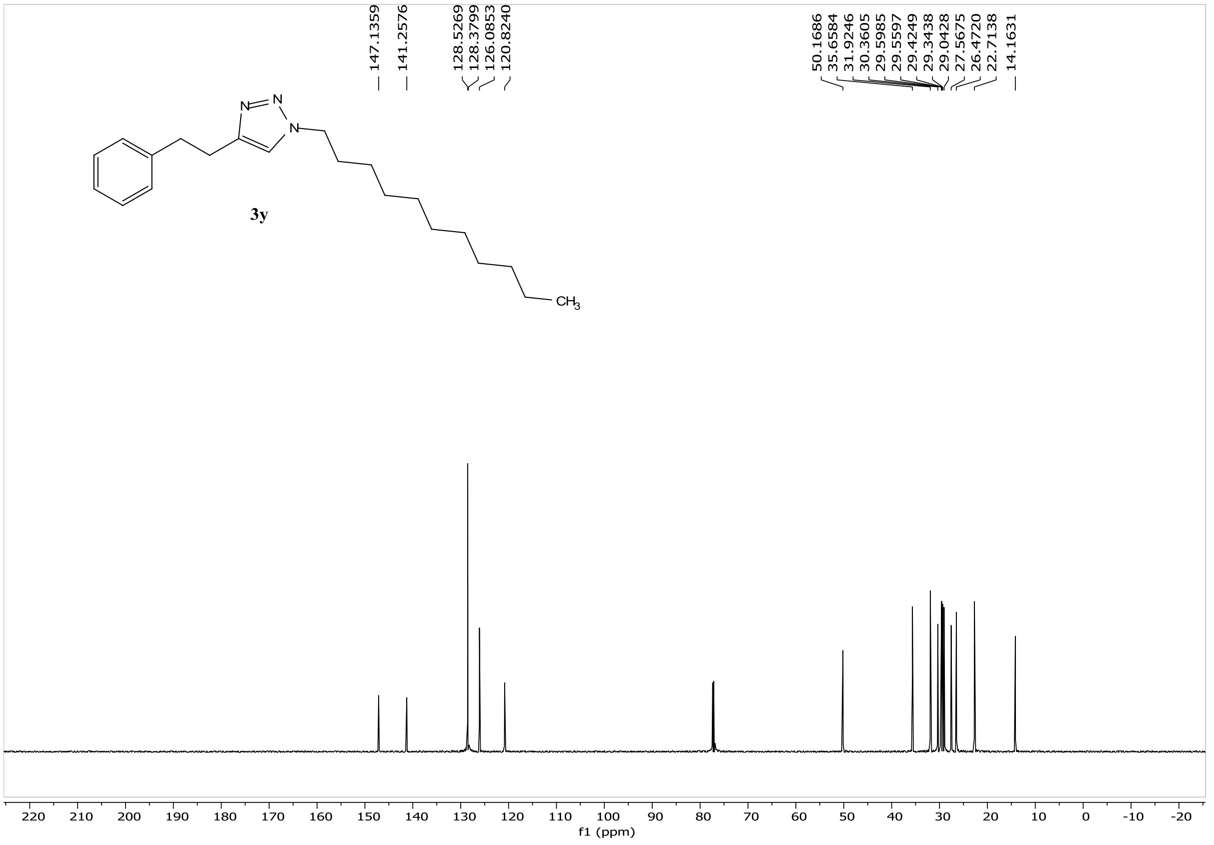
147.1359
141.2576
128.5269
128.3799
126.0853
120.8240
50.1686
35.6584
31.9246
30.3605
29.5985
29.5597
29.4249
29.3438
29.0428
27.5675
26.4720
22.7138
14.1631
3y
CH3
f1 (ppm)

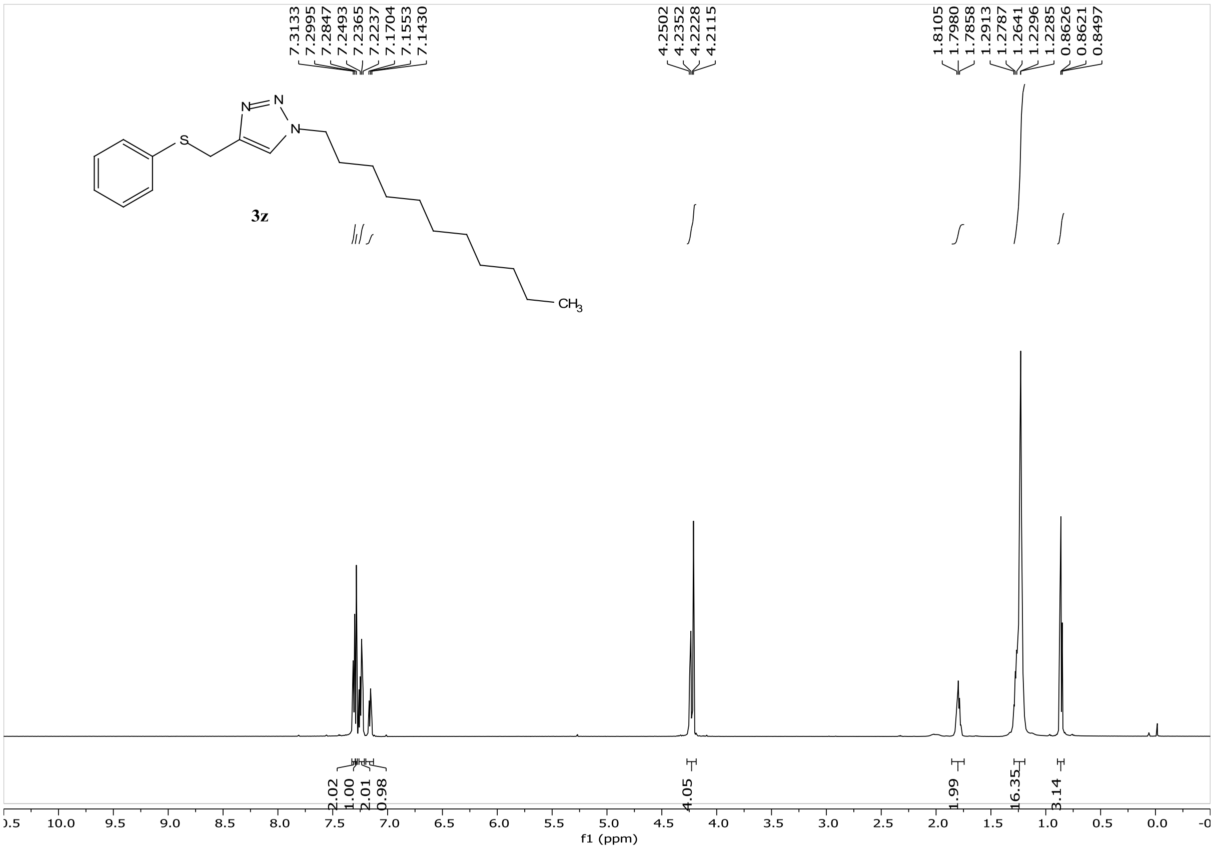
7.3133
7.2995
7.2847
7.2493
7.2365
7.2237
7.1704
7.1553
7.1430
4.2502
4.2352
4.2228
4.2115
1.8105
1.7980
1.7858
1.2913
1.2787
1.2641
1.2296
1.2285
0.8626
0.8621
0.8497
3z
CH3
2.02
1.00
2.01
0.98
4.05
1.99
16.35
3.14
f1 (ppm)

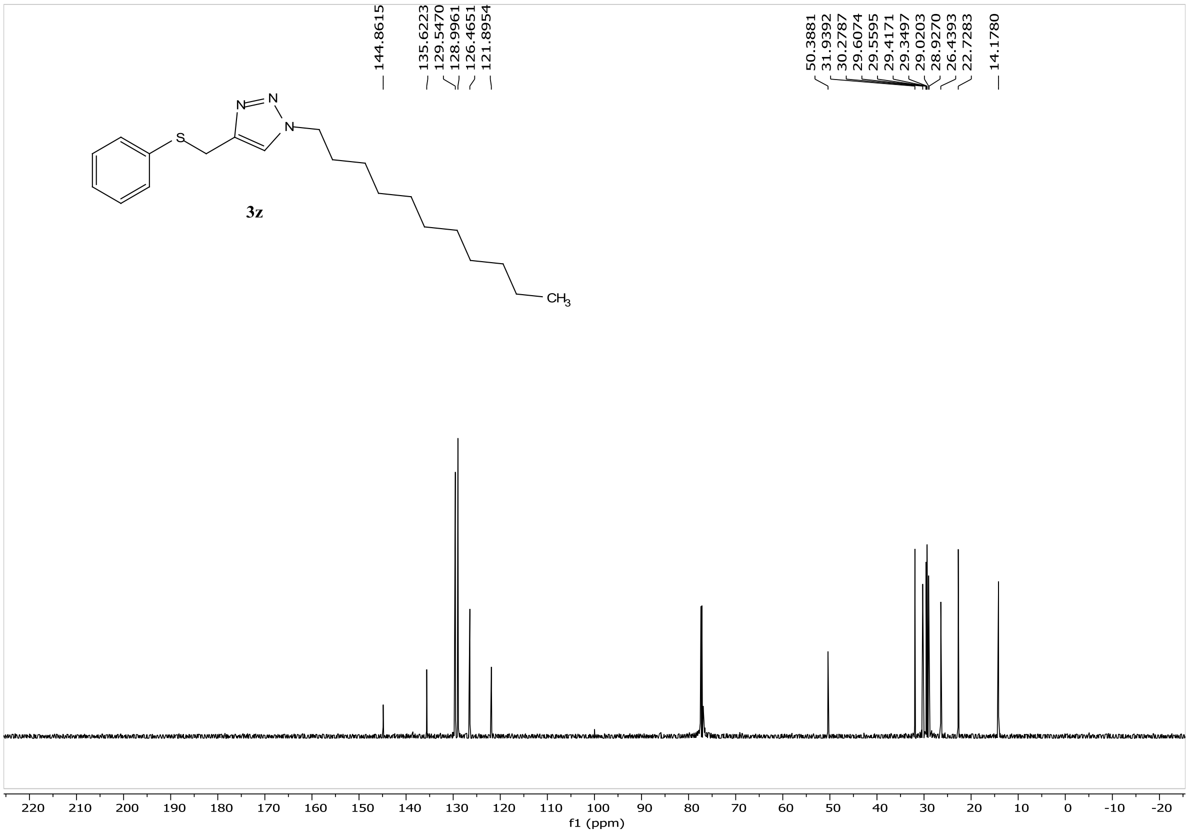
144.8615
135.6223
129.5470
128.9961
126.4651
121.8954
50.3881
31.9392
30.2787
29.6074
29.5595
29.4171
29.3497
29.0203
28.9270
26.4393
22.7283
14.1780
3z
CH3
f1 (ppm)

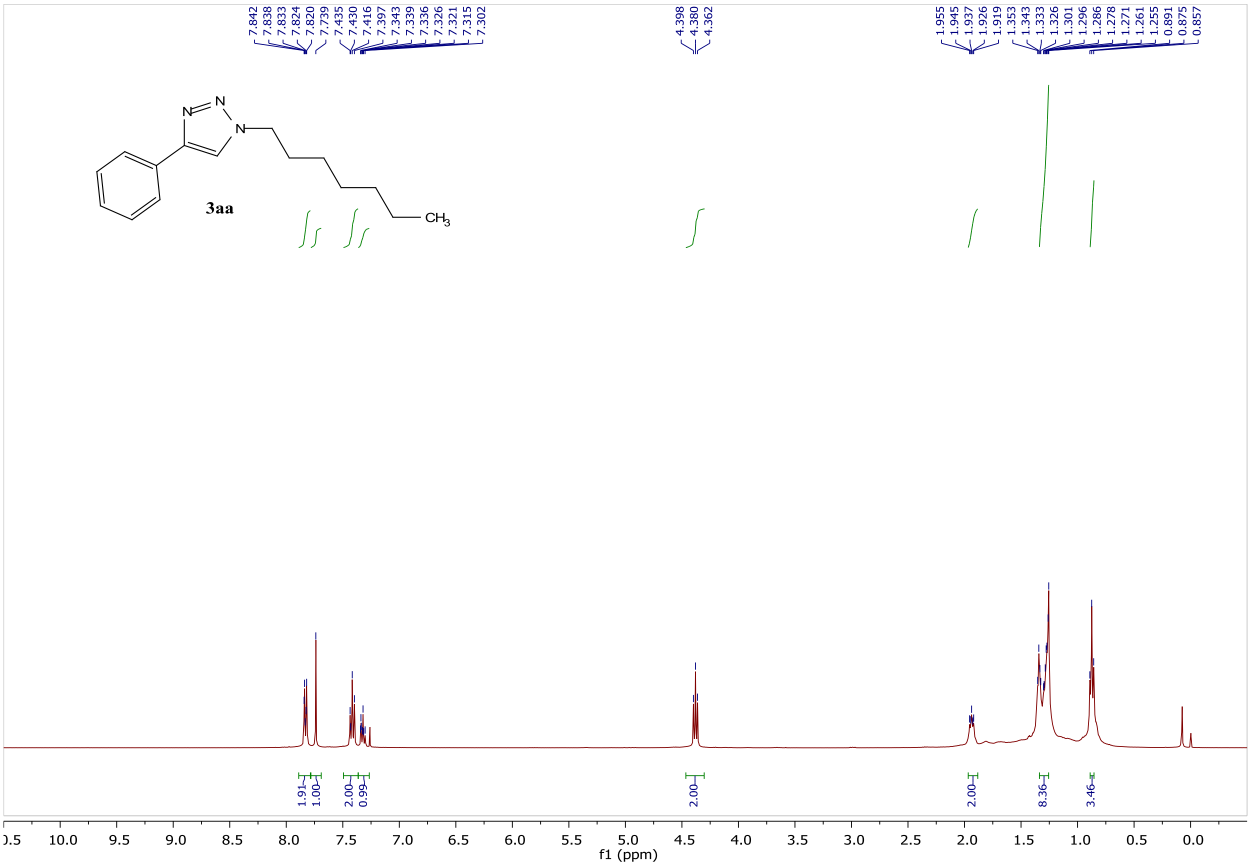
7.842
7.838
7.833
7.824
7.820
7.739
7.435
7.430
7.416
7.397
7.343
7.339
7.336
7.326
7.321
7.315
7.302
4.398
4.380
4.362
1.955
1.945
1.937
1.926
1.919
1.353
1.343
1.333
1.326
1.301
1.296
1.286
1.278
1.271
1.261
1.255
0.891
0.875
0.857
3aa
CH3
1.91
1.00
2.00
0.99
2.00
2.00
8.36
3.46
f1 (ppm)

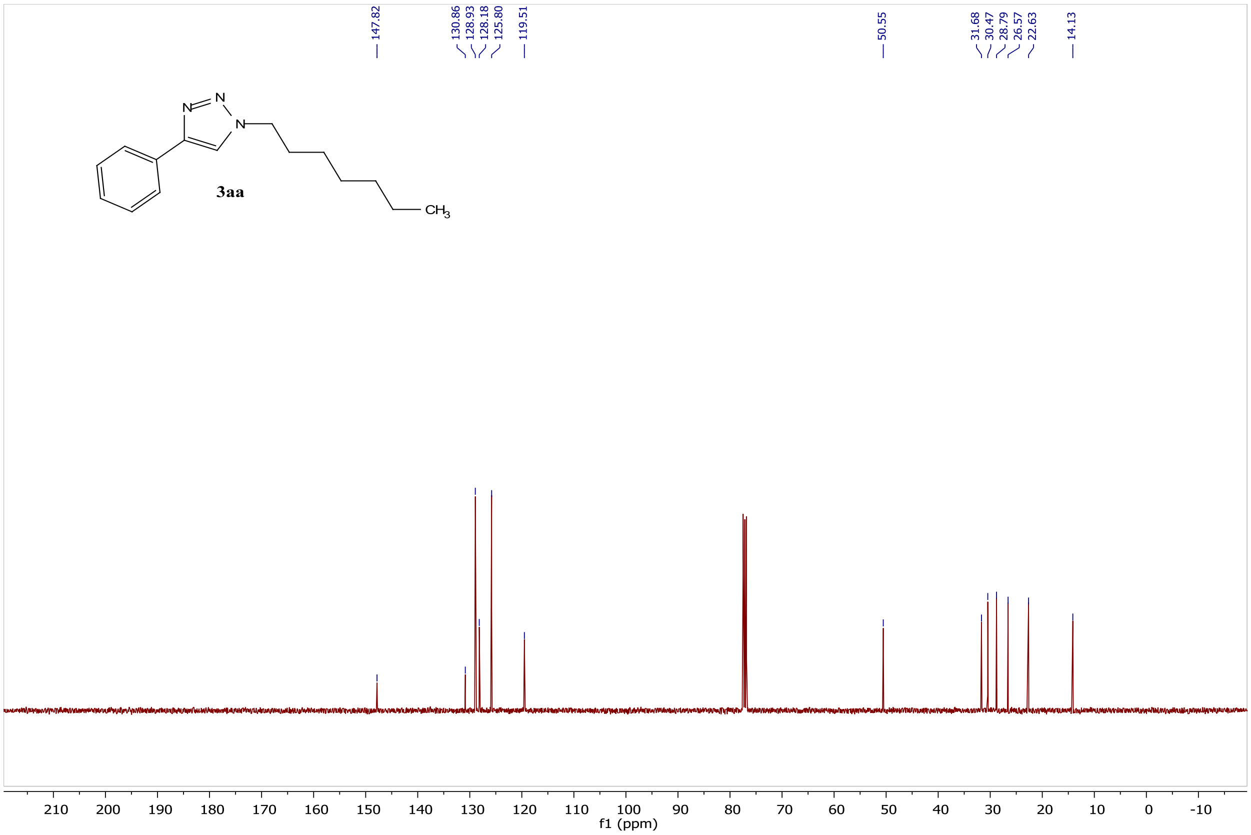
147.82
130.86
128.93
128.18
125.80
119.51
50.55
31.68
30.47
28.79
26.57
22.63
14.13
3aa
CH3
f1 (ppm)

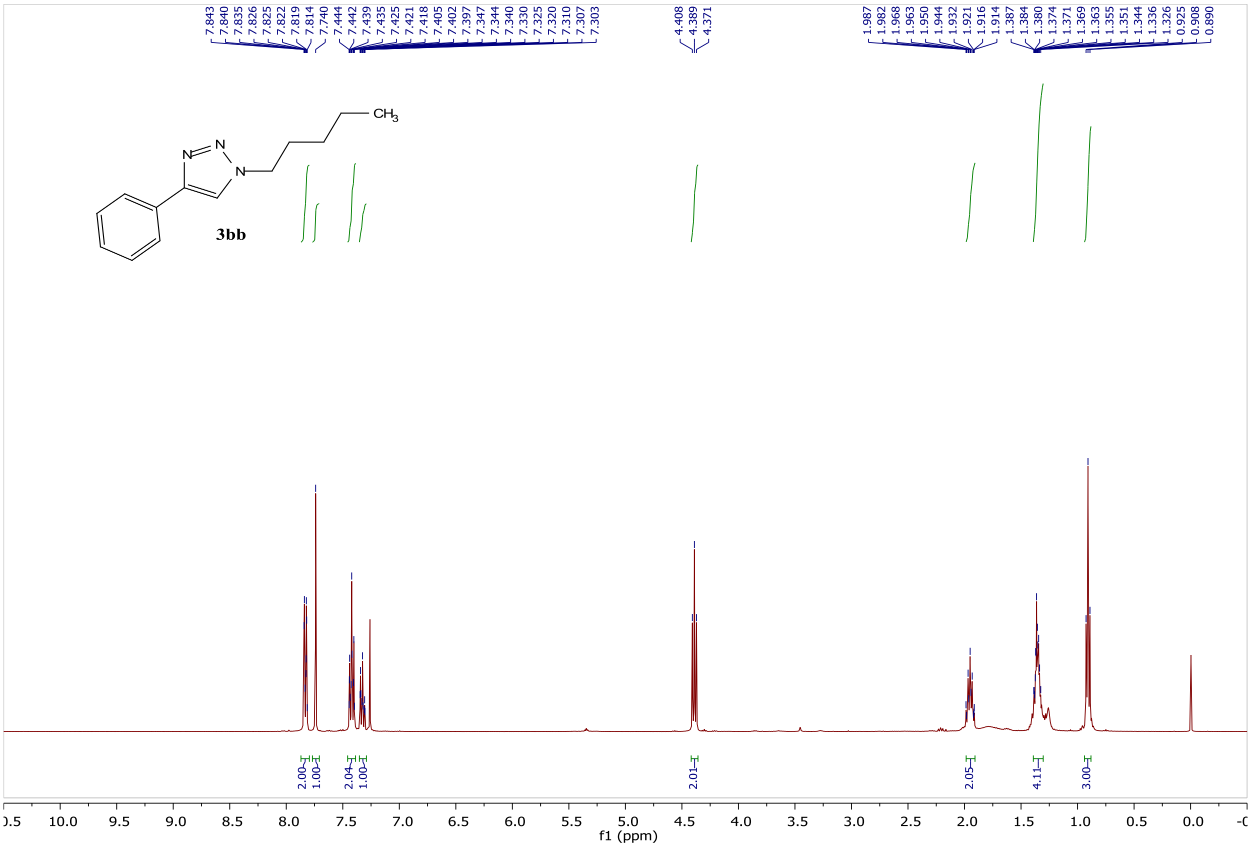
7.843 7.840 7.835 7.826 7.825 7.822 7.819 7.814 7.740 7.444 7.442 7.439 7.435 7.425 7.421 7.418 7.405 7.402 7.397 7.347 7.344 7.340 7.330 7.325 7.320 7.310 7.307 7.303
4.408 4.389 4.371
1.987 1.982 1.968 1.963 1.950 1.944 1.932 1.921 1.916 1.914 1.387 1.384 1.380 1.374 1.371 1.369 1.363 1.355 1.351 1.344 1.336 1.326 0.925 0.908 0.890
CH3
N
N
N
3bb
2.00
1.00
2.04
1.00
2.01
2.05
4.11
3.00
f1 (ppm)

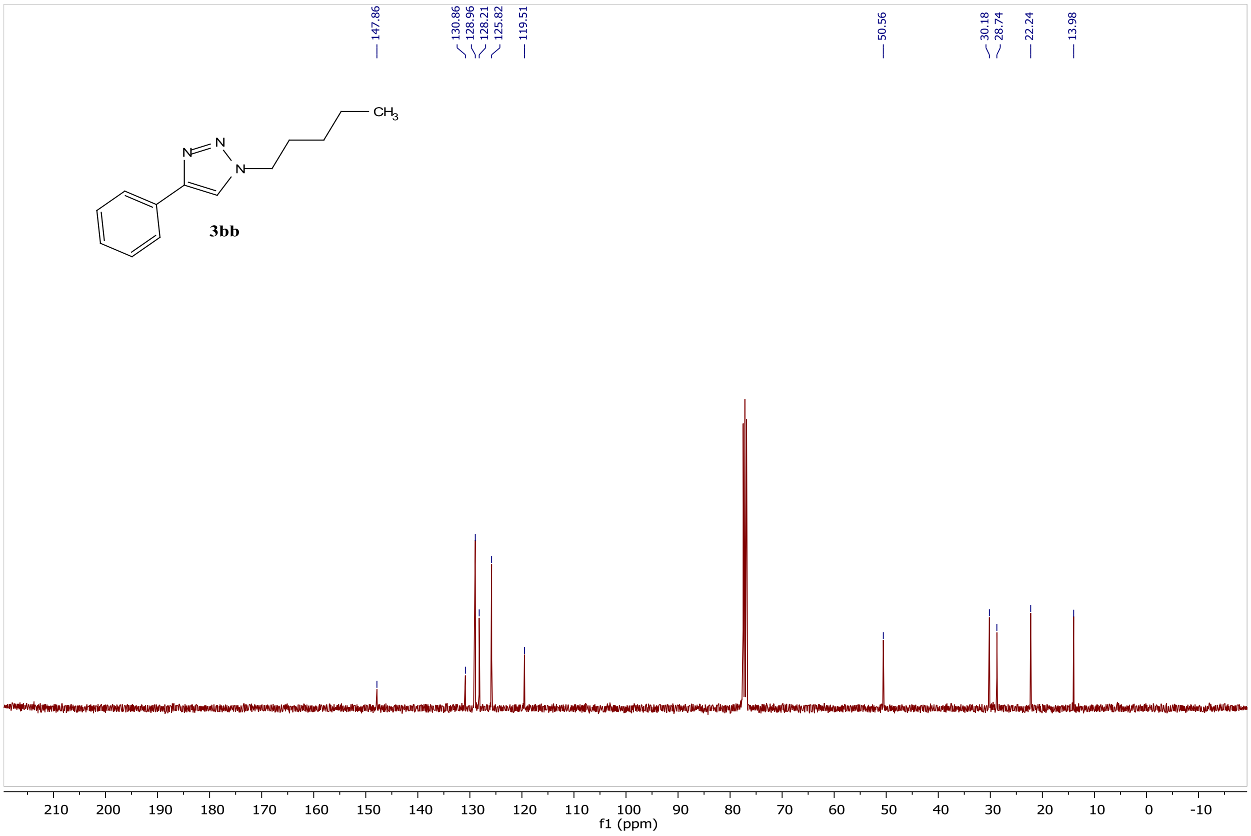
147.86
130.86 128.96 128.21 125.82
119.51
50.56
30.18 28.74
22.24
13.98
CH3
N
N
N
3bb
f1 (ppm)

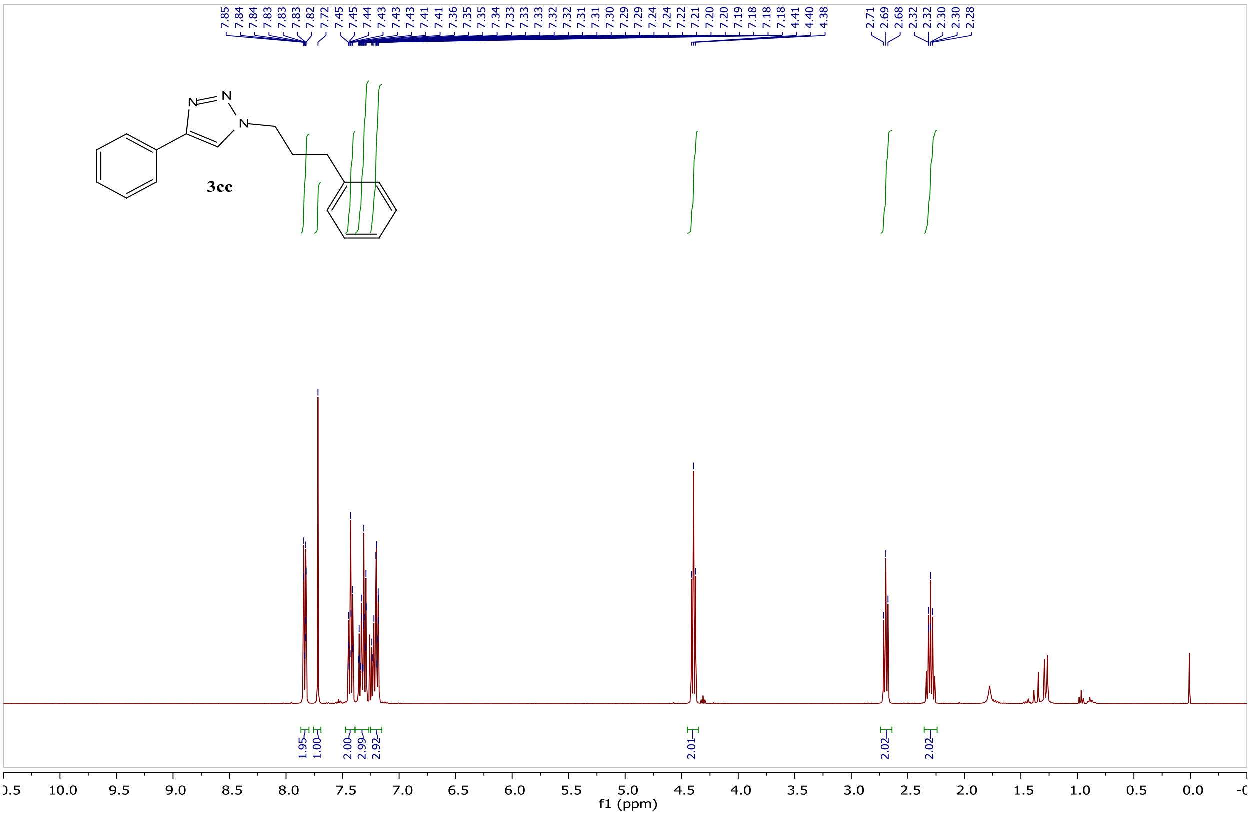
3cc
f1 (ppm)

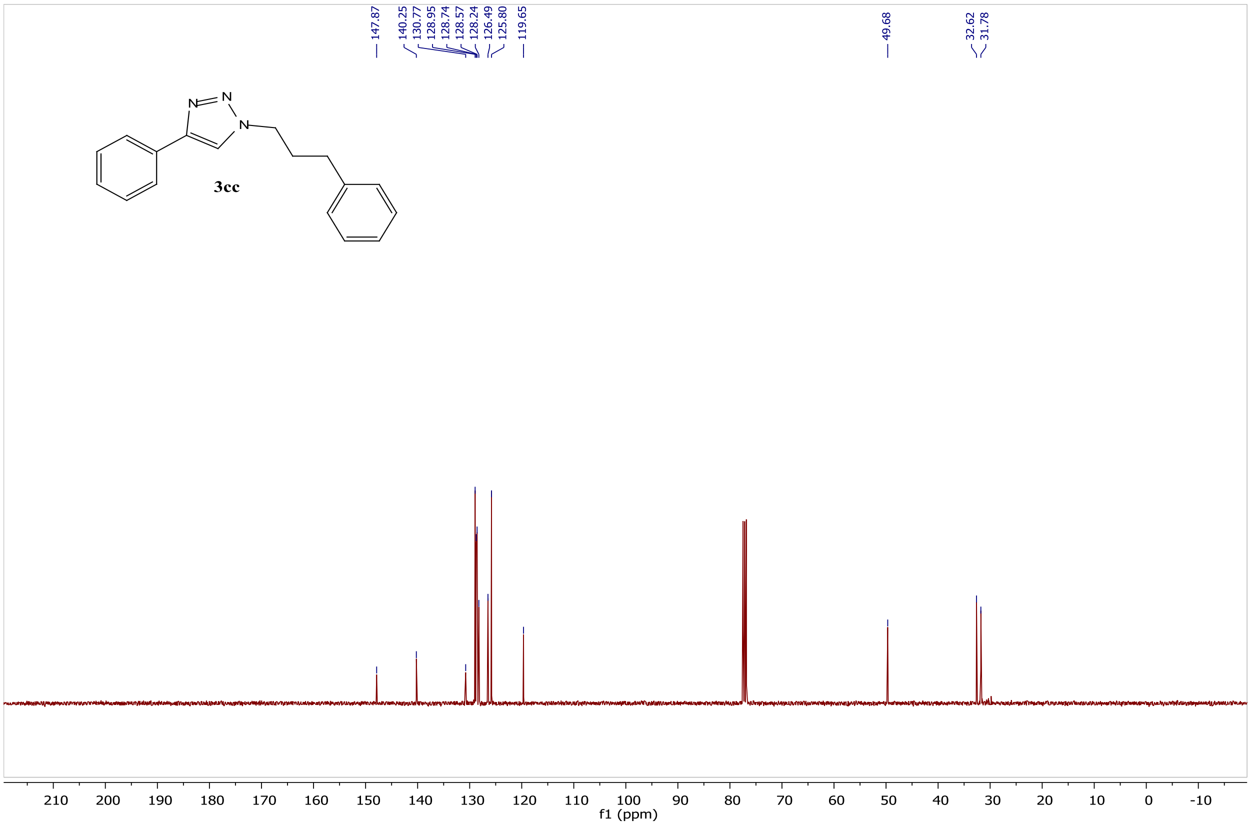
3cc
f1 (ppm)

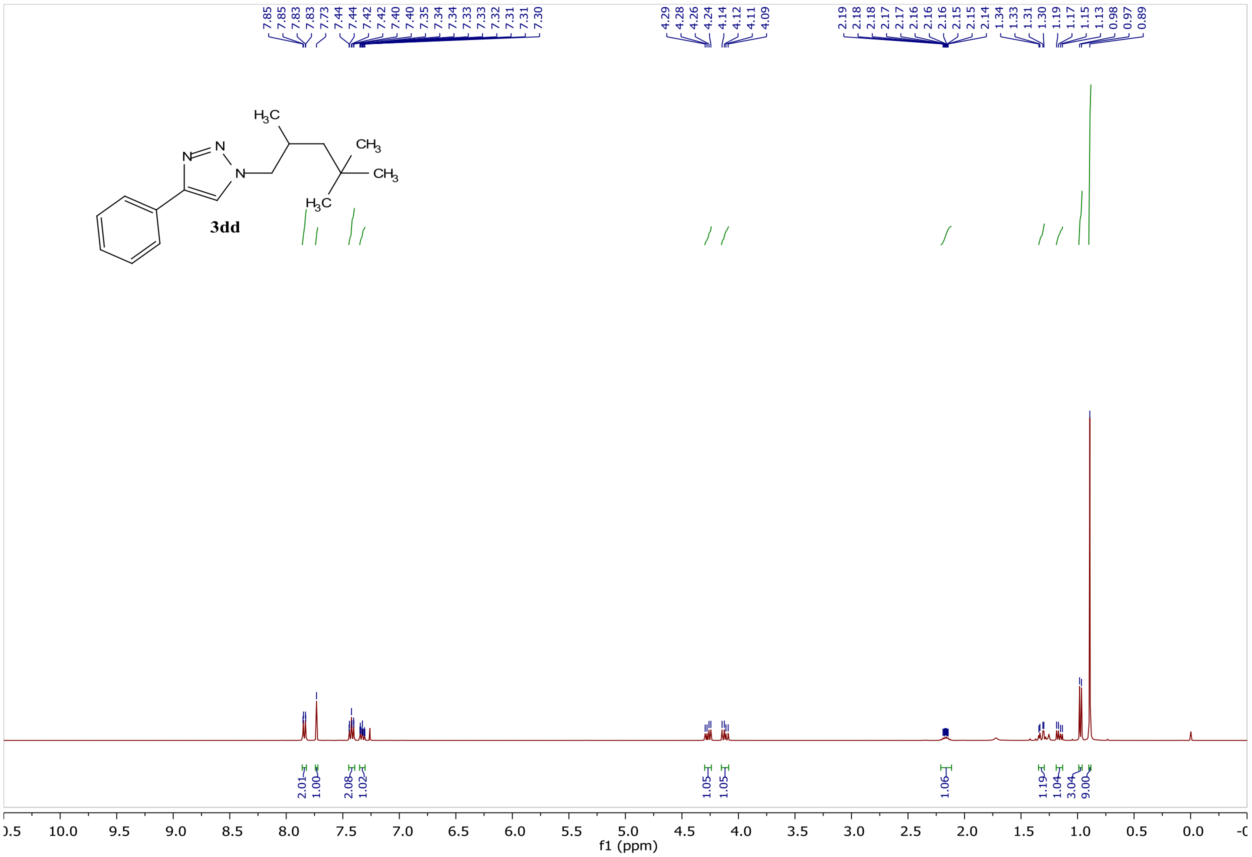
7.85 7.85 7.83 7.83 7.73 7.44 7.44 7.42 7.42 7.40 7.40 7.35 7.34 7.34 7.33 7.33 7.32 7.31 7.30
4.29 4.28 4.26 4.24 4.14 4.12 4.11 4.09
2.19 2.18 2.18 2.17 2.17 2.16 2.16 2.16 2.15 2.15 2.14 1.34 1.33 1.31 1.30 1.19 1.17 1.15 1.13 0.98 0.97 0.89
3dd
2.01 1.00 2.08 1.02 1.05 1.05 1.06 1.19 1.04 3.04 9.00
f1 (ppm)

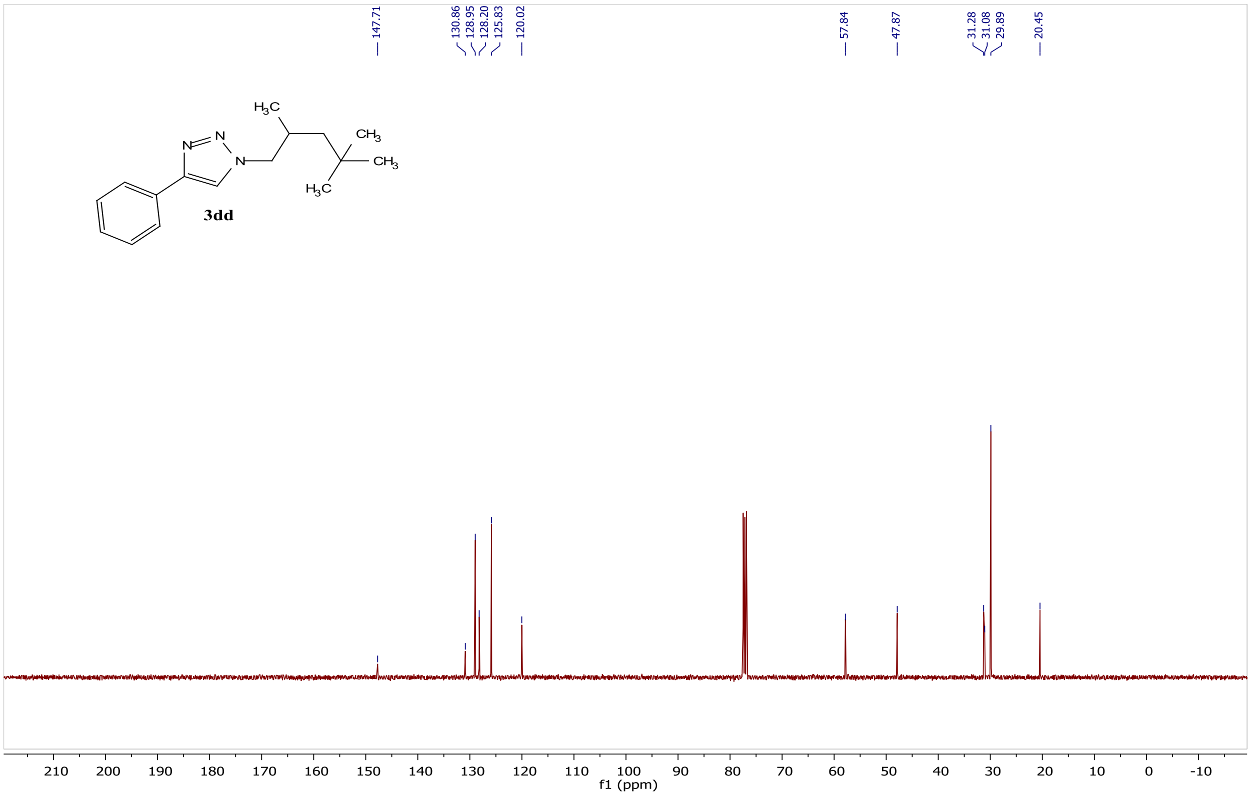
147.71
130.86 128.95 128.20 125.83 120.02
57.84
47.87
31.28 31.08 29.89
20.45
3dd
f1 (ppm)

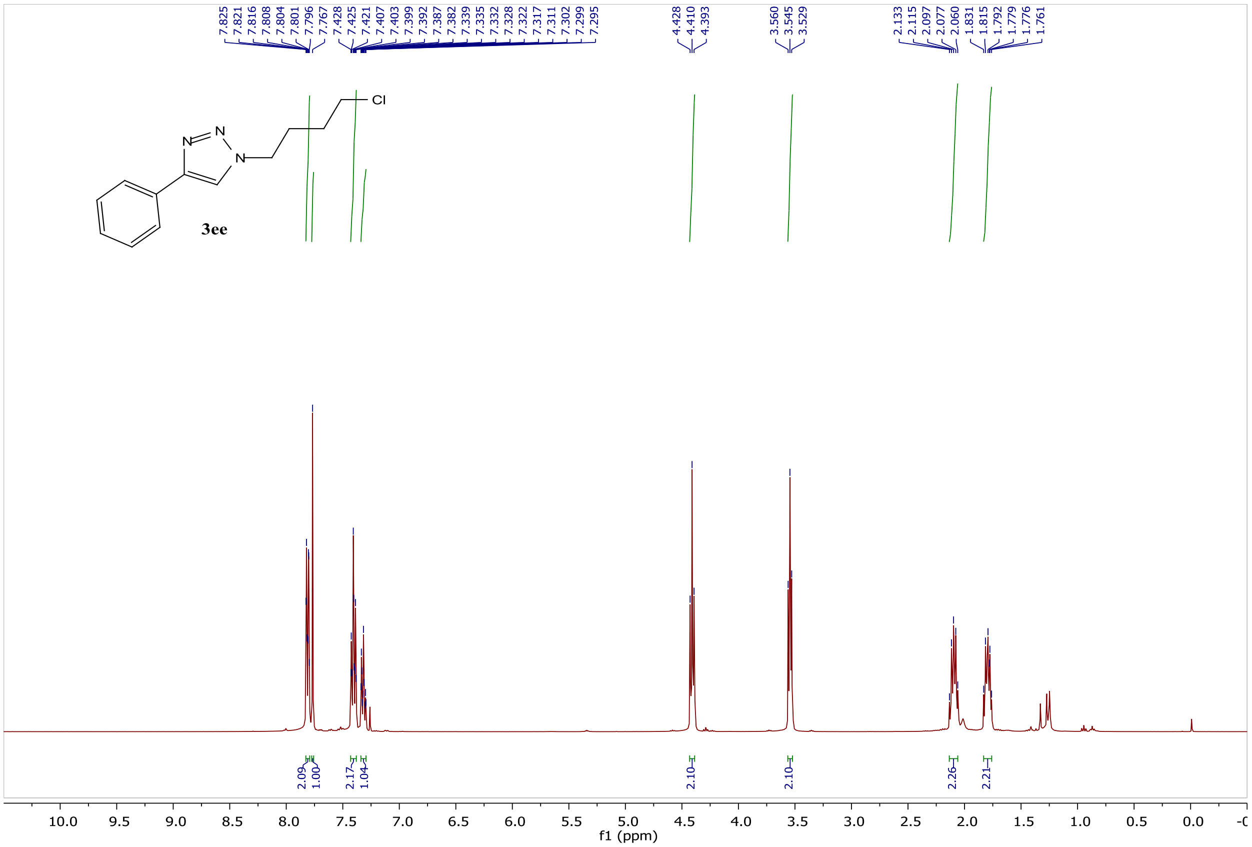
7.825 7.821 7.816 7.808 7.804 7.801 7.796 7.767 7.428 7.425 7.421 7.407 7.403 7.399 7.392 7.387 7.382 7.339 7.335 7.332 7.328 7.322 7.317 7.311 7.302 7.299 7.295
4.428 4.410 4.393
3.560 3.545 3.529
2.133 2.115 2.097 2.077 2.060 1.831 1.815 1.792 1.779 1.776 1.761
N
N
N
Cl
3ee
2.09 1.00 2.17 1.04 2.10 2.10 2.26 2.21
f1 (ppm)

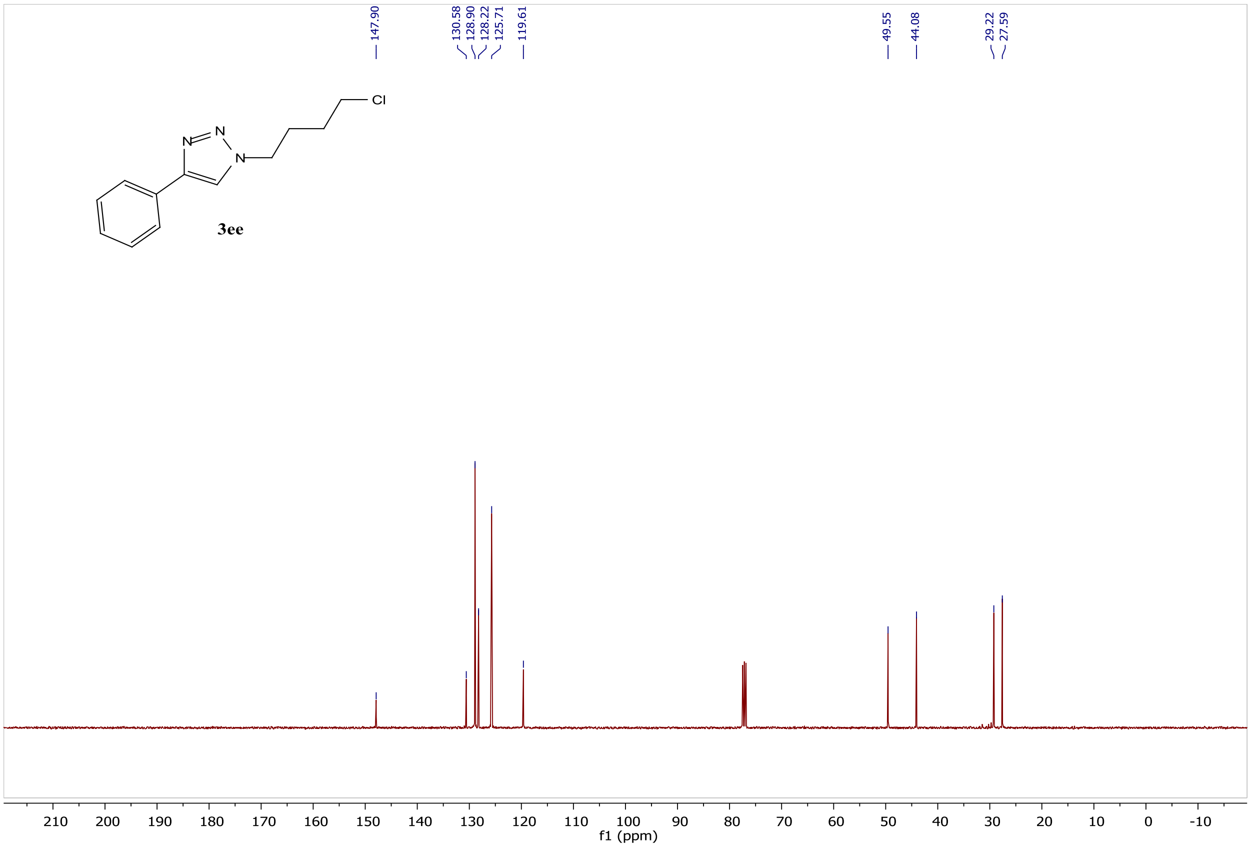
147.90
130.58 128.90 128.22 125.71 119.61
49.55 44.08
29.22 27.59
N
N
N
Cl
3ee
f1 (ppm)

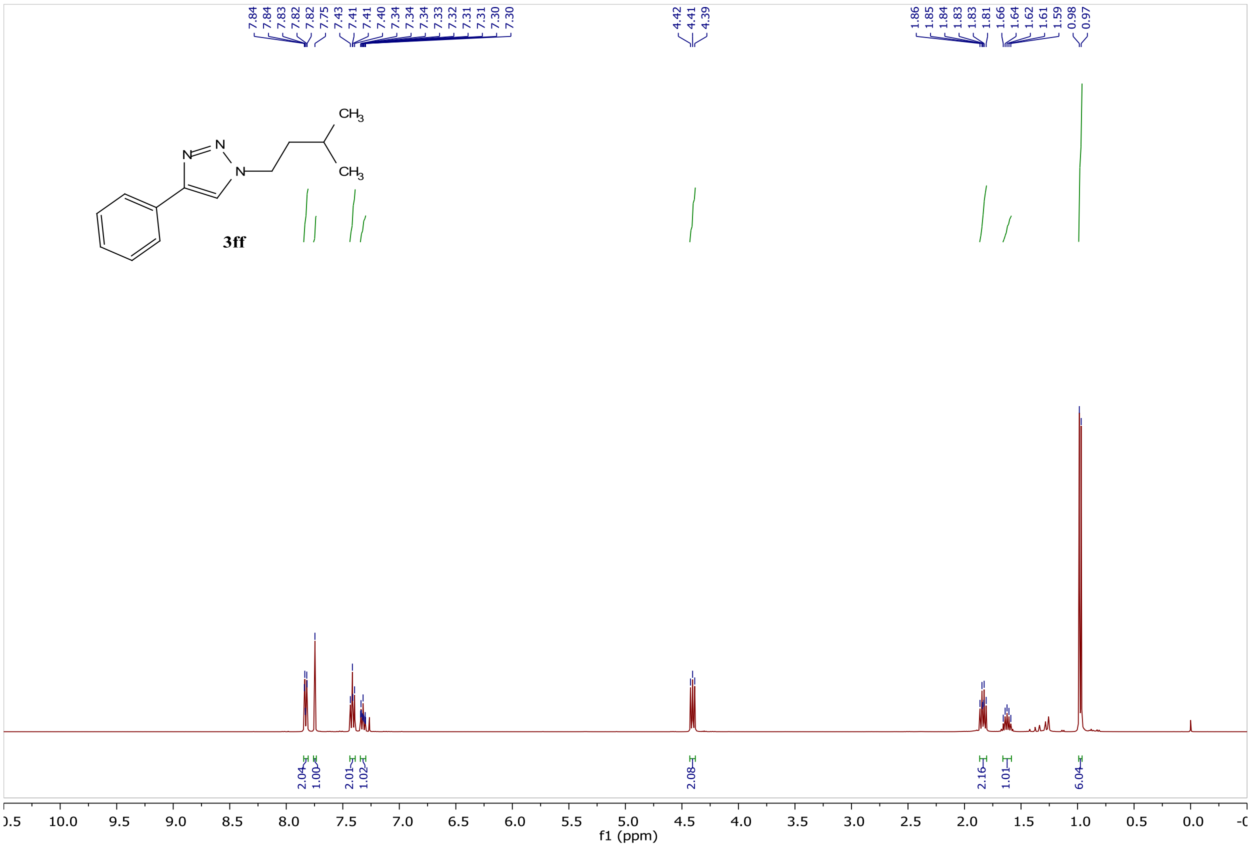
7.84
7.84
7.83
7.82
7.82
7.75
7.43
7.41
7.41
7.40
7.34
7.34
7.34
7.33
7.32
7.31
7.31
7.30
7.30
4.42
4.41
4.39
1.86
1.85
1.84
1.83
1.83
1.81
1.66
1.64
1.62
1.61
1.59
0.98
0.97
CH3
CH3
N
N
N
3ff
2.04
1.00
2.01
1.02
2.08
2.16
1.01
6.04
f1 (ppm)

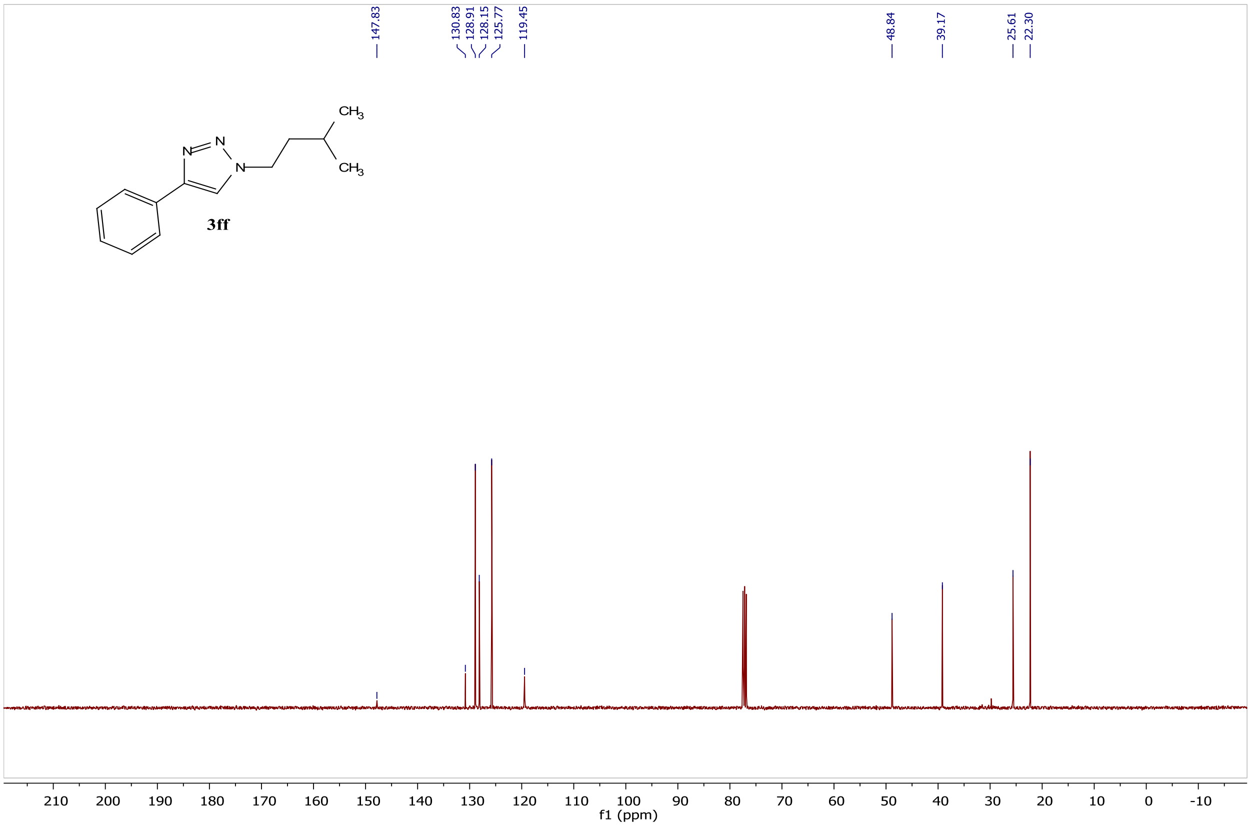
147.83
130.83
128.91
128.15
125.77
119.45
48.84
39.17
25.61
22.30
CH3
CH3
N
N
N
3ff
f1 (ppm)

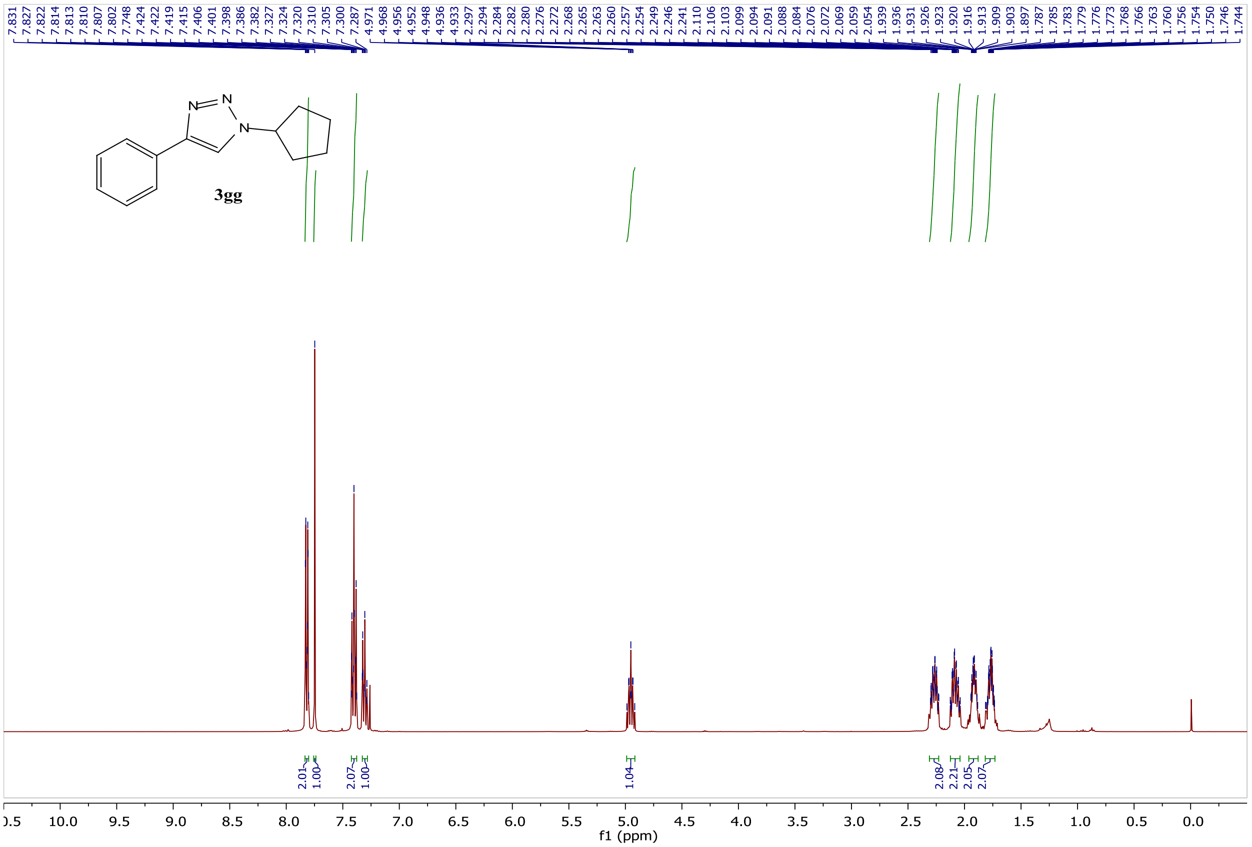
3gg
7.831 7.827 7.822 7.814 7.813 7.810 7.807 7.802 7.748 7.424 7.422 7.419 7.415 7.406 7.401 7.398 7.386 7.382 7.327 7.324 7.320 7.310 7.305 7.300 7.287 4.971 4.968 4.956 4.952 4.948 4.936 4.933 2.297 2.294 2.284 2.282 2.280 2.276 2.272 2.268 2.265 2.263 2.260 2.257 2.254 2.249 2.246 2.241 2.110 2.106 2.103 2.099 2.094 2.091 2.088 2.084 2.076 2.072 2.069 2.059 2.054 1.939 1.936 1.931 1.926 1.923 1.920 1.916 1.913 1.909 1.903 1.897 1.787 1.785 1.783 1.779 1.776 1.773 1.768 1.766 1.763 1.760 1.756 1.754 1.750 1.746 1.744
2.01 1.00 2.07 1.00 1.04 2.08 2.21 2.05 2.07
f1 (ppm)

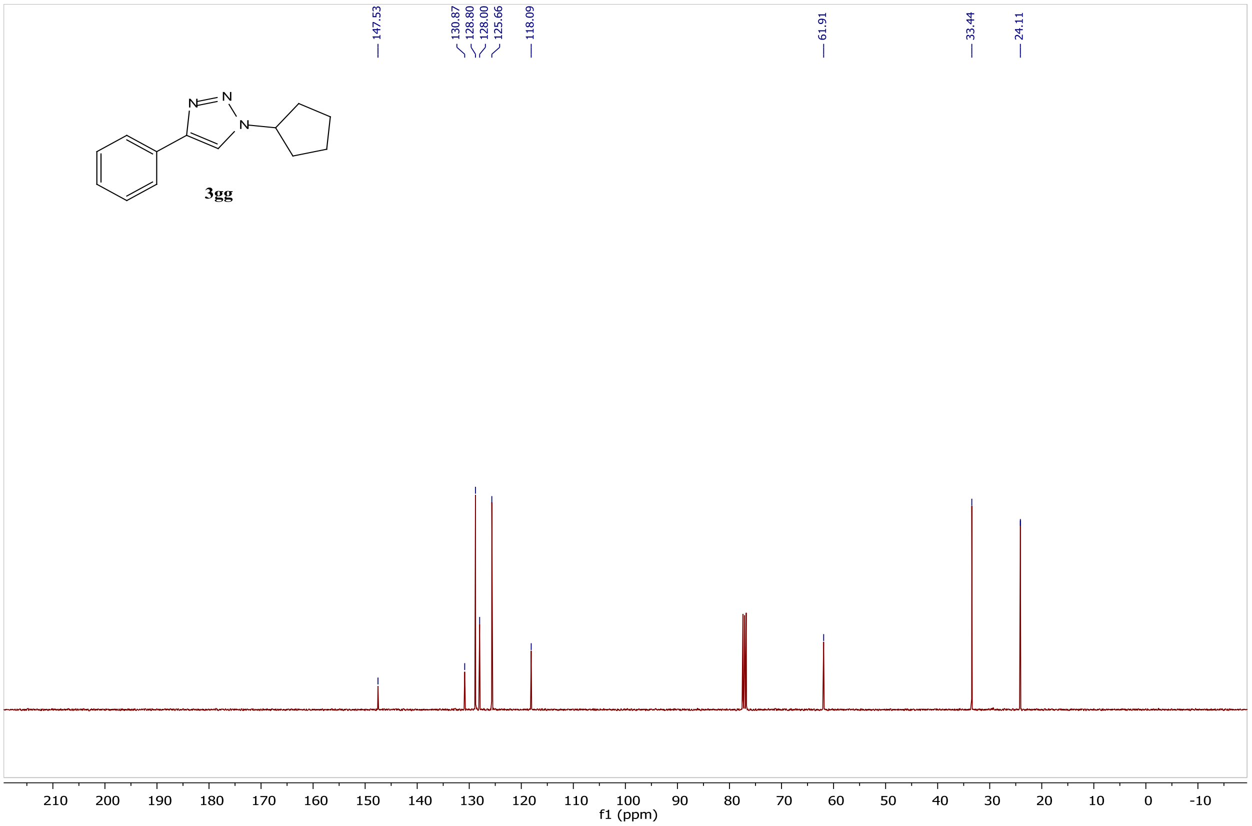
3gg
147.53 130.87 128.80 128.00 125.66 118.09 61.91 33.44 24.11
f1 (ppm)

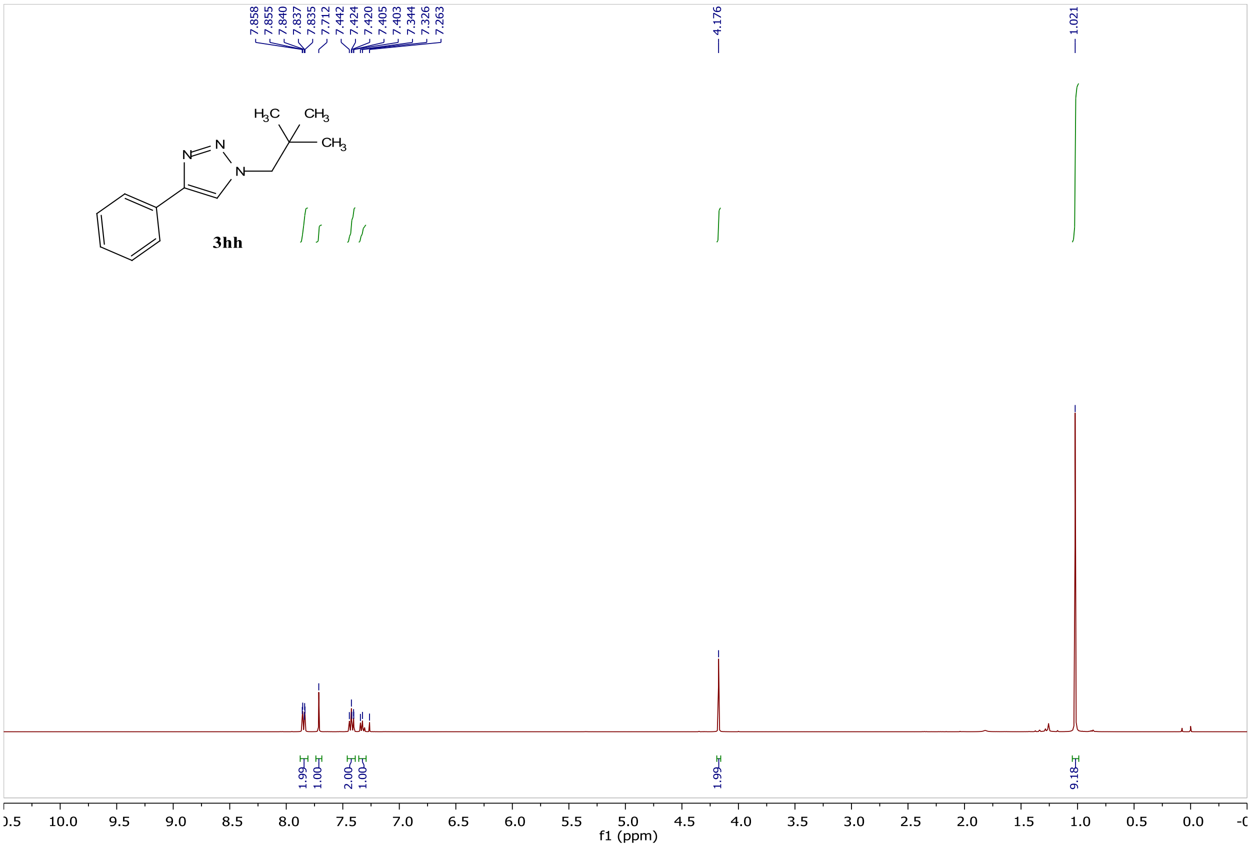
7.858
7.855
7.840
7.837
7.835
7.712
7.442
7.424
7.420
7.405
7.403
7.344
7.326
7.263
4.176
1.021
3hh
1.99
1.00
2.00
1.00
1.99
9.18
f1 (ppm)

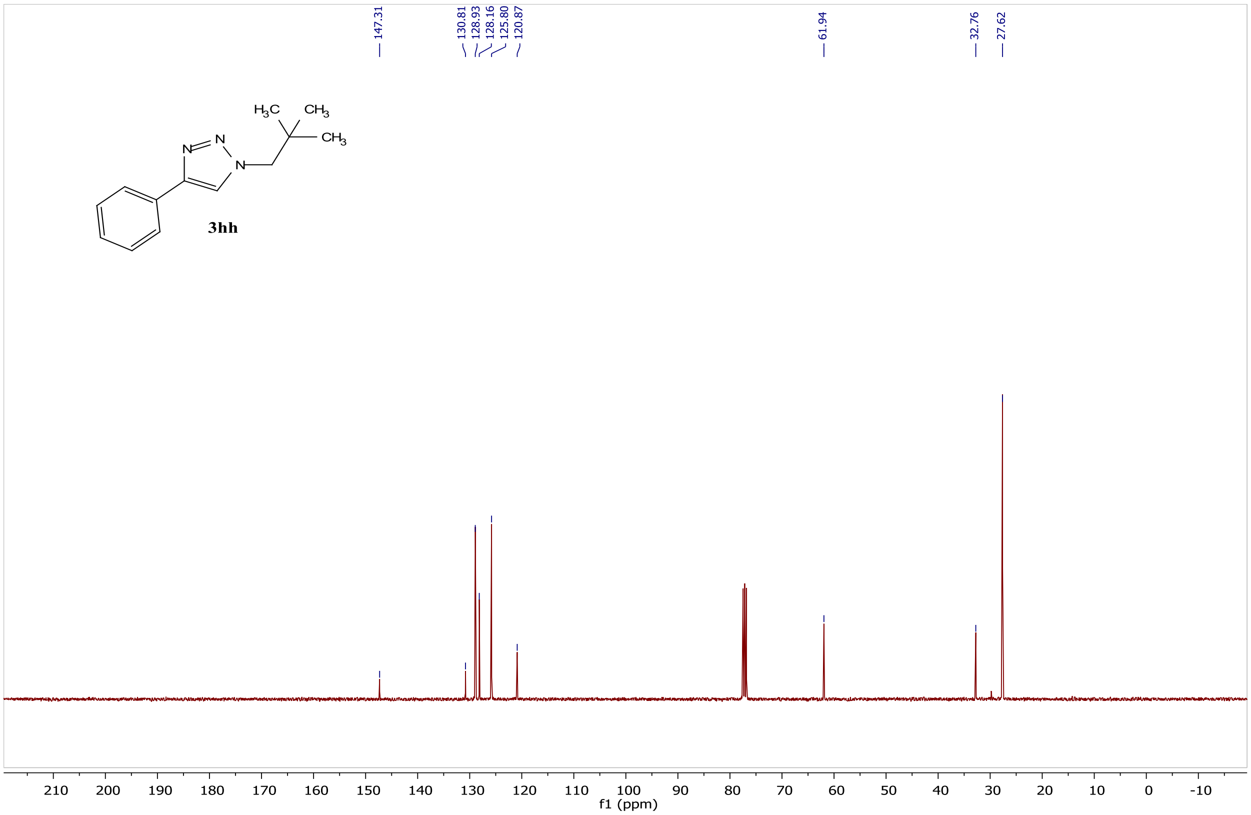
147.31
130.81
128.93
128.16
125.80
120.87
61.94
32.76
27.62
3hh
f1 (ppm)

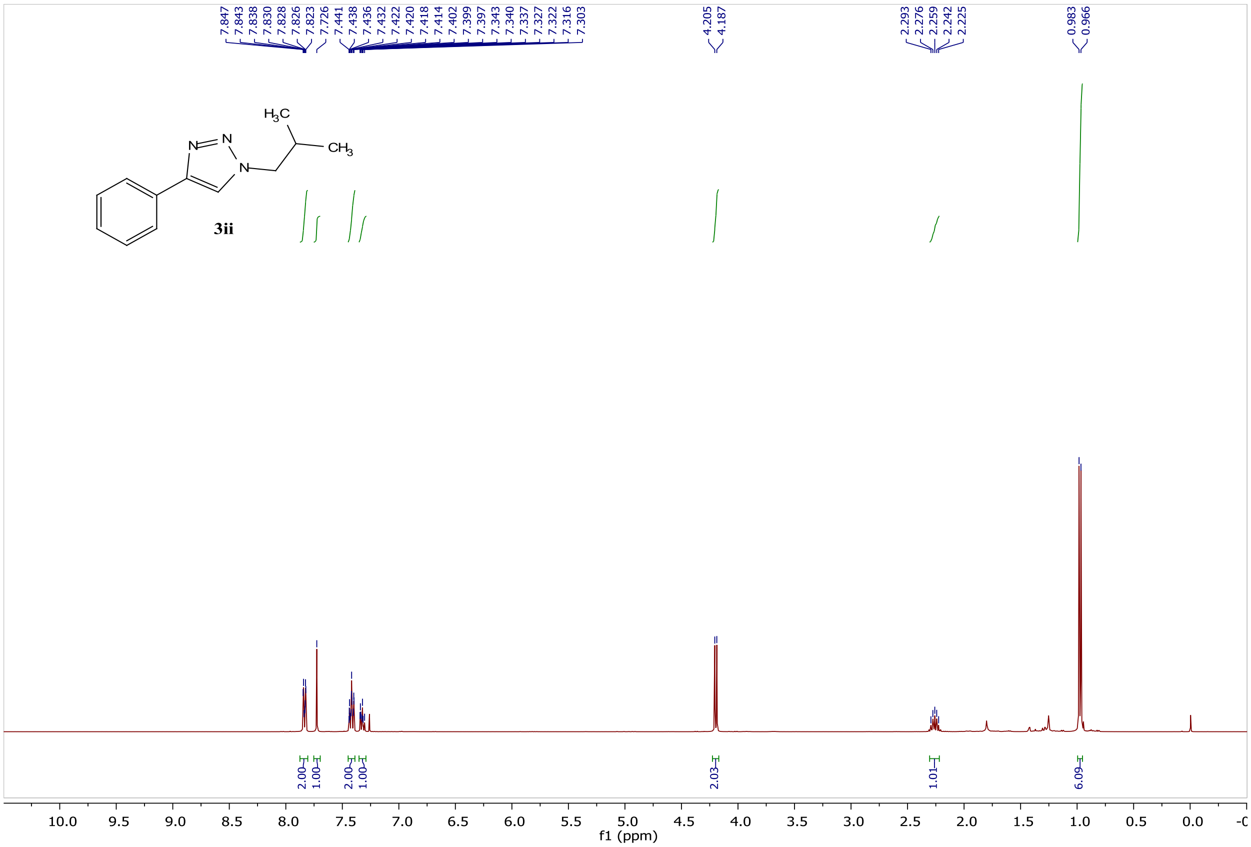
7.847
7.843
7.838
7.830
7.828
7.826
7.823
7.726
7.441
7.438
7.436
7.432
7.422
7.420
7.418
7.414
7.402
7.399
7.397
7.343
7.340
7.337
7.327
7.322
7.316
7.303
4.205
4.187
2.293
2.276
2.259
2.242
2.225
0.983
0.966
H_3C
CH_3
N
N
N
3ii
2.00
1.00
2.00
1.00
2.03
1.01
6.09
f1 (ppm)

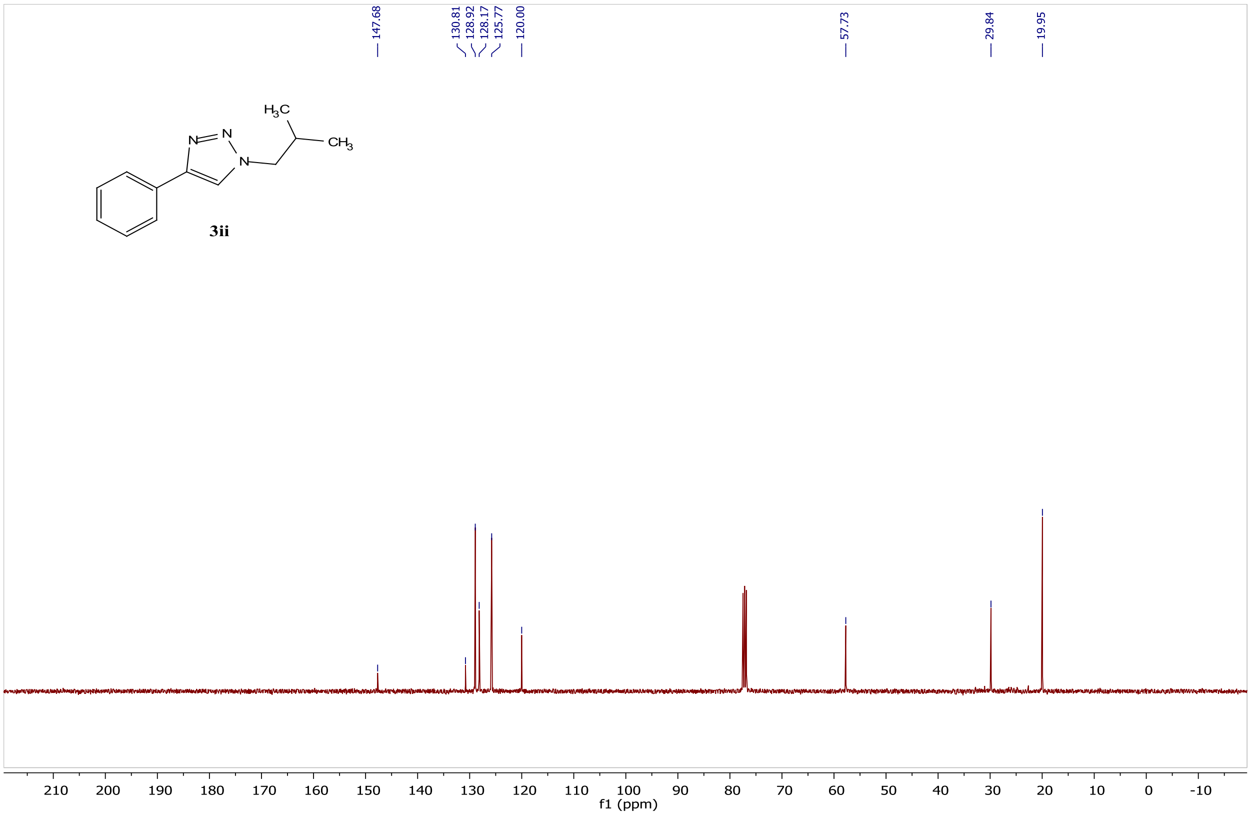
147.68
130.81
128.92
128.17
125.77
120.00
57.73
29.84
19.95
H_3C
CH_3
N
N
N
3ii
f1 (ppm)

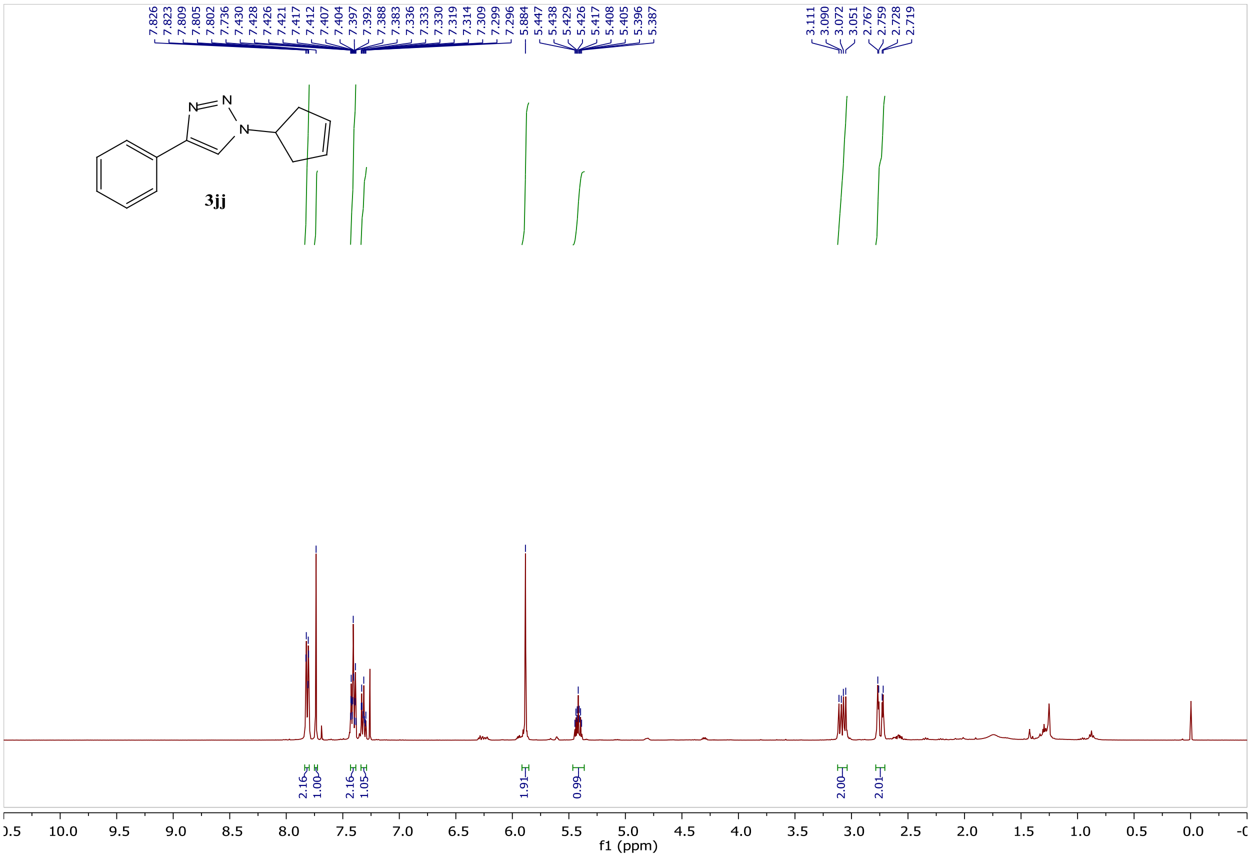
7.826 7.823 7.809 7.805 7.802 7.736 7.430 7.428 7.426 7.421 7.417 7.412 7.407 7.404 7.397 7.392 7.388 7.383 7.336 7.333 7.330 7.319 7.314 7.309 7.299 7.296
5.884
5.447 5.438 5.429 5.426 5.417 5.408 5.405 5.396 5.387
3.111 3.090 3.072 3.051 2.767 2.759 2.728 2.719
N
N
N
3jj
2.16
1.00
2.16
1.05
1.91
0.99
2.00
2.01
10.0 9.5 9.0 8.5 8.0 7.5 7.0 6.5 6.0 5.5 5.0 4.5 4.0 3.5 3.0 2.5 2.0 1.5 1.0 0.5 0.0
f1 (ppm)

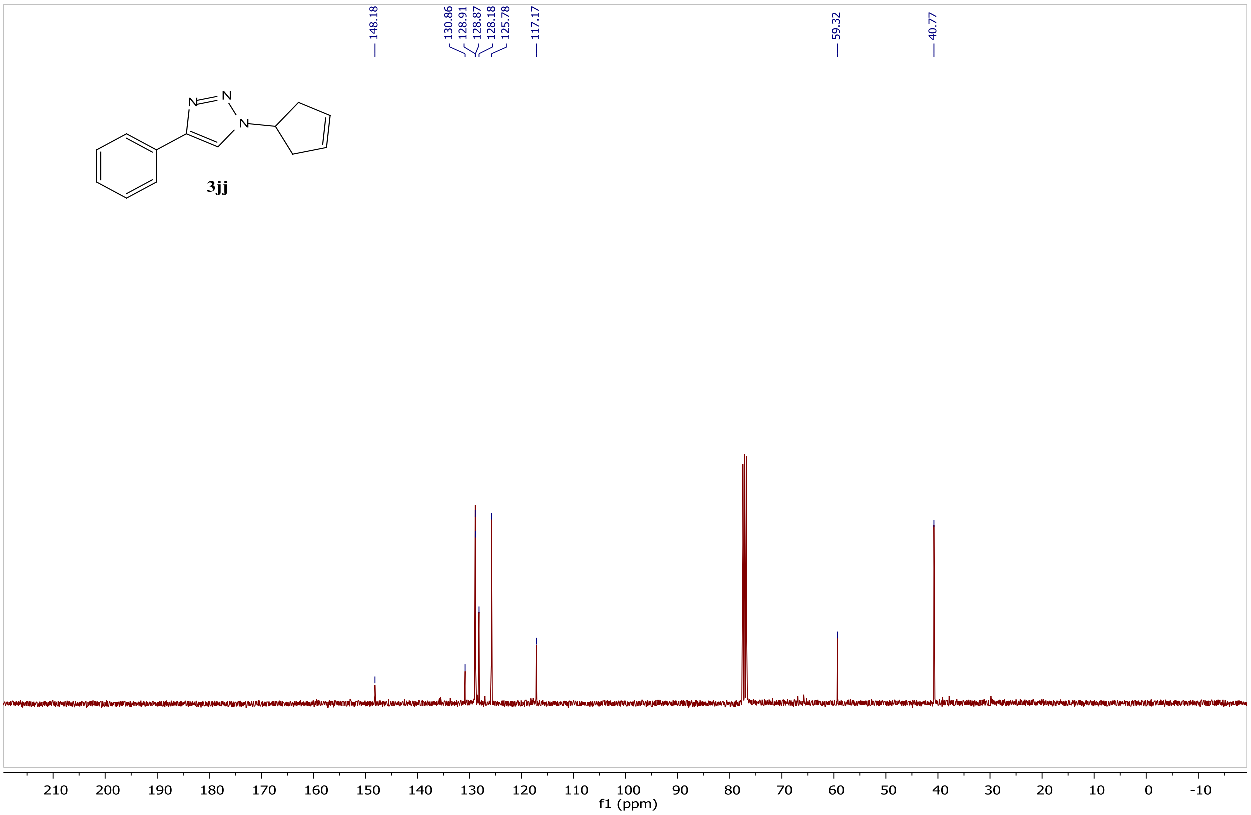
148.18
130.86 128.91 128.87 128.18 125.78
117.17
59.32
40.77
N
N
N
3jj
210 200 190 180 170 160 150 140 130 120 110 100 90 80 70 60 50 40 30 20 10 0 -10
f1 (ppm)

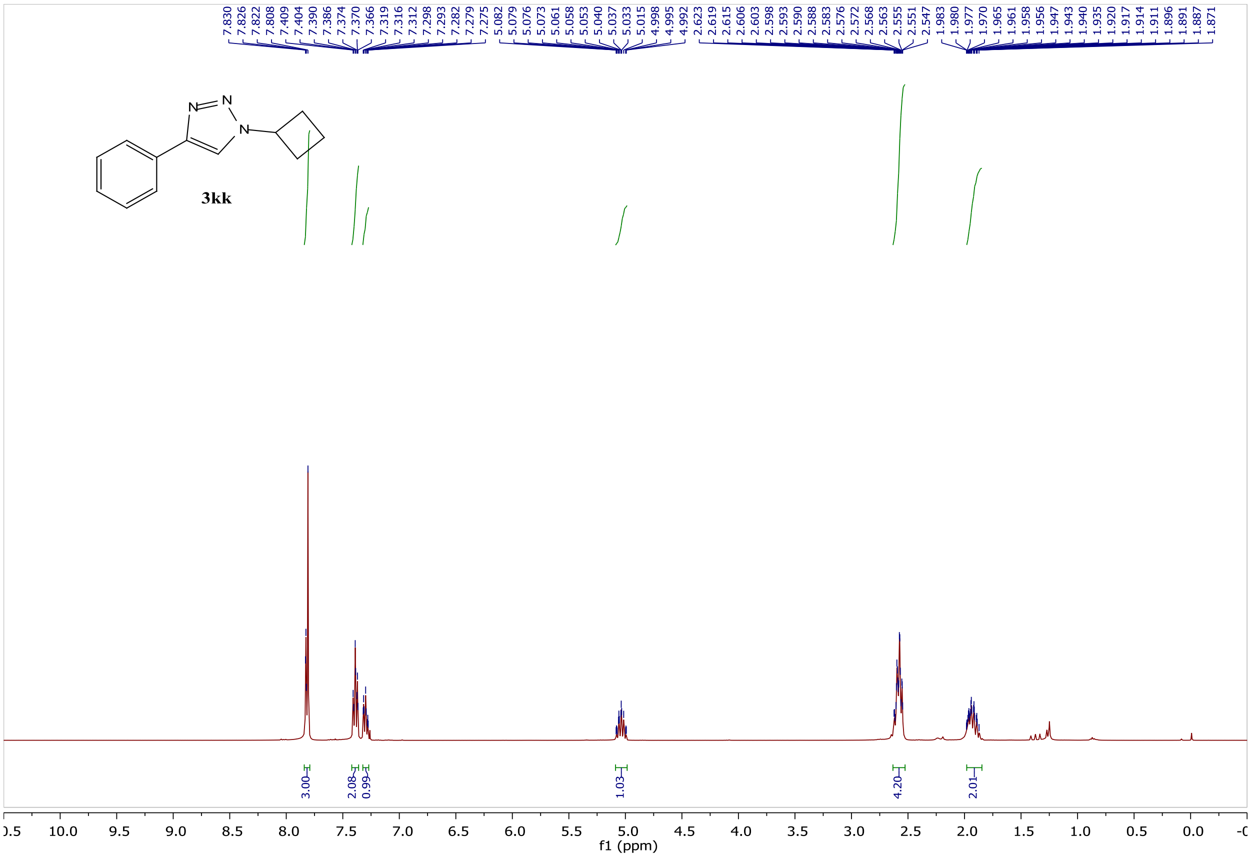
7.830 7.826 7.822 7.808 7.409 7.404 7.390 7.386 7.374 7.370 7.366 7.319 7.316 7.312 7.298 7.293 7.282 7.279 7.275 5.082 5.079 5.076 5.073 5.061 5.058 5.053 5.040 5.037 5.033 5.015 4.998 4.995 4.992 2.623 2.619 2.615 2.606 2.603 2.598 2.593 2.590 2.588 2.583 2.576 2.572 2.568 2.563 2.555 2.551 2.547 1.983 1.980 1.977 1.970 1.965 1.961 1.958 1.956 1.947 1.943 1.940 1.935 1.920 1.917 1.914 1.911 1.896 1.891 1.887 1.871
N
N
N
3kk
3.00
2.08
0.99
1.03
4.20
2.01
f1 (ppm)

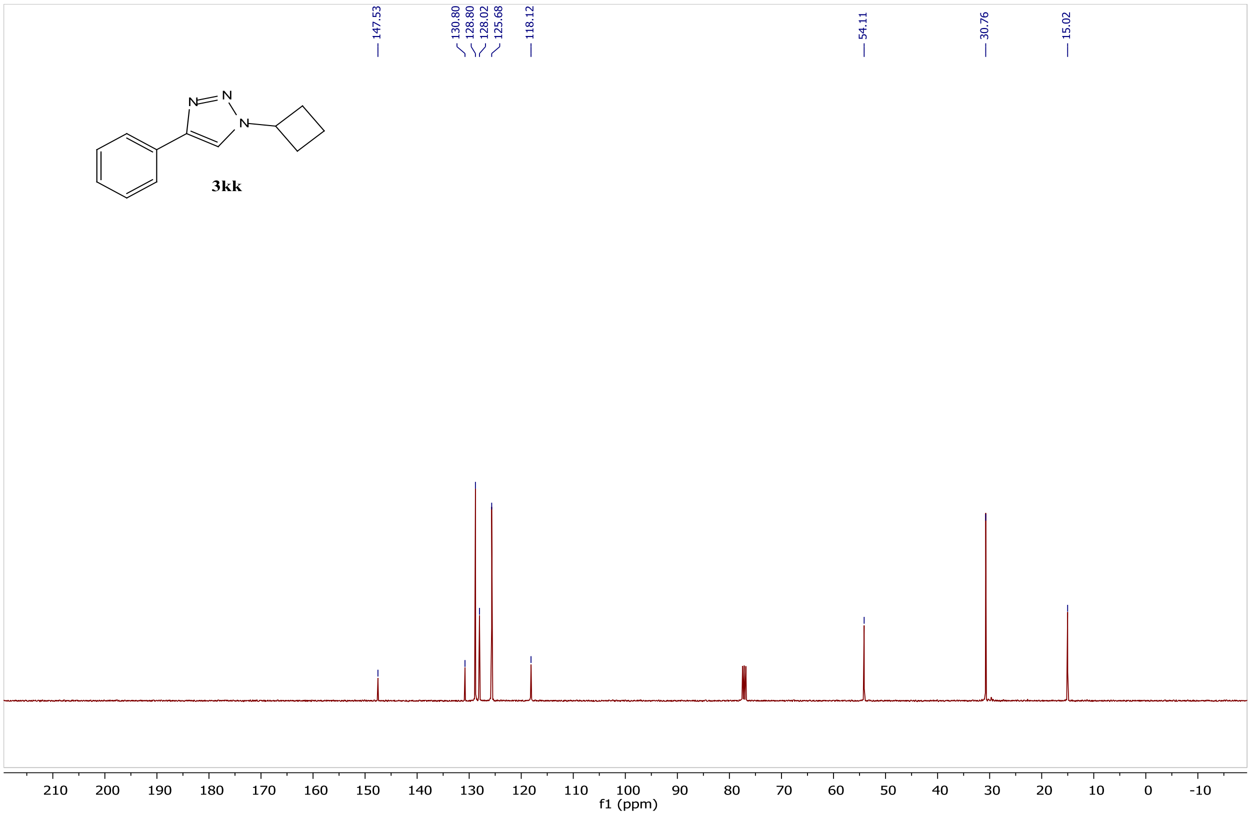
147.53
130.80
128.80
128.02
125.68
118.12
54.11
30.76
15.02
N
N
N
3kk
f1 (ppm)

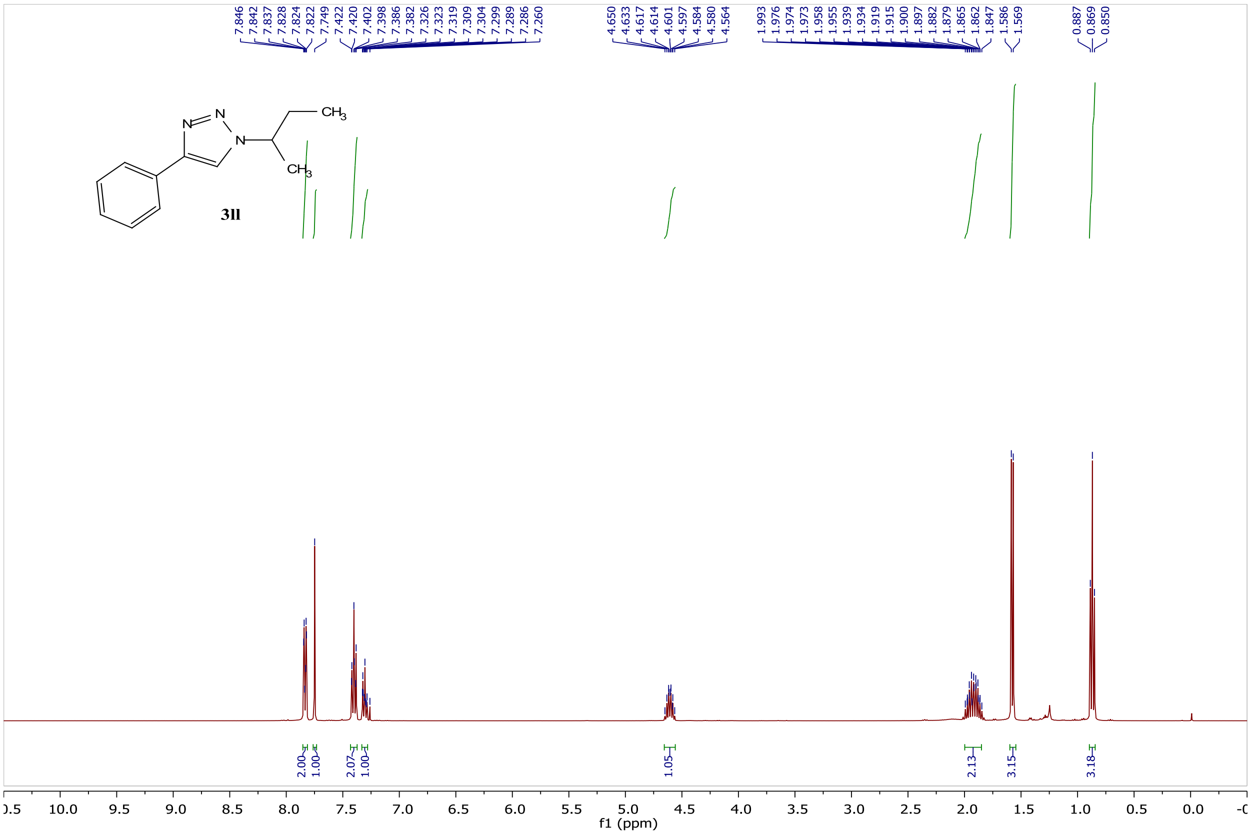
7.846
7.842
7.837
7.828
7.824
7.822
7.749
7.422
7.420
7.402
7.398
7.386
7.382
7.326
7.323
7.319
7.309
7.304
7.299
7.289
7.286
7.260
4.650
4.633
4.617
4.614
4.601
4.597
4.584
4.580
4.564
1.993
1.976
1.974
1.973
1.958
1.955
1.939
1.934
1.919
1.915
1.900
1.897
1.882
1.879
1.865
1.862
1.847
1.586
1.569
0.887
0.869
0.850
N
N
N
CH3
CH3
3ll
2.00
1.00
2.07
1.00
1.05
2.13
3.15
3.18
f1 (ppm)

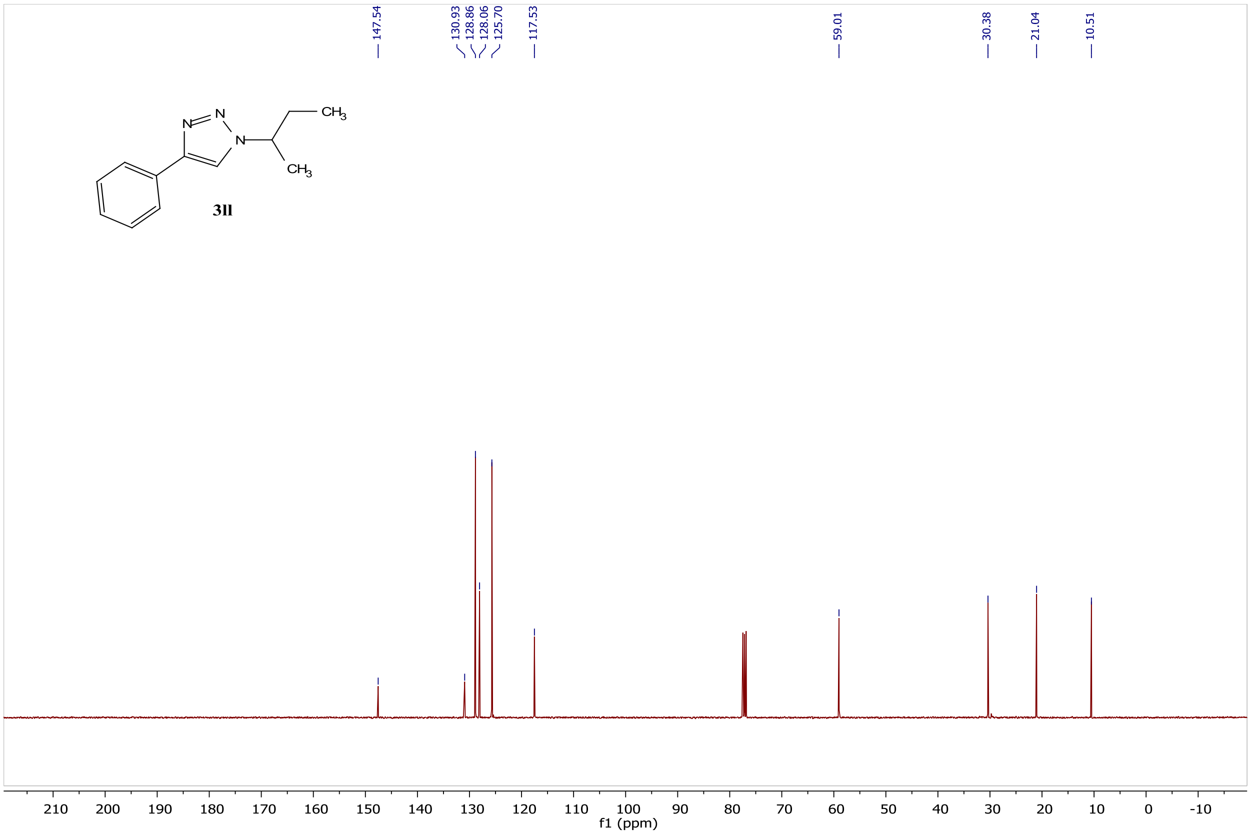
147.54
130.93
128.86
128.06
125.70
117.53
59.01
30.38
21.04
10.51
N
N
N
CH3
CH3
3ll
f1 (ppm)

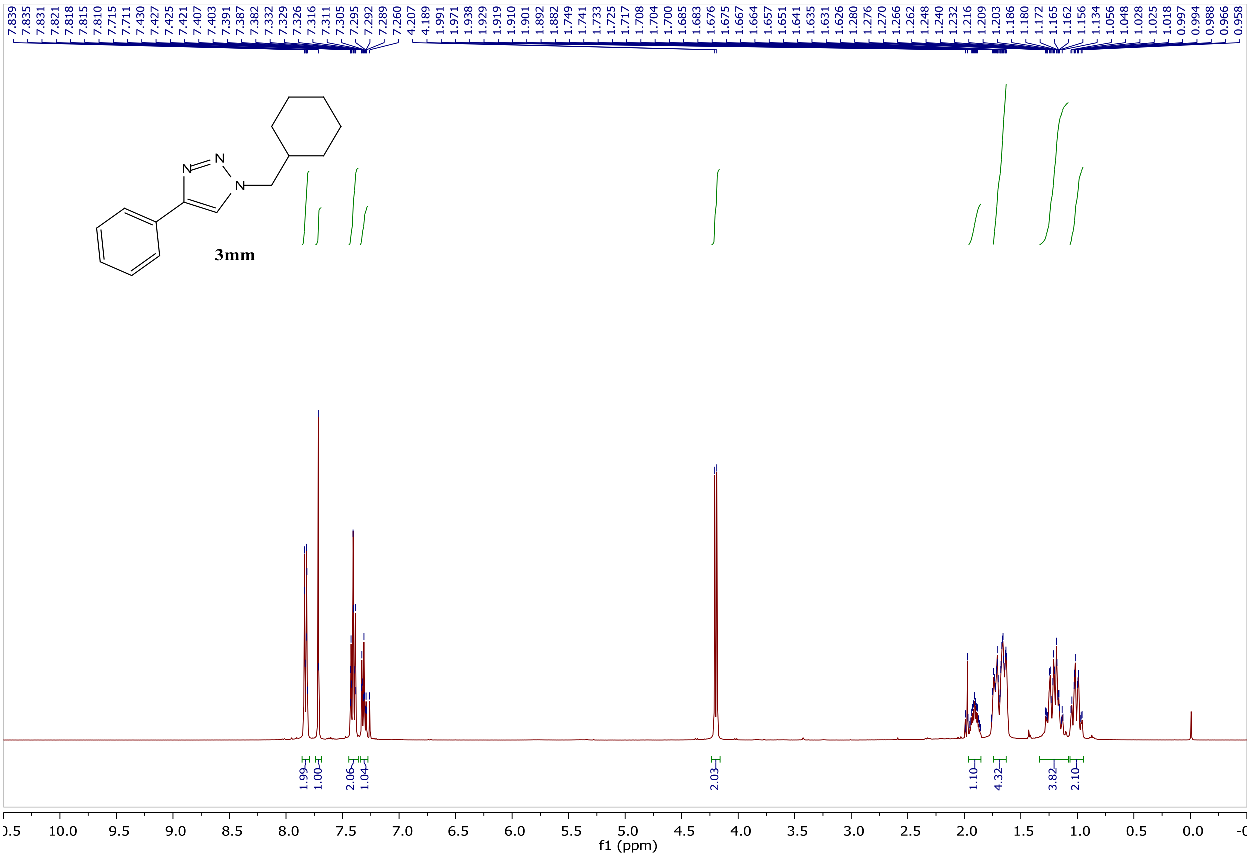
7.839 7.835 7.831 7.821 7.818 7.815 7.810 7.715 7.711 7.430 7.427 7.425 7.421 7.407 7.403 7.391 7.387 7.382 7.332 7.329 7.326 7.316 7.311 7.305 7.295 7.292 7.289 7.260
4.207 4.189 1.991 1.971 1.938 1.929 1.919 1.910 1.901 1.892 1.882 1.749 1.741 1.733 1.725 1.717 1.708 1.704 1.700 1.685 1.683 1.676 1.675 1.667 1.664 1.657 1.651 1.641 1.635 1.631 1.626 1.280 1.276 1.270 1.266 1.262 1.248 1.240 1.232 1.216 1.209 1.203 1.186 1.180 1.172 1.165 1.162 1.156 1.134 1.056 1.048 1.028 1.025 1.018 0.997 0.994 0.988 0.966 0.958
3mm
1.99 1.00 2.06 1.04 2.03 1.10 4.32 3.82 2.10
f1 (ppm)

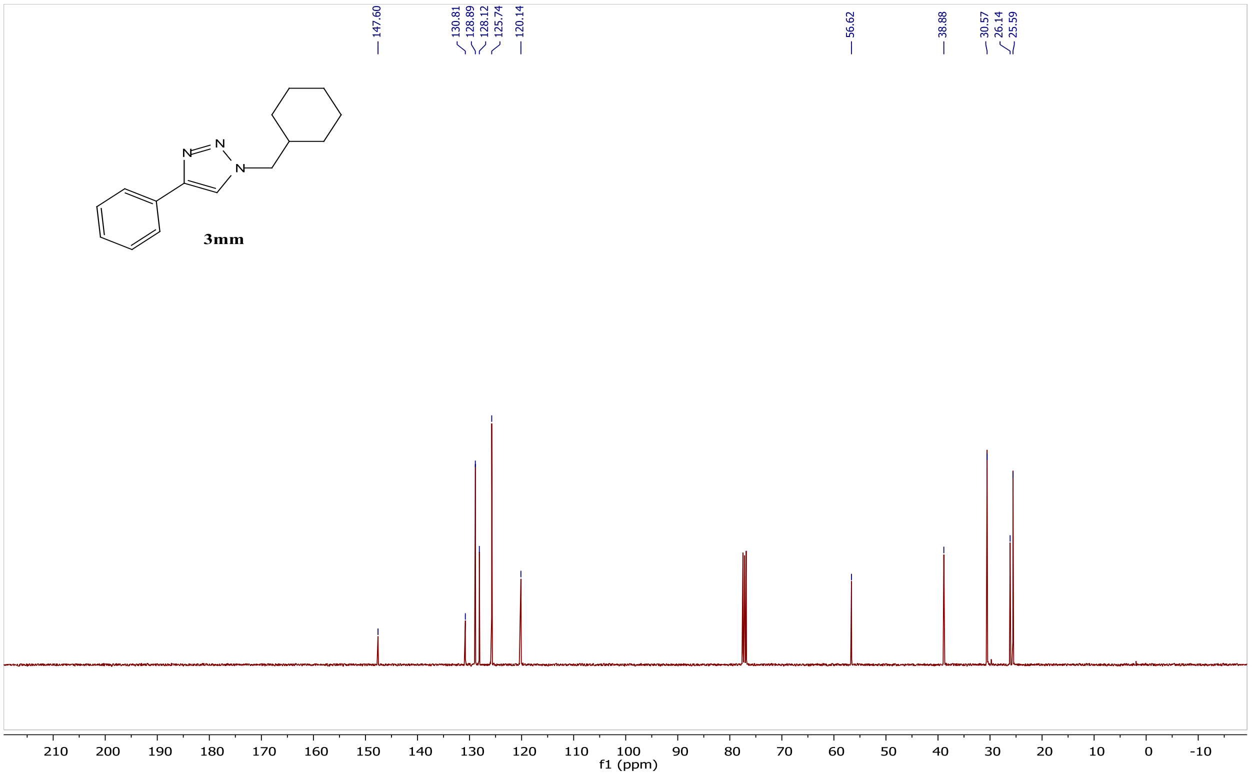
147.60
130.81 128.89 128.12 125.74 120.14
56.62
38.88
30.57 26.14 25.59
3mm
f1 (ppm)

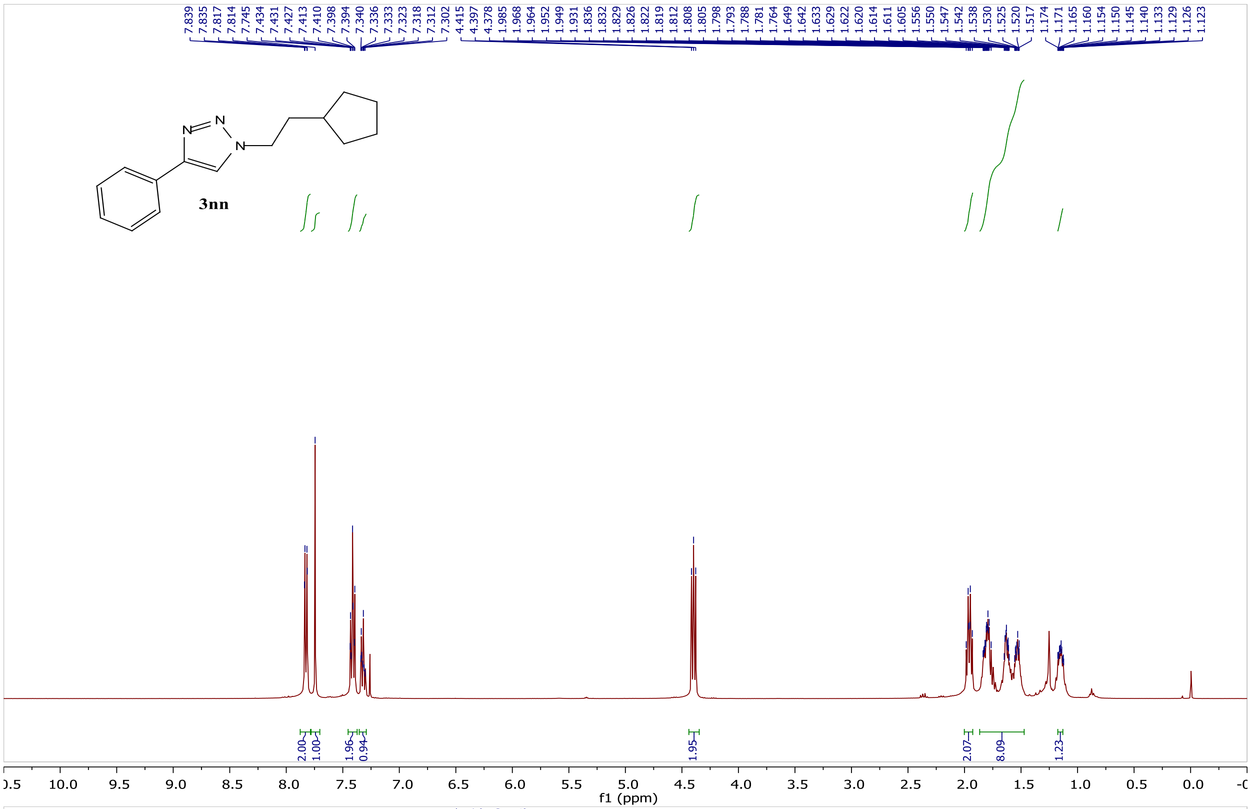
7.839 7.835 7.817 7.814 7.745 7.434 7.431 7.427 7.413 7.410 7.398 7.394 7.340 7.336 7.333 7.323 7.318 7.312 7.302 4.415 4.397 4.378 1.985 1.968 1.964 1.952 1.949 1.931 1.836 1.832 1.829 1.826 1.822 1.819 1.812 1.808 1.805 1.798 1.793 1.788 1.781 1.764 1.649 1.642 1.633 1.629 1.622 1.620 1.614 1.611 1.605 1.556 1.550 1.547 1.542 1.538 1.530 1.525 1.520 1.517 1.174 1.171 1.165 1.160 1.154 1.150 1.145 1.140 1.133 1.129 1.126 1.123
3nn
2.00 1.00 1.96 0.94 1.95 2.07 8.09 1.23
f1 (ppm)

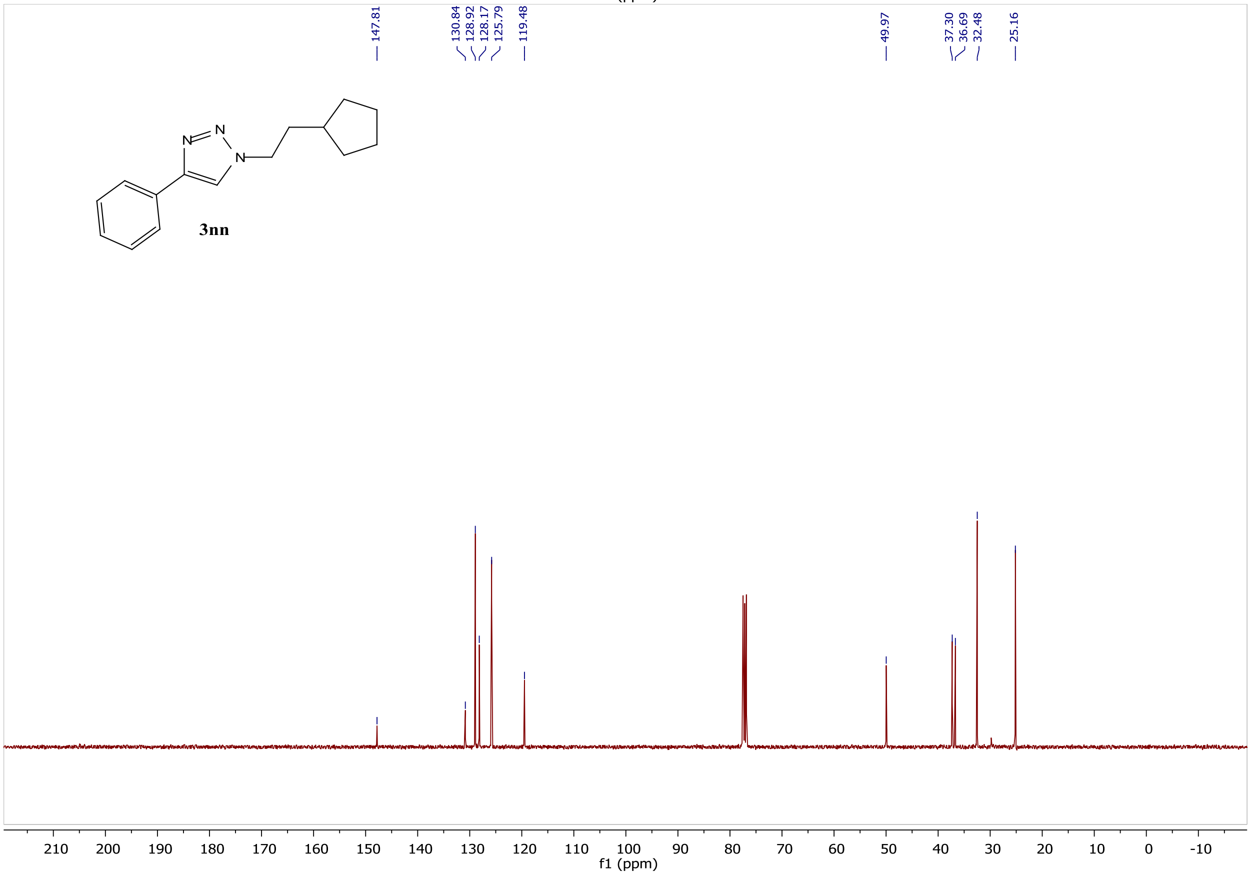
147.81
130.84 128.92 128.17 125.79 119.48
49.97
37.30 36.69 32.48
25.16
3nn
f1 (ppm)

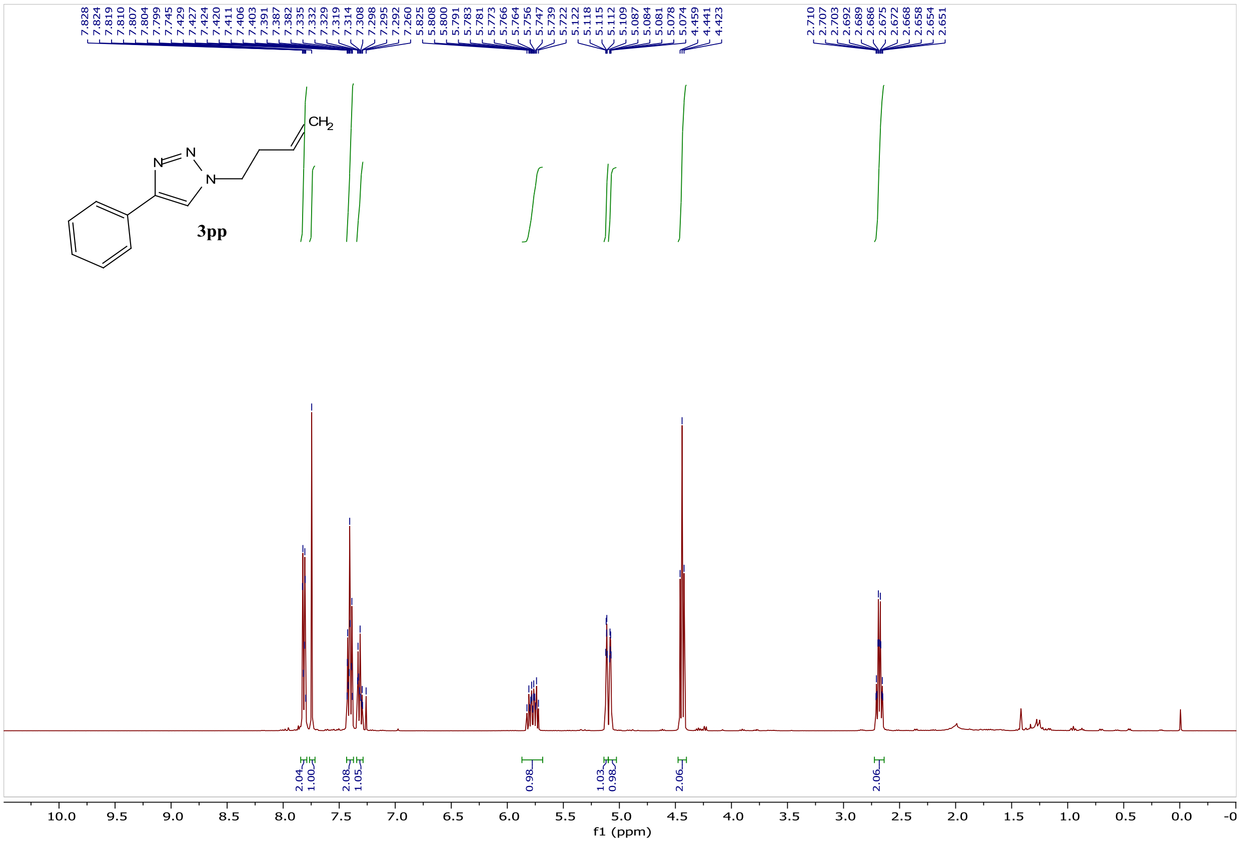
7.828 7.824 7.819 7.810 7.807 7.804 7.799 7.745 7.429 7.427 7.424 7.420 7.411 7.406 7.403 7.391 7.387 7.382 7.335 7.332 7.329 7.319 7.314 7.308 7.298 7.295 7.292 7.260 5.825 5.808 5.800 5.791 5.783 5.781 5.773 5.766 5.764 5.756 5.747 5.739 5.722 5.122 5.118 5.115 5.112 5.109 5.087 5.084 5.081 5.078 5.074 4.459 4.441 4.423
2.710 2.707 2.703 2.692 2.689 2.686 2.675 2.672 2.668 2.654 2.651
CH2
N
N
N
3pp
2.04 1.00 2.08 1.05 0.98 1.03 0.98 2.06 2.06
f1 (ppm)

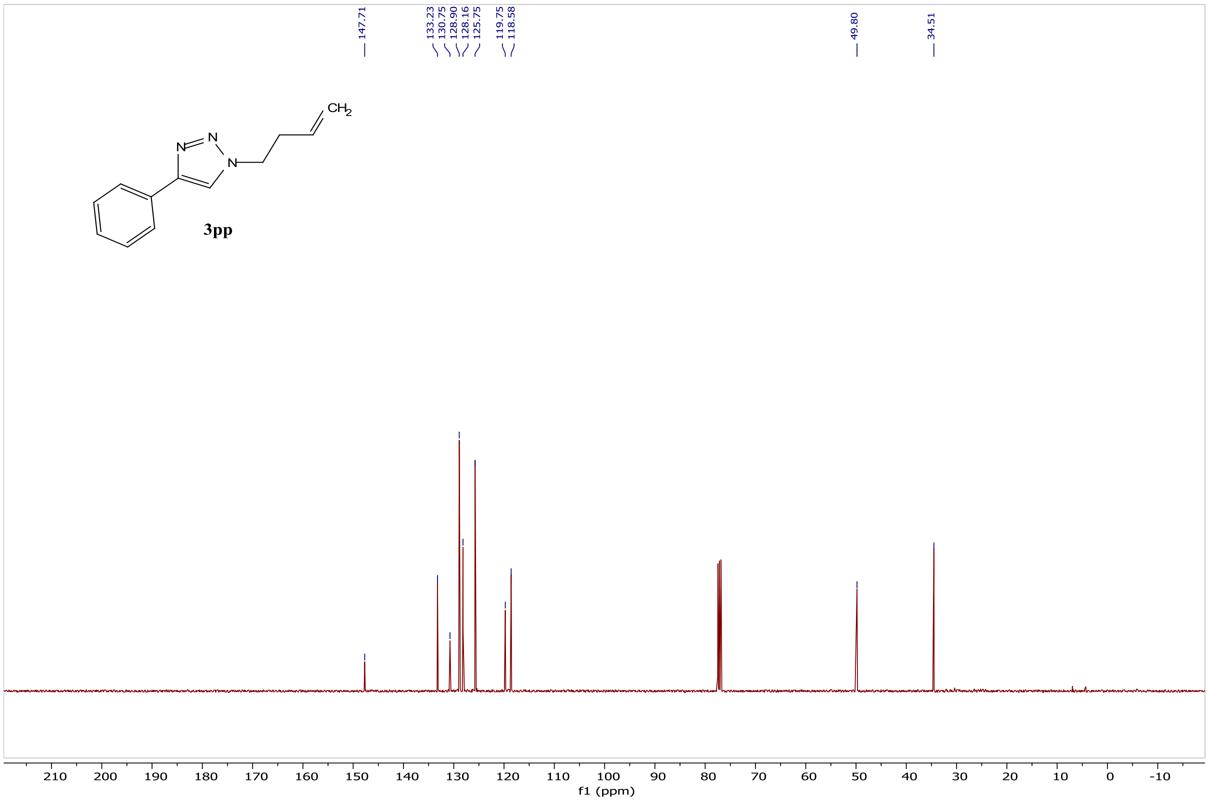
147.71
133.23 130.75 128.90 128.16 125.75 119.75 118.58
49.80
34.51
CH2
N
N
N
3pp
f1 (ppm)
